# Extraction solvent shapes the phytochemistry, multi-target bioactivity, and machine learning-predicted anticancer targets of *Seseli libanotis*

**DOI:** 10.1039/d6ra07396d

**Published:** 2026-08-28

**Authors:** Mehmet Veysi Cetiz, Abdullahi Ibrahim Uba, Milena Terzic, Josef Jeko, Zsolt Szilágyi, Ulku Yerebasan, Ismail Koyuncu, Ozgur Yuksekdag, Sakina Yagi, Gokhan Zengin

**Affiliations:** a Department of Medical Biochemistry, Faculty of Medicine, Harran University Sanliurfa 63290 Türkiye mvcetiz@gmail.com; b Department of Biostatistics and Medical Informatics, Faculty of Medicine, İstinye University Istanbul 34396 Türkiye abdullahi.iu2@gmail.com; c University of Novi Sad, Faculty of Technology Novi Sad Bulevar cara Lazara 1 21000 Novi Sad Serbia; d Science Park, University of Nyíregyháza Nyíregyháza Hungary jjozsi@gmail.com szilagyi.zsolt@nye.hu; e Bingol University, Faculty of Veterinary Medicine, Department of Pharmacology and Toxicology Bingöl 12400 Türkiye; f Department of Biology, Science Faculty, Khartoum University Khartoum Sudan sakinayagi@gmail.com; g Department of Biology, Science Faculty, Selcuk University, Campus Konya Türkiye

## Abstract

*Seseli libanotis* (L.) W. D. J. Koch (Apiaceae) is a wild vegetable traditionally consumed in eastern Anatolia, but its non-volatile phytochemistry and multi-target bioactivity remain insufficiently characterized. This study evaluated how extraction polarity shapes the chemical and biological profiles of four aerial-part extracts. The extracts were analyzed for total phenolic and flavonoid contents, chemical composition, antioxidant activity, inhibition of five enzymes, cytotoxicity against three tumor cell lines and human embryonic kidney (HEK-293) cells, and *in silico* molecular docking with enzyme targets. The ethanol/water extract showed the highest total phenolic content. Qualitative liquid chromatography-mass spectrometry (LC-MS) profiling showed broad coverage of caffeoylquinic acids and flavonoid glycosides in the polar extracts. Antioxidant activity was solvent- and assay-dependent: water was most active in the 2,2-diphenyl-1-picrylhydrazy (DPPH), cupric reducing antioxidant capacity (CUPRAC), and ferric reducing antioxidant power (FRAP) assays, ethanol/water in the 2,2′-azino-bis(3-ethylbenzothiazoline-6-sulfonic acid) (ABTS) assay, and ethyl acetate (EA) in the phosphomolybdenum assay. Ethanol and ethanol/water showed the highest acetylcholinesterase (AChE) and α-glucosidase inhibition, without significant differences between them; EA was most active against butyrylcholinesterase (BChE) and α-amylase. Molecular docking analyses suggested favorable binding modes of selected phytochemicals within the active sites of the investigated enzyme targets. Subsequent molecular dynamics simulations validated these interactions by demonstrating stable binding conformations sustained through persistent hydrogen-bonding networks. The ethanol/water extract was the most cytotoxic against three tumor cell lines (IC_50_ 31.77–41.25 µg mL^−1^). Annexin V and propidium iodide staining (Annexin V/PI) and acridine orange/ethidium bromide (AO/EB) staining indicated apoptosis-associated viability loss; however, similar activity against HEK-293 cells indicated limited tumor selectivity. Extraction solvent was the principal determinant of phytochemical composition and bioactivity, while the low selectivity highlights the need for fractionation and compound-level validation.

## Abstract 2

*Seseli libanotis* is a potential raw material for preparing health-promoting applications.

## Graphical abstract

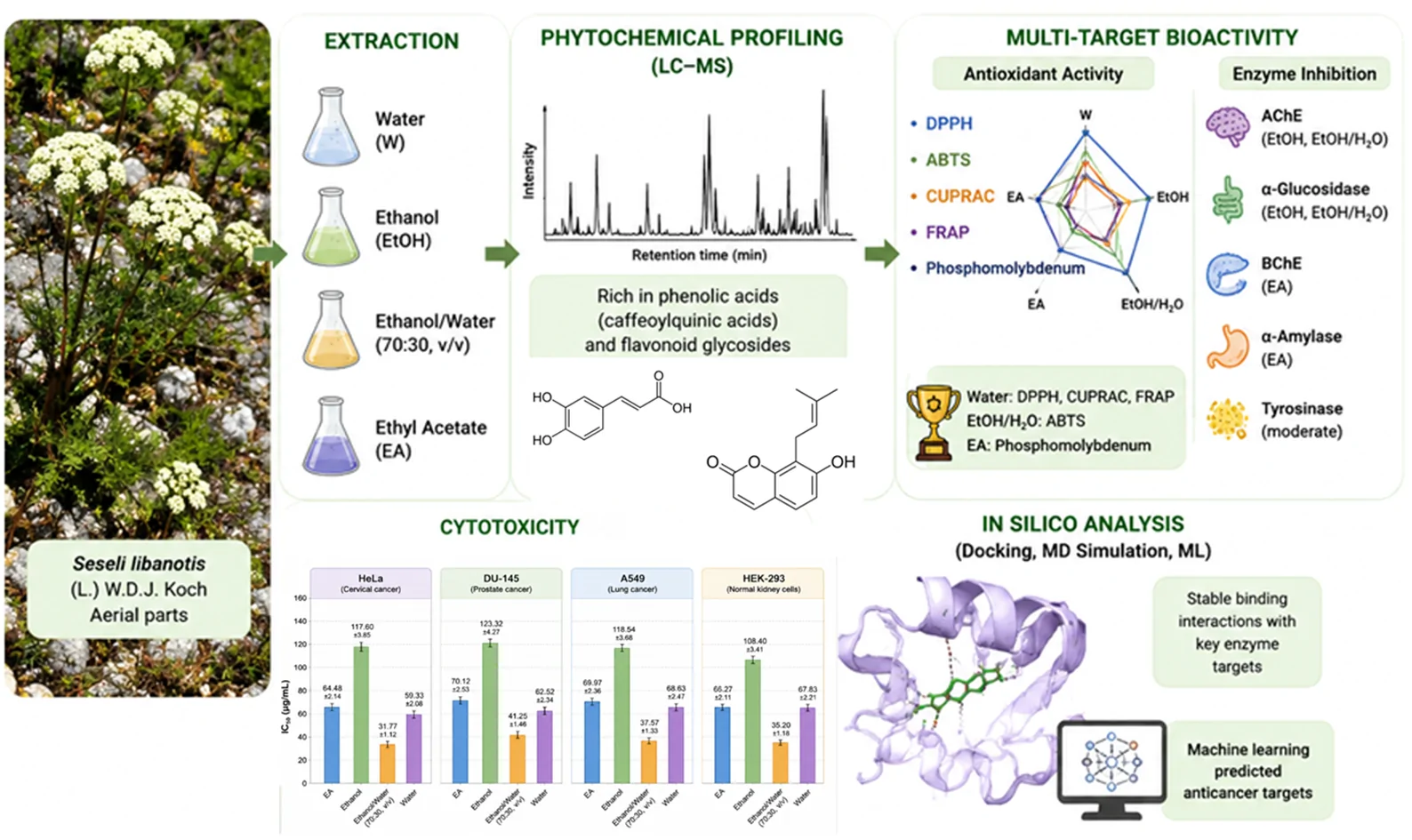

## Introduction

Apiaceae comprises a large family of aromatic and economically important plants widely used as foods, spices, and traditional remedies. Within this family, the Eurasian genus *Seseli* is chemically characterized primarily by essential oils and coumarins, including simple (osthenol, sesibiricin, sesebrin *etc.*) furano-, (xanthotoxin, bergapten, psoralen *etc*) and pyranocoumarins (pteryxin, xanthyletin derivative *etc*), together with phenolic acids (benzoic acid, gallic acid *etc*), flavonoids (seselinonol, apigenin, catechin *etc*), terpenoids (α-pinene, germacrene D, sabinene *etc*), and polyacetylenes (falcarinol, falcarindiol *etc*). These constituents have been associated with diverse biological activities, although the strength of the available evidence varies considerably among species, plant organs, extraction procedures, and experimental systems.^1–4^

For *Seseli libanotis* (L.) W. D. J. Koch, pharmacological research has focused predominantly on the volatile fraction and essential oil.^5–7^ By contrast, its non-volatile constituents, particularly polar phenolic compounds frequently associated with antioxidant and enzyme-modulating effects, remain comparatively poorly characterized. A solvent-resolved investigation combining phytochemical profiling with complementary biochemical, computational, and cell-based analyses may therefore provide valuable chemotaxonomic and pharmacological information.

*Seseli libanotis*, locally known as “Kelemkesir” or “Kelemenkesir”, is traditionally consumed as a vegetable in eastern Anatolia. Previous studies have examined the volatile composition and antibacterial activity of its aerial parts,^6^ the anti-inflammatory and antinociceptive effects of *Seseli* extracts,^8^ the phenolic content and antioxidant activity of methanolic extracts,^9^ the variability of its essential oil,^5^ and the antioxidant potential of Turkish *Seseli* species.^10^ More recently, the chemical composition, antibacterial activity, and cytotoxic effects of the root essential oil against HeLa cells were investigated,^11^ while the essential oil and *n*-hexane, ethyl acetate, and methanol extracts of the aerial parts were evaluated for antioxidant and tyrosinase-inhibitory activities.^7^ However, a systematic comparison linking extraction polarity and LC-MS-based profiling of the non-volatile fraction with antioxidant, enzyme-inhibitory, computational, and cell-based responses has not yet been reported.

A multi-assay strategy is essential when evaluating chemically complex plant extracts, since no single test can adequately capture their biological potential. Antioxidant assays differ in their underlying mechanisms and responsiveness to individual phytochemical classes, whereas enzyme-inhibition studies provide complementary, target-oriented evidence of functional activity. In the present study, the selected enzymes collectively represent biological processes associated with cholinergic dysfunction, postprandial glucose regulation, melanogenesis, and enzymatic browning. Their evaluation alongside multiple antioxidant assays therefore enables a broader and more mechanistically informative assessment of extract activity than would be possible using isolated endpoints alone.^12–14^

Interpreting the activity of crude extracts nevertheless remains challenging because the observed effects arise from complex mixtures in which individual constituents may act additively, synergistically, or antagonistically. Molecular docking was therefore incorporated as a complementary tool to bridge phytochemical profiling and experimental enzyme inhibition. By examining the predicted binding orientations, relative binding strengths, and key interactions of selected LC-MS-identified compounds within enzyme active sites, the *in silico* analysis provided a molecular framework for interpreting the observed inhibitory patterns. Although docking predictions cannot establish biological activity on their own, they can highlight plausible compound–target relationships and help prioritise candidates for isolation, mechanistic investigation, and experimental validation.

Accordingly, ethyl acetate, ethanol, ethanol/water, and water extracts of the aerial parts of *S. libanotis* were compared with respect to their total phenolic and flavonoid contents, qualitative LC-MS composition, antioxidant activity assessed using six complementary assays, and inhibitory effects against five enzymes. Cytotoxicity and cell-death responses were also evaluated in three tumour cell lines and a non-cancerous reference cell line, while molecular docking was used to investigate the potential interactions of selected LC-MS-identified constituents with the studied enzyme targets.

To the best of our knowledge, this study provides the first integrated, solvent-resolved characterisation of the non-volatile phytochemical fraction of *S. libanotis* combining LC-MS profiling with multi-target antioxidant and enzyme-inhibitory assays, molecular docking, and cell-based analyses. The principal objective was to determine how extraction polarity influences chemical coverage and biological response and thereby identify the extracts and compounds that warrant further fractionation, isolation, quantitative analysis, and experimental validation.

## Materials and methods

### Plant collection

Plant samples were collected in 2025 from Harput (Şüsnaz village), Elazıg, Turkey. Dr Ugur Cakilcioglu officially carried out the botanical identification. A reference specimen bearing the code UC-2023-15 was placed at the Munzur University herbarium. The aerial parts were separated immediately after collection and allowed air to dry at room temperature in a well-ventilated, shady area. The plant material was pulverized into a fine powder once it had dried completely. The powdered samples were kept under constant conditions in light-proof containers to maintain chemical stability and prevent spoiling.

### Plant extract preparation

To separate bioactive chemicals, four different solvents with different polarities were used. Distilled water, ethanol/water (70%), ethanol, and ethyl acetate. Ten grams of dried plant material were mixed with two hundred milliliters of each solvent. Depending on the type of solvent used, the extraction process was different. The plant material was macerated by soaking it in the organic solvents for a whole day at room temperature. Instead, a 15 minute hot-water infusion was used for water-based extraction. Following extraction, the organic solvent extracts were concentrated under low pressure using a rotary evaporator, and the water extract was processed by freeze-drying. Extraction yields (%) are given in Table 1.

**Table 1:** Extraction yields (%), total phenolic and flavonoid contents^a^ of *Seseli libanotis* aerial-part extracts

Extracts	Extraction yields (%)	TPC^b^ (mg GAE per g)	TFC^c^ (mg RE per g)
EA	1.80	28.48 ± 0.76^d^	1.17 ± 0.54^d^
Ethanol	8.44	36.37 ± 0.65^c^	37.40 ± 0.88^a^
Ethanol/water	26.77	60.27 ± 0.82^a^	32.30 ± 0.23^b^
Water	23.65	56.28 ± 0.58^b^	22.31 ± 1.14^c^

a Values are presented as mean ± SD of three independent measurements (*n* = 3). Means within the same column followed by different superscript letters (a–d) differ significantly at *p* ≤ 0.05.

b mg gallic acid equivalents per gram of extract.

c mg rutin equivalents per gram of extract.

### Total phenolic and flavonoid contents

Total phenolic^15^ and total flavonoid^16^ contents in the extracts were determined using validated colorimetric methods, following established procedures. Calibration curves were prepared with gallic acid for phenolics and rutin for flavonoids, enabling the expression of the results as GAE and RE per gram, respectively.

### Phytochemical analysis by HPLC-ESI-Orbitrap-MS/MS

The liquid chromatographic system was a Dionex 3000RS Ultimate system, where separations were achieved on a Thermo Accucore C18 (100 mm × 2.1, mm i.d., 2.6 µm) column thermostated at 25 °C (±1 °C). A previously developed and successfully applied method was used.^17^ All analytical details are given in the SI.

### Antioxidant assays

A set of *in vitro* assays was employed to evaluate antioxidant activity, following the protocol described by ref. 18. Reducing power was determined using the FRAP and CUPRAC methods, while radical scavenging capacity was assessed by DPPH and ABTS assays. The outcomes of these four tests were converted into Trolox equivalents and reported as mg TE per gram of dried extract. In addition, total antioxidant capacity was measured using the phosphomolybdenum (PBD) assay (mmol TE/g), and metal chelating activity was expressed as mg EDTA equivalents per gram (mg EDTAE/g).

### Enzyme inhibitory activity assays

Following established colorimetric methods,^18^ the extracts were evaluated for their ability to inhibit five key enzymes: acetylcholinesterase (AChE), butyrylcholinesterase (BChE), tyrosinase, α-amylase, and α-glucosidase. To ensure comparability among samples, inhibition results were expressed relative to appropriate reference compounds. Accordingly, cholinesterase inhibition was reported as mg GALAE per g, α-amylase and α-glucosidase inhibition as mmol ACAE per g, and tyrosinase inhibition as mg KAE per g.

### Cell culture

Four ATCC-derived lines were used: DU-145 (prostate carcinoma), HeLa (cervical adenocarcinoma), A549 (lung adenocarcinoma), and HEK-293 (normal human embryonic kidney cells, serving as the control line). Stocks were stored in liquid nitrogen and thawed as needed. Growth medium was DMEM-F12 or RPMI-1640 with 10% FBS, 100 µg mL^−1^ streptomycin, and 100 IU per mL penicillin. Cells were kept at 37 °C in a humidified incubator with 5% CO_2_.

### Cell viability assay

MTT assay was used to measure cytotoxicity. Cells were seeded at 1 × 10^4^ per well in 96-well plates and given 24 hours to attach. Medium was then swapped for extract at concentrations of 0 to 200 µg mL^−1^ for another 24 hours. Each well received 10 µL of MTT (0.5 mg mL^−1^) and sat for 4 hours before the medium was removed and replaced with 100 µL DMSO per well to dissolve the formazan crystals. Plates were read at 570/690 nm on a Thermo Multiskan GO reader, and IC_50_ values were pulled from the dose–response curves.

### AO/EB staining for apoptosis in HeLa cells

HeLa cells treated with ethanol/water extract (30 µg mL^−1^) were checked for apoptotic morphology by AO/EB staining. After treatment, cells went through a PBS wash, fixation in 70% ethanol, and a rinse in distilled water. Staining used the AO/EB working solution (Sigma-Aldrich, Cat. no. A6014-E1510), and images were taken under a fluorescence microscope.

### Annexin V assay for apoptosis in HeLa cells

A second apoptosis check used the FITC Annexin V Apoptosis Detection Kit I (BD Biosciences). HeLa cells were seeded at 5 × 10^5^ per well in 6-well plates and left for 24 hours. They were then treated with the extract (30 µg mL^−1^) for another 24 hours. Cells were trypsinized and resuspended in 1× binding buffer, then moved into flow cytometry tubes. Each tube got 5 µL FITC-Annexin V and 5 µL propidium iodide. After 15 minutes at room temperature in the dark, 100 µL of 1× binding buffer was added. Samples were centrifuged, given 5 more minutes, and run on a BD flow cytometer.

### Network pharmacology

Canonical SMILES of the selected phytochemicals were obtained from PubChem.^19^ Compound-associated target genes were predicted using a local ChEMBL multitask model-based target prediction workflow. SMILES were standardized with RDKit and converted into Morgan fingerprints.^20^ The fingerprints were then submitted to the ChEMBL multitask ONNX model, and predicted ChEMBL target IDs were mapped to target names, official gene symbols, UniProt IDs, target types, and organism information using ChEMBL v36.^21,22^ Predictions with scores ≥0.50 were retained, with a maximum of 50 targets per compound. Only human targets with mapped gene symbols were used for downstream term-specific intersection analysis. Disease-related gene sets were obtained from the GeneCards database (https://www.genecards.org/) separately for cell death, necroptosis, and apoptosis. For each term were filtered as protein-coding. In all term modules, only genes with a GeneCards relevance score ≥15 were included. Interaction relationships among the overlapping genes were explored through the STRING database v12.0 (https://string-db.org/).^23^ An interaction score threshold of 0.4 was selected to balance network coverage and confidence. The interaction network was subsequently examined and visualized in Cytoscape v3.10.3 for further topological assessment.^24^

### Compound–target network

Term-specific compound–target and PPI networks were generated for the overlapping targets. Predicted ligand–target pairs were filtered according to the shared genes between CMT/ChEMBL-predicted human targets and GeneCards-derived cancer genes and visualized in Cytoscape v3.10.3 as bipartite compound–target networks. In these networks, compounds and target genes were shown as distinct node classes, and edges represented predicted ligand–target associations. In parallel, the overlapping genes of each cancer module were submitted to STRING v12.0 using *Homo sapiens* as the organism and an interaction score threshold of 0.4. No additional interactors were added. The resulting PPI networks were exported and visualized in Cytoscape v3.10.3 to examine the functional connectivity among cancer-specific candidate targets.^21–24^

### Functional annotation of common genes

Functional annotation and enrichment of the overlapping targets were performed with ToppFun. We queried Gene Ontology (GO) categories, focusing on Biological Process (BP), alongside Kyoto Encyclopedia of Genes and Genomes (KEGG) pathway enrichment. Terms and pathways reaching *p* < 0.05 were considered significant.^25^ To emphasize the most informative categories, we selected the top 5 GO BP terms and top 10 KEGG pathways ranked by gene count and visualized them as bar plots.^26^ All figures were generated in R v4.3.3.

### External transcriptomic validation

External transcriptomic validation was performed using GEO datasets corresponding to the cancer models evaluated in the cytotoxicity experiments: GSE63514 for cervical cancer, GSE19804 for lung cancer, and GSE46602 for prostate cancer. Only samples clearly annotated as tumor or normal/non-tumor control were retained, whereas ambiguous, precancerous, dysplastic, intermediate, or otherwise non-relevant samples were excluded. The final datasets included 28 tumor and 24 control samples for cervical cancer, 60 tumor and 60 control samples for lung cancer, and 36 tumors and 14 control samples for prostate cancer, resulting in a total of 222 samples. Probe-level microarray data were processed in R using GEOquery/Bioconductor, mapped to official gene symbols using the corresponding Affymetrix GPL570 platform annotation, and averaged when multiple probes mapped to the same gene.^27^ The requirement for log_2_ transformation was assessed from the expression-value distributions; no additional log_2_ transformation was applied to the final expression matrices. For each cancer type, the same predefined panel of 34 regulated-cell-death-associated targets identified through the network pharmacology analysis was used as the input feature set. All 34 predefined target genes were successfully mapped in each of the three GEO expression matrices, and the feature panel was not re-selected according to the individual GEO results. Tumor-control expression differences were assessed separately for each cancer dataset using the limma package, followed by Benjamini–Hochberg false-discovery-rate correction.^28,29^

### Machine-learning analysis

Machine-learning analysis was performed in R using the caret package with ElasticNet logistic regression.^30,31^ For each cancer dataset, samples were divided into stratified training and held-out test subsets at an approximately 80 : 20 ratio, and model tuning was performed by repeated cross-validation using only the training data. Final test-set performance was assessed using ROC–AUC, accuracy, balanced accuracy, sensitivity, specificity, *F*1 score, and held-out prediction results. ElasticNet feature importance was used to interpret the relative contribution of genes within the predefined 34-gene signature and was not considered an independent target-discovery procedure. The complete sample metadata, gene-level differential-expression results, held-out test predictions, model-performance metrics, and feature-importance results are provided in the SI.

### Molecular docking analysis

A total of 19 phytochemicals were selected for molecular docking based on their identification/annotation in *S*. *libanotis* extracts and the availability of reliable canonical SMILES data for ligand preparation. The selected target proteins, PDB IDs, binding-pocket coordinates, and docking grid parameters are summarized in Table S1. Docking ligands were prepared from canonical SMILES using Gypsum-DL. Ligand-state generation was performed at physiological pH 7.4, and initial 3D structures were generated in SDF format. The generated SDF structures were checked using RDKit. Geometry optimization was performed using the MMFF94 force field when suitable parameters were available, with a maximum of 2000 iterations. For each ligand, the lowest-energy valid conformer was retained. The optimized ligands were then converted into AutoDock Vina-compatible PDBQT format using Meeko. During conversion, hydrogen atoms, Gasteiger charges, AutoDock atom types, rotatable-bond definitions, torsion trees, and PDBQT-compatible ligand parameters were assigned. Energy-minimized ligands were converted to AutoDock Vina-compatible PDBQT format using Meeko. Gastric cancer-related protein targets were retrieved from the Protein Data Bank.^32,33^ Molecular docking was performed using AutoDock Vina v1.1.2 software,^34–36^ with exhaustiveness set to 32. The binding pockets of the target proteins were predicted using the POCASA v1.1 server, and docking grid boxes were centered on the predicted pocket regions. Molecular docking simulations were performed using AutoDock Vina v1.1.2, with exhaustiveness set to 32. Protein–ligand interactions were analyzed using the Protein–Ligand Interaction Profiler (PLIP).^37,38^

### Molecular dynamics simulation

Following the docking stage, MD simulations were performed to probe how stable and conformationally well-behaved the chosen protein–ligand complexes were over time. Systems were first built using the CHARMM-GUI web server,^39^ with protein and ligand parameters assigned from the CHARMM36m force field and the surrounding aqueous environment modeled explicitly with TIP3P water. Physiological ionic strength was approximated by adding 0.15 M NaCl together with the counterions needed to neutralize each system.

Short-range non-bonded interactions were handled with the Verlet cutoff scheme, hydrogen-containing covalent bonds were constrained *via* LINCS, and long-range electrostatics were computed using the particle mesh Ewald (PME) approach. Prior to equilibration, every system underwent energy minimization by the steepest-descent method until the maximum force fell below 1000 kJ mol^−1^ nm^−1^, clearing steric clashes and unfavorable contacts.^40,41^

Equilibration then proceeded in two stages: an NVT phase followed by an NPT phase, both held at 310 K, after which 100 ns production runs were carried out in GROMACS (v2023).^42,43^ The resulting trajectories were subsequently examined for structural stability, per-residue flexibility, solvent exposure, persistence of hydrogen bonding, and the dynamics of ligand–protein contacts throughout the simulation.

### Statistical analysis

Experiments were performed in triplicate. Statistical differences between extract samples were evaluated by one-way ANOVA with Tukey's post hoc test (GraphPad Prism v9.2), using *p* < 0.05 as the threshold for significance.

## Results and discussion

### Total phenolic (TPC) and flavonoids (TFC) contents

The total phenolic (TPC) and total flavonoid contents (TFC) of the four extracts are presented in Table 1. TPC was favoured by water-containing extraction systems and reached its highest value in the ethanol/water extract (60.27 mg GAE per g), followed by the water (56.28 mg GAE per g), ethanol (36.37 mg GAE per g), and EA extracts (28.48 mg GAE per g). In contrast, TFC was highest in the ethanol extract (37.40 mg RE per g), followed by ethanol/water (32.30 mg RE per g), water (22.31 mg RE per g), and EA (1.17 mg RE per g). The different rankings indicate that the two assays reflect overlapping but chemically distinct groups of extractable constituents.

This divergence is partly explained by the analytical principles of the methods. The Folin–Ciocalteu reagent is not exclusively selective for phenolic compounds and may also react with other reducing substances, including ascorbic acid, reducing sugars, and certain amino acids. TPC should therefore be interpreted as an operational estimate influenced by the overall reducing capacity of the extract rather than as an absolute measure of phenolic concentration.^44^ Similarly, the aluminium chloride assay is structurally selective and responds more strongly to particular flavonoid subclasses and hydroxylation patterns, while other flavonoids may be detected only weakly.^45^ The divergence between TPC and TFC is therefore not contradictory but reflects the different chemical selectivity of the two assays.

The colorimetric results were broadly consistent with the qualitative LC-MS profiles presented in Table 2. The ethanol/water and water extracts contained numerous caffeoylquinic acids and other highly polar phenolic constituents, which may have contributed substantially to their Folin–Ciocalteu responses without producing a proportionally high flavonoid-specific signal. By comparison, the ethanol extract contained several quercetin-, kaempferol-, and isorhamnetin-derived glycosides, in agreement with its comparatively high TFC. The EA extract showed a markedly different pattern, combining a measurable Folin–Ciocalteu response with a very low TFC and an LC-MS profile distinguished by a broader representation of relatively lipophilic constituents, including long-chain fatty acids and chlorophyll-derived compounds. Together, these findings indicate that solvent polarity strongly influenced the relative distribution of phenolic acids, flavonoids, coumarins, and other reducing compounds among the extracts. However, because the LC-MS analysis was qualitative, direct quantitative relationships between individual constituents and the colorimetric values cannot be established.

**Table 2:** Tentatively annotated constituents in the tested *Seseli libanotis* extracts based on qualitative LC-MS analysis (+, detected; −, not detected)

Compounds	Formula	[M + H]^+^	[M − H]^−^	Ethyl acetate	EtOH	EtOH/water	Water	References
Phloroglucinol^a^	C_6_H_6_O_3_	127.040		—	+	+	—	
Quinic acid^a^	C_7_H_12_O_6_		191.06	+	+	+	+	
Shikimic acid^a^	C_7_H_10_O_5_		173.05	+	+	+	+	
Citric acid^a^	C_6_H_8_O_7_		191.02	—	+	+	+	
Gentisic acid-*O*-glucoside	C_13_H_16_O_9_		315.07	—	+	+	+	
Neochlorogenic acid (3-*O*-caffeoylquinic acid)^a^	C_16_H_18_O_9_	355.10		—	+	+	+	
Uralenneoside	C_12_H_14_O_8_		285.06	—	+	+	+	
Salicylic acid-2-*O*-glucoside	C_13_H_16_O_8_		299.08	—	+	+	+	
3-*O*-(*p*-Coumaroyl)quinic acid	C_16_H_18_O_8_		337.09	—	+	+	+	
Penstemide	C_21_H_32_O_10_		443.19	—	+	+	+	
*trans*-4-*O*-Glucosyl-4-hydroxycinnamate	C_15_H_18_O_8_		325.09	—	+	+	+	
Caffeic acid-4-*O*-glucoside	C_15_H_18_O_9_		341.09	—	+	+	+	
Chlorogenic acid (5-*O*-caffeoylquinic acid)^a^	C_16_H_18_O_9_	355.10		+	+	+	+	
3-*O*-Feruloylquinic acid	C_17_H_20_O_9_		367.10	—	+	+	+	
Caffeic acid^a^	C_9_H_8_O_4_		179.03	—	+	+	+	
Cryptochlorogenic acid (4-*O*-caffeoylquinic acid)^a^	C_16_H_18_O_9_	355.10		—	+	+	+	
Asperulosidic acid	C_18_H_24_O_12_	433.13		—	+	+	+	
5-*O*-(*p*-Coumaroyl)quinic acid	C_16_H_18_O_8_		337.09	—	+	+	+	
Apo-9-zeaxanthinone or isomer	C_13_H_20_O_2_	209.15		+	+	+	+	
6-*O*-(*p*-Coumaroyl)-1-*O*-glyceroylglucose	C_18_H_22_O_11_		413.11	—	+	+	+	
5-*O*-Feruloylquinic acid	C_17_H_20_O_9_		367.10	+	+	+	+	
*p*-Coumaric acid^a^	C_9_H_8_O_3_		163.04	+	+	+	+	
Scopoletin (7-hydroxy-6-methoxycoumarin)^a^	C_10_H_8_O_4_	193.05		—	+	—	—	
Hexosyl-hydroxycinnamate	C_15_H_18_O_8_		325.09	—	+	+	+	
5-*O*-(*p*-Coumaroyl)quinic acid cis isomer	C_16_H_18_O_8_		337.09	—	+	+	+	
Ferulic acid^a^	C_10_H_10_O_4_		193.05	—	+	+	—	
Quercetin-*O*-hexosylhexoside	C_27_H_30_O_17_		625.14	—	+	+	+	
5-*O*-Feruloylquinic acid cis isomer	C_17_H_20_O_9_		367.10	—	+	+	+	
Apigenin-C-arabinoside-C-glucoside	C_26_H_28_O_14_	565.16		—	+	+	+	
Vicenin-1 (apigenin-8-C-glucoside-6-C-xyloside)^a^	C_26_H_28_O_14_	565.16		—	+	+	+	
Ethyl chlorogenate	C_18_H_22_O_9_		381.12	—	+	+	—	
Isorhamnetin-*O*-hexosylhexoside	C_28_H_32_O_17_		639.16	—	+	+	+	
Di-*O*-caffeoylquinic acid isomer 1	C_25_H_24_O_12_		515.12	—	+	+	+	
Hyperoside (hyperin, Quercetin-3-*O*-galactoside)^a^	C_21_H_20_O_12_		463.09	+	+	+	+	
Quercetin-*O*-rhamnosylhexoside	C_27_H_30_O_16_		609.15	+	+	+	+	
Quercetin-3-*O*-glucuronide (miquelianin)	C_21_H_18_O_13_		477.07	—	+	+	+	
Di-*O*-caffeoylquinic acid isomer 2	C_25_H_24_O_12_		515.12	—	+	+	—	
Quercetin-3-*O*-(6″-malonyl)glucoside	C_24_H_22_O_15_		549.09	—	+	+	+	
Kaempferol-*O*-rhamnosylhexoside	C_27_H_30_O_15_		593.15	+	+	+	+	
Quercitrin (quercetin-3-*O*-rhamnoside)^a^	C_21_H_20_O_11_		447.09	+	+	+	+	
Quercetin-*O*-(acetyl)rhamnosylhexoside isomer 1	C_29_H_32O17_		651.16	—	+	+	+	
Kaempferol-3-*O*-rutinoside (nicotiflorin)	C_27_H_30_O_15_		593.15	+	+	—	+	
Kaempferol-3-*O*-glucuronide	C_21_H_18_O_12_		461.07	+	+	+	+	
Quercetin-*O*-(acetyl)rhamnosylhexoside isomer 2	C_29_H_32_O_17_		651.16	—	+	+	+	
Quercetin-*O*-(acetyl)rhamnosylhexoside isomer 3	C_29_H_32_O_17_		651.16	—	—	+	+	
Kaempferol-*O*-(malonyl)glucoside	C_24_H_22_O_14_		533.09	—	+	+	+	
Kaempferol-*O*-(acetyl)rhamnosylhexoside isomer 1	C_29_H_32_O_16_		635.16	—	+	+	+	
Isorhamnetin-*O*-rhamnoside	C_22_H_22_O_11_		461.11	+	+	+	+	
Quercetin (3,3′,4′,5,7-pentahydroxyflavone)	C_15_H_10_O_7_		301.04	—	+	+	—	
Kaempferol-*O*-(acetyl)rhamnosylhexoside isomer 2	C_29_H_32_O_16_		635.16	+	+	+	+	
Quercetin-*O*-caffeoylrhamnoside	C_30_H_26_O_14_		609.12	+	+	+	+	
Unidentified terpenoid isomer 1	C_15_H_18_O_3_		245.12	—	+	+	+	
Quercetin-*O*-(*p*-coumaroyl)rhamnoside	C_30_H_26_O_13_		593.13	+	+	+	+	
4-Hydroxyphenylethyl *trans*-ferulate	C_18_H_18_O_5_		313.11	+	+	+	—	
Isorhamnetin-*O*-(*p*-coumaroyl)hexoside	C_31_H_28_O_14_		623.14	—	+	+	—	
Malyngic acid (9,12,13-trihydroxy-10*E*,15*Z*-octadecadienoic acid)	C_18_H_32_O_5_		327.22	+	+	+	+	
Isorhamnetin-*O*-(*p*-coumaroyl)rhamnoside	C_31_H_28_O_13_		607.15	—	+	+	+	
Pinellic acid (9,12,13-trihydroxy-10*E*-octadecenoic acid)	C_18_H_34_O_5_		329.23	+	+	+	—	
Osthenol	C_14_H_14_O_3_	231.10		+	+	+	—	79
Stearidonic acid	C_18_H_28_O_2_		275.20	+	—	—	—	
Unidentified terpenoid isomer 2	C_15_H_18_O_3_			—	+	+	—	
Unidentified terpenoid isomer 3	C_15_H_18_O_3_			—	+	+	—	
9-Hydroxyoctadecadienoic acid	C_18_H_32_O_3_		295.23	+	+	+	+	
Unidentified saponin	C_30_H_48_O_2_	441.37		+	+	+	—	
α-Linolenic acid^a^	C_18_H_30_O_2_		277.22	+	+	+	—	
Hexadecenoic acid	C_16_H_30_O_2_		253.22	+	—	—	—	
Pentadecanoic acid	C_15_H_30_O_2_		241.22	+	—	—	—	
Palmitoleic acid	C_16_H_30_O_2_		253.22	—	—	+	—	
Linoleic acid^a^	C_18_H_32_O_2_		279.23	+	+	—	+	
Oleic acid^a^	C_18_H_34_O_2_		281.25	+	—	—	—	
Pheophorbide A	C_35_H_36_N_4_O_5_	593.28		+	+	+	—	
Arachidic acid (icosanoic acid)^a^	C_20_H_40_O_2_		311.30	+	—	—	—	
Behenic acid (docosanoic acid)^a^	C_22_H_44_O_2_		339.33	+	—	—	—	
Tricosanoic acid	C_23_H_46_O_2_		353.34	+	—	—	—	
Lignoceric acid (tetracosanoic acid)	C_24_H_48_O_2_		367.36	+	—	—	—	
Hyenic acid (pentacosanoic acid)	C_25_H_50_O_2_		381.37	+	—	—	—	
Cerotic acid (hexacosanoic acid)	C_26_H_52_O_2_		395.39	+	—	—	—	
Carboceric acid (heptacosanoic acid)	C_27_H_54_O_2_		409.40	+	—	—	—	
Melissic acid (triacontanoic acid)	C_30_H_60_O_2_		451.45	+	—	—	—	
Pheophytin A	C_55_H_74_N_4_O_5_	871.57		+	+	—	—	

a Confirmed by standard.

Comparison with previous studies should be interpreted cautiously because reported TPC values are influenced by plant origin and taxonomic status, the analysed organ, extraction solvent and protocol, solid-to-solvent ratio, extract yield, calibration standard, and the basis used for expressing the results. Within these limitations, the values obtained in the present study were higher than those reported for *S. gummiferum* and *S. transcaucasicum* (19.09–24.33 mg GAE per g; Zengin *et al.*^46^), but lower than those reported for methanolic extracts of Serbian *S. libanotis* (84.04–87.52 mg GAE per g; Matejić *et al.*^9^). Rather than indicating analytical inconsistency, this variation most likely reflects the combined influence of botanical, geographical, and methodological factors on the apparent phenolic content of *Seseli* extracts.

### HPLC-ESI-Orbitrap-MS/MS results

**Fig. 1:**
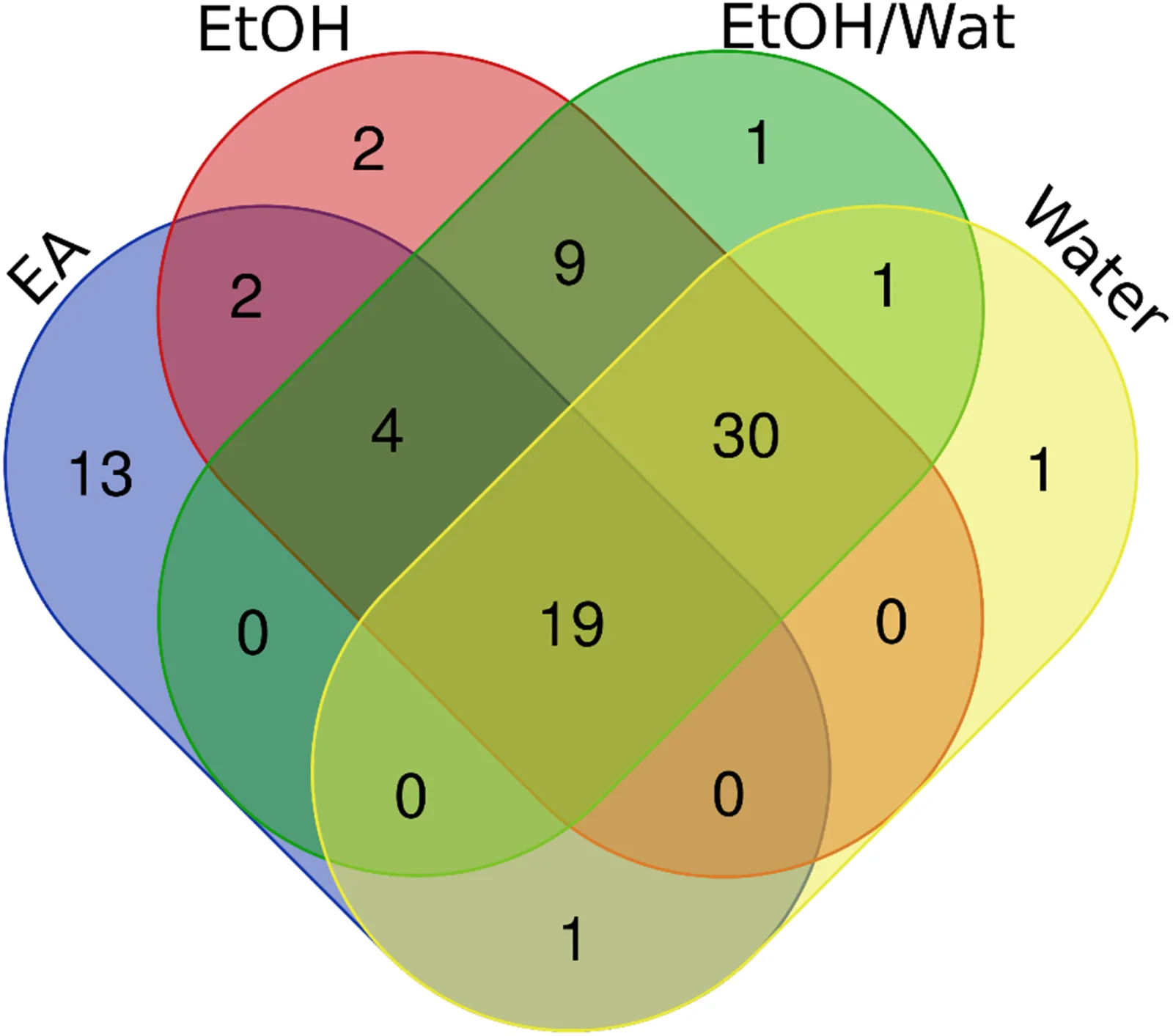
Veen diagram based on the numbers of identified compounds in the tested extracts.

The qualitative HPLC-ESI-Orbitrap-MS/MS dataset comprised 80 tentatively annotated constituents spanning organic acids, hydroxycinnamic acids and acyl-quinic acid derivatives, flavonoids, coumarins, terpenoid-related compounds, oxylipins, chlorophyll-derived compounds, and fatty acids (Table 2). Based on presence/absence data, the broadest chemical coverage was observed in the ethanol and ethanol/water extracts, with 66 and 64 annotated constituents, respectively, followed by the water extract with 52 and the EA extract with 39 (Fig. 1). These numbers reflect the diversity of compounds detected under the applied analytical conditions and should not be interpreted as measures of their relative or absolute abundance.

The principal differences among the extracts concerned the distribution of phytochemical classes rather than simply the total number of annotations. The ethanol, ethanol/water, and water extracts showed similarly broad coverage of flavonoid glycosides, each containing almost the complete set of compounds assigned to this class. Ethanol and ethanol/water also contained a particularly broad range of hydroxycinnamic acids and acyl-quinic acid derivatives. Although the water extract retained most polar phenolic acids and flavonoid glycosides, it contained fewer terpenoid-related compounds, oxylipins, chlorophyll derivatives, and long-chain fatty acids. The EA extract contained a smaller subset of the polar phenolics but was chemically distinguished by a broader representation of lipophilic constituents. Notably, a homologous series of saturated C20–C30 fatty acids, including arachidic, behenic, tricosanoic, lignoceric, hyenic, cerotic, carboceric, and melissic acids, was detected exclusively in EA. The solvents therefore recovered chemically complementary groups of constituents rather than progressively richer versions of a single phytochemical profile.

Among the polar phenolics, tentative assignments included neochlorogenic acid (3-*O*-caffeoylquinic acid; 3-CQA), chlorogenic acid (5-*O*-caffeoylquinic acid; 5-CQA), cryptochlorogenic acid (4-*O*-caffeoylquinic acid; 4-CQA), two dicaffeoylquinic acid isomers, feruloyl- and *p*-coumaroylquinic acid derivatives, and free caffeic, *p*-coumaric, and ferulic acids. The revised acyl-quinic acid nomenclature was applied according to Abrankó and Clifford.^47^ Flavonoid annotations comprised derivatives of quercetin, kaempferol, isorhamnetin, and apigenin. These included hyperoside, quercitrin, miquelianin, nicotiflorin, vicenin-1, and several putatively acetylated, malonylated, caffeoylated, or coumaroylated glycosides. Although the broad distribution of these compounds across the polar extracts was similar, several differences were evident. For example, quercetin aglycone and ethyl chlorogenate were detected in the ethanol and ethanol/water extracts but not in the water or EA extracts, whereas individual glycosides showed extract-specific patterns.

The tentative annotations were supported by established mass-spectrometric fragmentation behaviour reported for the corresponding phytochemical classes. Acyl-quinic acid derivatives can be differentiated through characteristic precursor and production patterns, while flavonoid O- and C-glycosides are commonly characterised from diagnostic neutral losses, aglycone and radical-aglycone ions, and cross-ring fragmentation pathways.^48–55^ Nevertheless, because co-chromatographic confirmation with authentic standards was not available for all constituents, the assignments should be regarded as tentative. Structural confidence is particularly limited for compounds reported as positional isomers, acylated glycosides, and unidentified terpenoid-related signals. Moreover, the qualitative nature of Table 2 precludes conclusions regarding compound concentration, extract enrichment, or the relative contribution of individual constituents to the measured biological activities.

Despite these limitations, the HPLC-ESI-Orbitrap-MS/MS profiles provide a relevant chemical context for the antioxidant results. Caffeoylquinic acids and flavonoid glycosides were widely detected in the polar extracts, which generally showed stronger radical-scavenging and reducing activities. Chlorogenic and related caffeoylquinic acids are widely distributed dietary phenolics with documented antioxidant and anti-inflammatory properties.^56–58^ Similarly, quercetin-derived compounds commonly possess an *ortho*-dihydroxy or catechol arrangement in the B-ring, a structural feature associated with electron donation and radical stabilisation.^59^ However, the presence of such compounds cannot by itself explain the differences among the antioxidant assays, since the measured responses may reflect their concentrations, structural diversity, interactions with other constituents, and the distinct chemical mechanisms of each assay.

The annotated profiles also provide plausible, but not conclusive, links to the enzyme-inhibition results. Quercetin, kaempferol, isorhamnetin, and their glycosides belong to flavonoid subclasses frequently investigated as modulators of enzymes associated with carbohydrate digestion, cholinergic signalling, and melanogenesis.^13,14^ Scopoletin was tentatively detected exclusively in the ethanol extract, which also showed the strongest acetylcholinesterase inhibition. Since this coumarin has previously been associated with acetylcholinesterase-inhibitory and other biological effects,^60^ its co-occurrence with the strongest experimental activity provides a specific compound–target hypothesis for further investigation. It does not, however, establish scopoletin as the principal active constituent, because the activity of a crude extract may arise from additive, synergistic, or antagonistic interactions among several compounds.

The distribution of chlorophyll-derived compounds was less extract-specific than that of the very-long-chain fatty acids. Pheophorbide A was detected in the EA, ethanol, and ethanol/water extracts, whereas pheophytin A occurred in EA and ethanol. Neither was detected in the water extract. Their presence, together with the broader range of fatty acids detected in EA, further illustrates the preferential recovery of less-polar constituents by organic solvents, although their abundance cannot be inferred from the qualitative data.

Overall, the HPLC-ESI-Orbitrap-MS/MS results demonstrate pronounced solvent-dependent partitioning of the detectable phytochemical classes. The polar extracts provided broad coverage of hydroxycinnamic acids and flavonoid glycosides, whereas EA was distinguished by the exclusive detection of numerous long-chain saturated fatty acids and a broader representation of lipophilic constituents. The profiles identify several compounds that may contribute to the contrasting antioxidant and enzyme-inhibitory responses and therefore provide a rational basis for the subsequent molecular docking analysis. Nevertheless, definitive compound–activity relationships will require quantitative LC-MS determination, confirmation with authentic standards, bioactivity-guided fractionation, isolation of individual constituents, and direct experimental target validation.

### Antioxidant activity

Antioxidant activity varied considerably according to both extraction solvent and assay mechanism (Table 3). The water extract showed the highest numerical values in the DPPH (132.71 mg TE per g), CUPRAC (287.80 mg TE per g), and FRAP assays (251.99 mg TE per g), whereas the ethanol/water extract showed the highest numerical ABTS value (233.61 mg TE per g), although it did not differ significantly from the water extract. The ethanol extract ranked second in the DPPH assay (93.38 mg TE per g), while the EA extract produced the lowest responses in the four radical-scavenging and reducing-power assays. Metal-chelating activity did not differ significantly among the water (19.74 mg EDTAE per g), ethanol/water (18.56 mg EDTAE per g), and EA extracts (18.37 mg EDTAE per g), whereas the ethanol extract showed significantly lower activity (7.95 mg EDTAE per g). A different pattern was observed in the phosphomolybdenum assay, in which the EA extract gave the highest numerical value (2.99 mmol TE per g), followed by ethanol/water, water, and ethanol.

**Table 3:** Antioxidant activity^a^ of *Seseli libanotis* aerial-part extracts

Extracts	DPPH^b^ (mg TE per g)	ABTS^b^ (mg TE per g)	CUPRAC^b^ (mg TE per g)	FRAP^b^ (mg TE per g)	MC^c^ (mg EDTAE per g)	PBD^d^ (mmol TE per g)
EA	15.24 ± 0.49^d^	83.08 ± 6.22^c^	74.58 ± 1.66^d^	38.77 ± 0.99^d^	18.37 ± 0.36^a^	2.99 ± 0.11^a^
Ethanol	93.38 ± 1.17^b^	145.51 ± 2.87^b^	176.66 ± 2.32^c^	121.29 ± 3.65^c^	7.95 ± 0.59^b^	2.45 ± 0.06^c^
Ethanol/water	54.23 ± 7.64^c^	233.61 ± 11.73^a^	212.02 ± 19.21^b^	189.54 ± 4.36^b^	18.56 ± 0.90^a^	2.69 ± 0.08^b^
Water	132.71 ± 1.97^a^	211.26 ± 18.48^a^	287.80 ± 2.24^a^	251.99 ± 2.60^a^	19.74 ± 0.74^a^	2.66 ± 0.08^b,c^

a Values are presented as mean ± SD of three independent measurements (*n* = 3). Means within the same column followed by different superscript letters differ significantly at *p* ≤ 0.05.

b mg Trolox equivalents per g of extract.

c mg EDTAE per g of extract.

d mmol Trolox equivalents per g of extract.

The differences among the assays reflect the distinct reaction mechanisms through which antioxidant capacity is evaluated. DPPH and ABTS primarily measure radical-scavenging through hydrogen-atom and single-electron transfer pathways, whereas CUPRAC and FRAP assess the electron-donating or reducing capacity of the extracts. Metal-chelating activity represents a different antioxidant mechanism based on the sequestration of pro-oxidant transition metals, while the phosphomolybdenum assay reflects the overall capacity of chemically diverse constituents to reduce Mo(vi) to Mo(v) under acidic conditions.^61^ Consequently, variation in extract ranking among the assays is expected and indicates that no single method can provide a complete assessment of antioxidant potential.

The strong radical-scavenging and reducing-power responses of the water and ethanol/water extracts are broadly consistent with their high TPC values and the qualitative detection of caffeoylquinic acids, hydroxycinnamic acid derivatives, and flavonoid glycosides. Structural features such as the *ortho*-dihydroxy or catechol arrangement found in caffeoyl and quercetin-derived compounds favour electron donation and stabilisation of the resulting radicals and may therefore contribute to the observed antioxidant responses.^57,59,62^ However, direct compound–activity relationships cannot be established because the LC-MS analysis was qualitative and did not determine the concentrations of individual constituents.

The comparatively high phosphomolybdenum value of the EA extract, despite its lower TPC and weak performance in DPPH, ABTS, CUPRAC, and FRAP assays, suggests that non-phenolic or less-polar reducing constituents may also contribute to this assay. The EA extract contained a distinct range of fatty acids, oxylipins, coumarins, and chlorophyll-derived compounds, some of which may participate in the reduction of the phosphomolybdenum complex. Nevertheless, their individual contributions cannot be inferred from presence/absence LC-MS data and should be investigated through quantitative analysis and fractionation.

The DPPH results also did not strictly follow the TPC ranking, as the ethanol extract displayed stronger DPPH activity than the ethanol/water extract despite its lower TPC. This discrepancy is not unexpected because Folin–Ciocalteu values represent an overall reducing response rather than the concentration of compounds specifically active toward DPPH. Radical-scavenging activity depends on the concentration and structure of individual antioxidants, the accessibility and number of reactive hydroxyl groups, reaction kinetics, solvent conditions, and possible additive, synergistic, or antagonistic interactions within each extract. The results therefore reinforce the importance of evaluating plant extracts using complementary antioxidant methods rather than ranking them according to a single assay or total phenolic content alone.

Comparison with previous studies should be approached cautiously, even when equivalent assays and units are used, because antioxidant values are influenced by species and plant origin, analysed organ, extraction solvent and procedure, solid-to-solvent ratio, extract yield, and experimental conditions. Within these limitations, the values recorded in the present study were higher than those reported for *S. gummiferum* and *S. transcaucasicum* using the same assay panel and units: 41.67–53.20 mg TE per g for CUPRAC, 31.26–34.14 mg TE per g for FRAP, 5.51–11.45 mg TE per g for DPPH, and 43.46–51.91 mg TE per g for ABTS.^46^ Chlorogenic acid, which was tentatively annotated in all four extracts in the present study and previously reported as a major phenolic constituent of these related species, is a well-established dietary antioxidant and represents one plausible contributor to the observed responses.^46,57^ Its actual contribution, however, cannot be determined without quantitative LC-MS analysis and testing of isolated compounds.

### Enzyme inhibitory activity

The four extracts displayed clearly differentiated and target-dependent enzyme-inhibitory profiles (Table 4), indicating that extraction solvent influenced the pattern of biological activity. The ethanol and ethanol/water extracts showed the highest AChE inhibition, with values of 2.70 and 2.45 mg GALAE per g, respectively, and did not differ significantly from each other, whereas the EA extract showed the highest BChE inhibition (4.18 mg GALAE per g). BChE-inhibitory activity was not detected in the ethanol/water or water extracts. The ethanol/water extract exhibited the highest tyrosinase inhibition (54.32 mg KAE per g), followed by the ethanol extract (51.18 mg KAE per g). A contrasting pattern was observed for the carbohydrate-hydrolysing enzymes: EA showed the highest α-amylase inhibition (0.35 mmol ACAE per g), whereas ethanol and ethanol/water showed the highest α-glucosidase inhibition, with values of 1.68 and 1.54 mmol ACAE per g, respectively, and no significant difference between them.

**Table 4:** Enzyme inhibitory activity^a^ of *Seseli libanotis* aerial-part extracts^e^

Extracts	AChE^b^ (mg GALAE per g)	BChE^b^ (mg GALAE per g)	Tyrosinase^c^ (mg KAE per g)	α-Amylase^d^ (mmol ACAE per g)	α-Glucosidase^d^ (mmol ACAE per g)
EA	1.98 ± 0.31^b^	4.18 ± 0.46^a^	42.09 ± 1.63^c^	0.35 ± 0.01^a^	0.17 ± 0.10^c^
Ethanol	2.70 ± 0.03^a^	1.81 ± 0.19^b^	51.18 ± 0.70^b^	0.23 ± 0.01^b^	1.68 ± 0.14^a^
Ethanol/water	2.45 ± 0.04^a^	na	54.32 ± 0.41^a^	0.19 ± 0.01^c^	1.54 ± 0.13^a^
Water	0.15 ± 0.02^c^	na	38.75 ± 0.57^d^	0.09 ± 0.01^d^	0.48 ± 0.01^b^

a Values are presented as mean ± SD of three independent measurements (*n* = 3). Means within the same column followed by different superscript letters differ significantly at *p* ≤ 0.05.

b mg galantamine equivalents per g of extract.

c mg kojic acid equivalents per g of extract.

d mmol acarbose equivalents per g of extract.

e na – not active.

The cholinesterase results are particularly informative because AChE and BChE differ in active-site architecture and ligand selectivity. The stronger AChE inhibition of the ethanol and ethanol/water extracts may reflect the contribution of phenolic and coumarin-type constituents capable of forming hydrogen-bonding, π–π, and hydrophobic interactions within the enzyme gorge. Scopoletin was tentatively detected exclusively in the ethanol extract, which also exhibited the highest AChE inhibition. Given the reported cholinesterase-inhibitory potential of this coumarin, its occurrence provides a plausible chemical lead; however, the qualitative LC-MS data do not establish its concentration or individual contribution to the measured activity. The stronger BChE inhibition of EA may instead be related to its broader representation of less-polar constituents, including coumarins, oxylipins, fatty acids, and other lipophilic compounds. Because BChE possesses a larger and more permissive acyl-binding pocket than AChE, it may accommodate bulkier and more hydrophobic ligands more readily. Nevertheless, this interpretation remains provisional and requires validation using isolated compounds and kinetic studies.

The tyrosinase-inhibition pattern cannot be explained solely by the presence of flavonoid glycosides, since these compounds were widely detected in the ethanol, ethanol/water, and water extracts, while the water extract showed substantially weaker activity. This discrepancy suggests that inhibition depended not only on chemical class membership but also on the concentration, substitution pattern, glycosylation, accessibility, and combined action of individual constituents. Flavonoids and hydroxycinnamic acids may interact with tyrosinase through copper coordination, hydrogen bonding, or occupation of the catalytic pocket, but structural features such as the number and position of hydroxyl groups strongly influence their inhibitory behaviour.^13^ The higher activity of the ethanol/water and ethanol extracts may therefore reflect a more favourable combination of active constituents rather than the simple presence of a particular phytochemical class.

The opposing behaviour toward α-amylase and α-glucosidase is also noteworthy. The EA extract preferentially inhibited α-amylase, whereas the more polar ethanol and ethanol/water extracts were substantially more active against α-glucosidase. Flavonoids and phenolic acids are frequently reported to inhibit α-glucosidase through hydrogen bonding and hydrophobic interactions within or near the catalytic site, with activity strongly affected by hydroxylation and glycosylation patterns.^14^ The stronger α-glucosidase inhibition of the ethanol-based extracts is therefore chemically plausible, although it cannot be assigned to individual constituents without quantitative and compound-level testing. From a pharmacological perspective, stronger α-glucosidase inhibition accompanied by more moderate α-amylase inhibition is often considered a preferable profile for controlling postprandial glucose release, because excessive α-amylase inhibition may contribute to gastrointestinal adverse effects. The ethanol and ethanol/water extracts may therefore warrant further investigation in this context, although the present standard-equivalent values are insufficient to demonstrate therapeutic efficacy.

Comparison with related *Seseli* species provides only a broad frame of reference. Enzyme-inhibitory activities reported for *S. gummiferum* and *S. transcaucasicum* included α-amylase values of 0.12–0.78 mmol ACAE per g and cholinesterase activities of up to 9.71 mg GALAE per g, whereas their methanol extracts showed higher tyrosinase inhibition than observed here, reaching approximately 107–109 mg KAE per g.^46^ These differences may arise from species-specific phytochemistry, geographical origin, analysed plant material, extraction conditions, and assay calibration, and should therefore not be interpreted as direct evidence of greater or lower pharmacological potential.

The enzyme-inhibition data reveal a clear separation of activity according to target class: ethanol favoured AChE and α-glucosidase inhibition, EA favoured BChE and α-amylase inhibition, and ethanol/water was most effective against tyrosinase.

### Cytotoxicity and cell-death analysis

Cytotoxicity was evaluated against HeLa, DU-145, and A549 tumour cells and the HEK-293 reference cell line (Table 5). The ethanol/water extract showed the greatest cytotoxic effect, with the lowest IC_50_ values in all three tumour cell lines: 31.77 µg mL^−1^ in HeLa, 37.57 µg mL^−1^ in A549, and 41.25 µg mL^−1^ in DU-145 cells. The water and EA extracts displayed intermediate activity, whereas the ethanol extract was the least cytotoxic, with IC_50_ values above 117 µg mL^−1^ in the tumour cell lines.

**Table 5:** Cytotoxicity of *Seseli libanotis* aerial-part extracts against tumour and reference cell lines, expressed as IC_50_ values (µg mL^−1^)^a^

Extracts	HELA	DU-145	A549	HEK-293
EA	64.48 ± 2.14^b^	70.12 ± 2.53^c^	69.97 ± 2.36^b^	66.27 ± 2.11^b^
Ethanol	117.60 ± 3.85^c^	123.32 ± 4.27^d^	118.54 ± 3.68^c^	108.40 ± 3.41^c^
Ethanol/water	**31.77** ± 1.12^a^	41.25 ± 1.46^a^	37.57 ± 1.33^a^	35.20 ± 1.18^a^
Water	59.33 ± 2.08^b^	62.52 ± 2.34^b^	68.63 ± 2.47^b^	67.83 ± 2.21^b^

a Values are presented as mean ± SD of three independent measurements (*n* = 3). Means within the same column followed by different superscript letters differ significantly at *p* ≤ 0.05.

The cytotoxic activity was not accompanied by meaningful tumour-cell selectivity. The ethanol/water extract produced an IC_50_ of 35.20 µg mL^−1^ in HEK-293 cells, corresponding to selectivity indices of 1.11 for HeLa, 0.94 for A549, and 0.85 for DU-145 cells. More broadly, the selectivity indices calculated for all four extracts ranged from approximately 0.85 to 1.14, indicating that none preferentially affected the tumour cells under the applied conditions. The ethanol/water extract should therefore be described as broadly cytotoxic rather than selectively anticancer. Moreover, HEK-293 is an immortalised kidney-derived cell line and cannot fully represent the responses of normal primary tissues. Consequently, the present findings warrant further toxicological investigation but cannot be directly extrapolated to the dietary safety of *S. libanotis*.

Cytotoxic activity against HeLa cells has also been reported for the root essential oil of *S. libanotis*.^11^ However, direct comparison is not possible because that study examined a volatile root-derived fraction, used trypan-blue exclusion to assess cell viability, and expressed exposure in µL mL^−1^, whereas the present study evaluated non-volatile aerial-part extracts and determined IC_50_ values in µg mL^−1^. Nevertheless, both studies suggest that chemically distinct fractions of *S. libanotis* can reduce HeLa cell viability and warrant further compound-level and selectivity assessment.

**Fig. 2:**
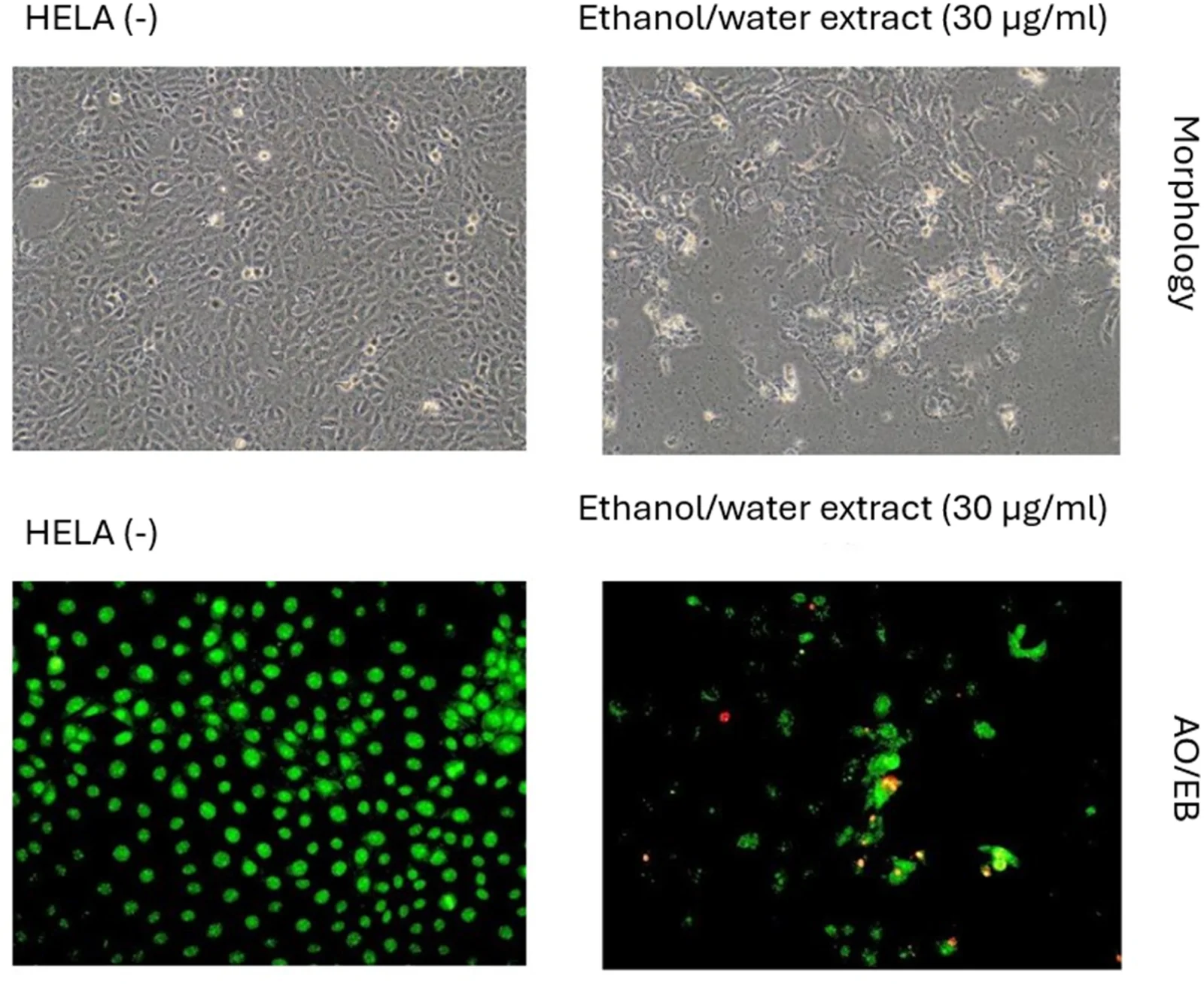
AO/EB staining after *Seseli libanotis* (ethanol/water) (30 µg mL^−1^) applied to HELA cell.

Since HeLa cells showed the lowest IC_50_ value, they were selected for further cell-death analysis using the ethanol/water extract at 30 µg mL^−1^. The AO/EB micrographs showed treatment-associated changes in cell density, morphology, and fluorescence, including an increase in orange- or red-fluorescent cells relative to the untreated control (Fig. 2). These observations are qualitatively consistent with cell-death-associated morphological changes and reduced membrane integrity. However, AO/EB imaging alone cannot quantify cell viability or establish a specific cell-death mechanism without systematic cell counting and complementary analyses.

**Fig. 3:**
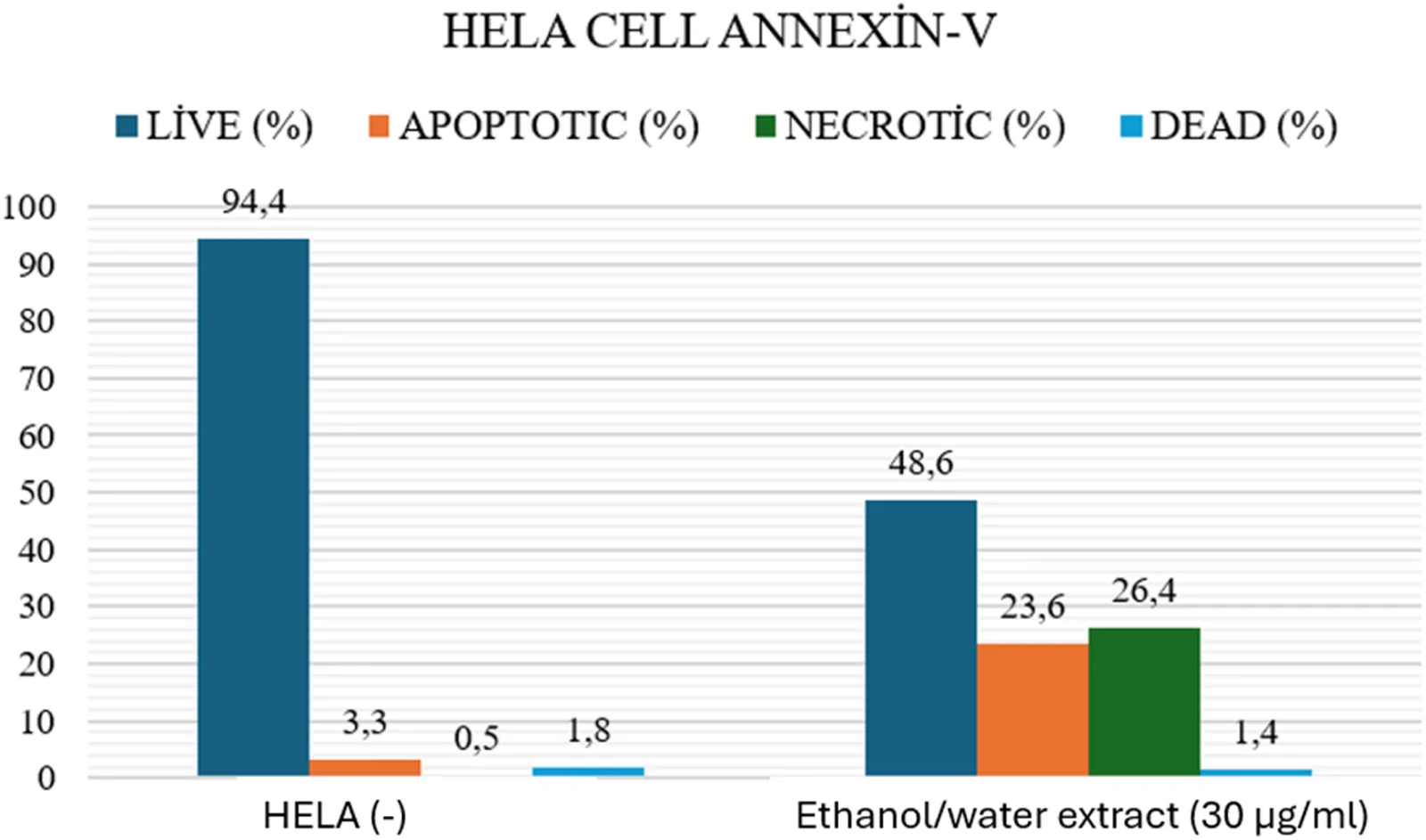
Annexin-V/PI staining results after applying *Seseli libanotis* (ethanol/water) (30 µg mL^−1^) to HeLa cells.

**Fig. 4:**
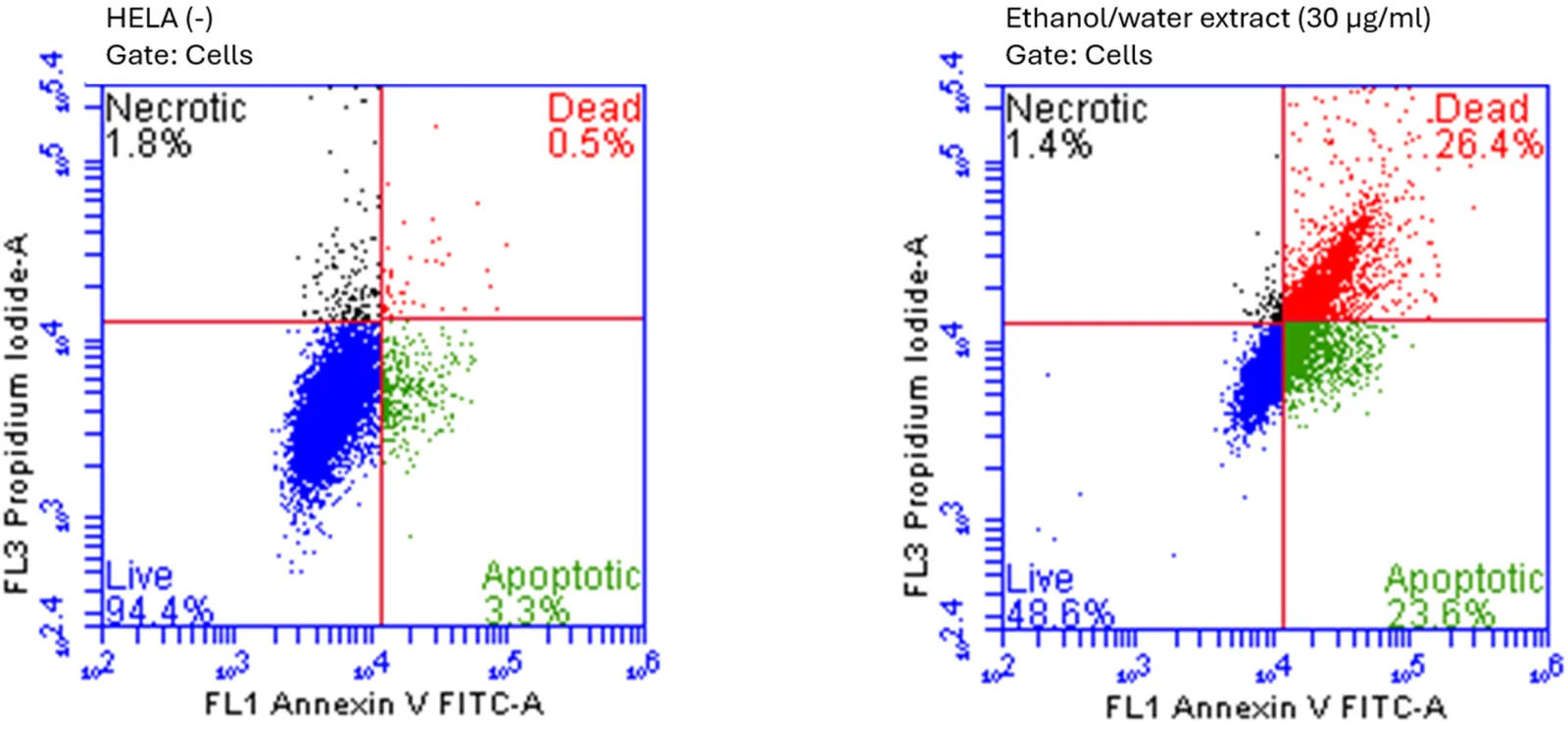
Annexin-V/PI staining results after *Seseli libanotis* (ethanol/water) (30 µg mL^−1^) to HELA cells. Blue: live-[(FITC^−^)/(PI^−^)]; green: early apoptotic [(FITC^+^)/(PI^−^)]; red: late apoptotic [(FITC^+^)/(PI^+^)]; black: shows necrotic [(FITC^+^)/(PI^+^)] cells.

Annexin V/PI analysis provided stronger evidence of treatment-associated redistribution among the cell populations. The viable Annexin V^−^/PI^−^ population decreased from 94.4% in the untreated control to 48.6% following treatment. Simultaneously, the early-apoptotic Annexin V^+^/PI^−^ fraction increased from 3.3% to 23.6%, while the Annexin V^+^/PI^+^ population increased from 0.5% to 26.4%. By contrast, the Annexin V^−^/PI^+^ population remained low, changing from 1.8% to 1.4%. Overall, the total Annexin V-positive population increased from 3.8% in the control to 50.0% after treatment. This pattern is consistent with an apoptosis-associated loss of viability rather than extensive primary necrosis (Fig. 3 and 4).

The Annexin V^+^/PI^+^ population should nevertheless be interpreted cautiously because this phenotype may represent late apoptotic cells, secondary necrosis, or a combination of both. Furthermore, the analysis was conducted at a single concentration. Confirmation of the cell-death pathway would therefore require concentration- and time-dependent experiments, independent biological replication, appropriate positive controls, and complementary molecular markers such as caspase activation, mitochondrial membrane depolarisation, or PARP cleavage.

The apoptosis-associated response is chemically plausible because some coumarins, caffeoylquinic acid derivatives, and flavonoids have demonstrated pro-apoptotic effects in other experimental systems.^2,62–64^ However, evidence obtained for individual representatives of these chemical classes cannot identify the constituents responsible for the activity of the crude ethanol/water extract. Specific compound–activity and mechanistic relationships therefore remain to be established through quantitative phytochemical analysis, bioactivity-guided fractionation, and testing of isolated compounds.

Taken together, the results show a clear solvent-dependent differentiation among the extracts. The water and ethanol/water extracts combined high total phenolic contents with generally strong radical-scavenging and reducing-power responses, while the ethanol/water extract additionally showed the highest tyrosinase inhibition and cytotoxicity. Ethanol was characterised by the highest total flavonoid content, whereas ethanol and ethanol/water jointly showed the highest AChE and α-glucosidase inhibition. In contrast, the EA extract displayed a distinct lipophilic profile together with the highest BChE, α-amylase, and phosphomolybdenum responses. These findings indicate that extraction polarity strongly influenced both the detected chemical profiles and biological activities, although the observed effects cannot be attributed to individual constituents.

The results should be interpreted cautiously because the LC-MS analysis was qualitative and the assays were performed using crude extracts. Enzyme-inhibition values represent comparative screening rather than mechanistic potency, while cytotoxic selectivity was assessed using only one non-cancerous reference cell line and cell-death analysis was conducted at a single concentration. Quantitative profiling, bioactivity-guided fractionation, testing of isolated compounds, additional non-cancerous cell models, and further mechanistic studies are therefore required.

### Network pharmacology analysis

**Fig. 5:**
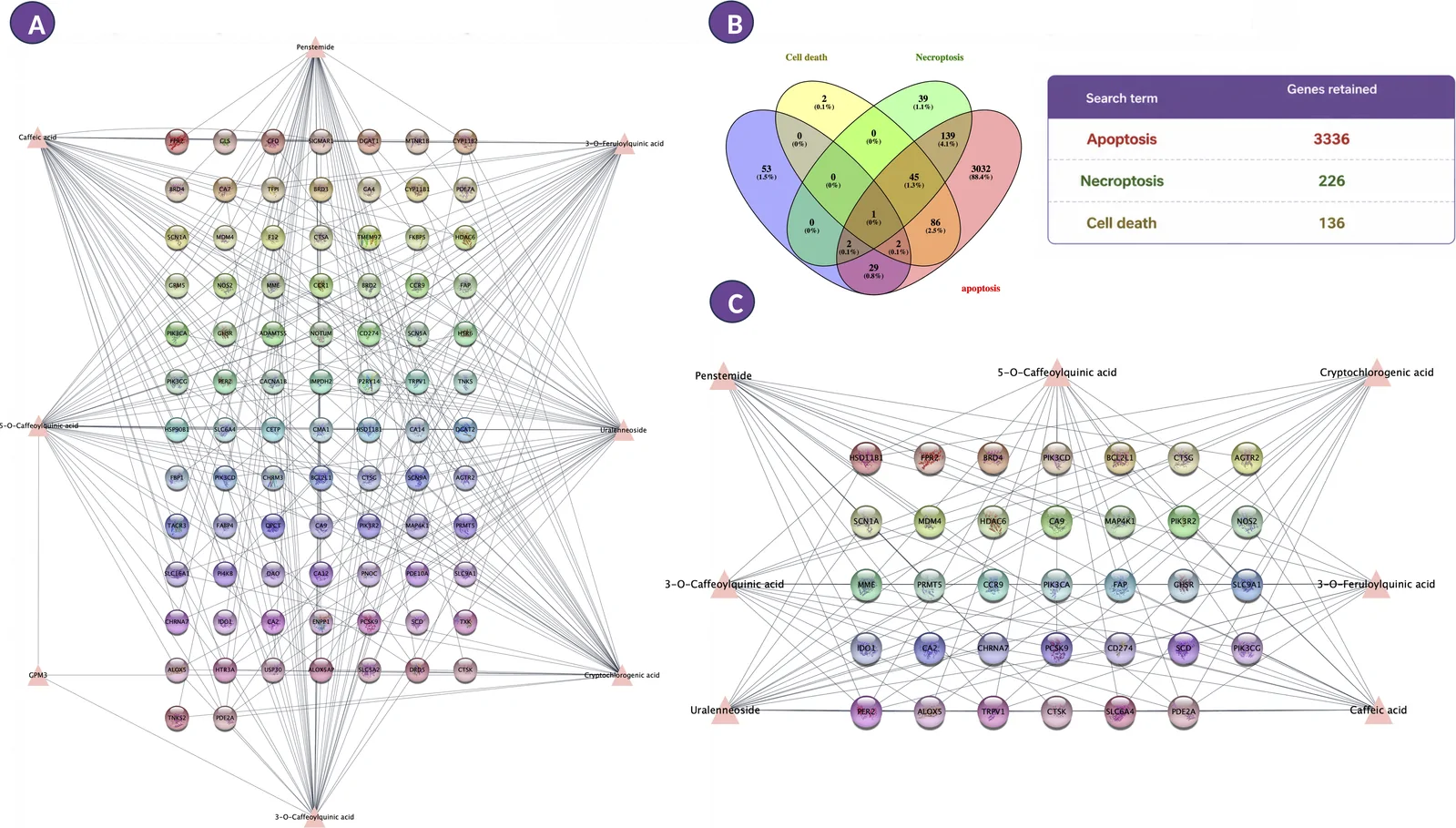
Compound–target network analysis. (A) Network of seven phytochemicals and 86 predicted targets. (B) Intersection with filtered regulated-cell-death gene sets, yielding 34 shared genes. (C) Ligand–target network of the 34 shared genes.

All selected phytochemicals were successfully processed using their canonical SMILES. Following ChEMBL-based target prediction, score filtering, restriction to human targets and GeneCards intersection, seven compounds contributed eligible associations to the final network: 5-*O*-caffeoylquinic acid, 3-*O*-caffeoylquinic acid, 3-*O*-feruloylquinic acid, cryptochlorogenic acid, caffeic acid, uralenneoside and penstemide. Disease-associated genes were retrieved independently from GeneCards using the search terms ‘‘apoptosis’’, ‘‘necroptosis’’, and ‘‘cell death”. Disease-associated genes were retrieved independently from GeneCards using the search terms “apoptosis”, “necroptosis”, and “cell death”. GeneCards relevance scores were rounded to the nearest whole number, and the retrieved genes were restricted to protein-coding genes with rounded relevance scores ≥15. Following these filtering criteria, 3.336 apoptosis-associated genes, 226 necroptosis-associated genes, and 136 cell-death-associated genes were retained (Fig. 5). The three term-specific gene lists were subsequently merged, and duplicate entries were removed to generate a unified regulated-cell-death gene set. Intersection of this unified gene set with the ChEMBL-predicted human targets identified 34 shared genes for downstream analysis. A total of 259 compound–target associations involving 86 unique human genes were retained before disease-specific filtering. Intersection with the GeneCards-derived regulated cell death gene set identified 34 shared targets. GeneCards relevance scores were rounded to the nearest whole number before application of the ≥15 threshold. The final disease-associated network contained 105 predicted compound–target associations, with prediction scores ranging from 0.591 to 0.955. Penstemide showed the broadest target coverage, with 18 shared targets, followed by uralenneoside with 17 and caffeic acid with 16. The caffeoylquinic acid derivatives and cryptochlorogenic acid were each associated with 14 targets, whereas 3-*O*-feruloylquinic acid was linked to 12 targets. Although caffeic acid showed relatively broad coverage, its mean prediction score was lower than those of the quinic acid derivatives. This indicates that target number and prediction confidence should be considered together rather than interpreted independently. Thirteen phytochemicals did not contribute eligible associations to the final network. Their SMILES were processed successfully, indicating that their absence was not due to structural input errors. Instead, they either did not produce predictions above the selected score threshold, lacked mapped human targets, or showed no overlap with the GeneCards-derived gene set. Therefore, their absence from the network should not be interpreted as biological inactivity, but rather as a lack of support within the current prediction and filtering framework.

### Compound–target and PPI network analysis

The final disease-filtered compound–target network contained seven phytochemicals, 34 shared human targets, and 105 predicted compound–target associations. Penstemide showed the broadest target coverage, with 18 shared targets, followed by uralenneoside with 17 and caffeic acid with 16. Neochlorogenic acid (5-*O*-caffeoylquinic acid), chlorogenic acid (3-*O*-caffeoylquinic acid), and cryptochlorogenic acid (4-*O*-caffeoylquinic acid) were each associated with 14 shared targets, whereas 3-*O*-feruloylquinic acid was associated with 12 targets. Several targets were recurrently predicted for multiple phytochemicals. *MME*, *PER2*, *TRPV1*, and *PDE2A* were associated with all seven network-forming compounds. *FAP*, *SLC9A1*, *HSD11B1*, and PIK3CD were associated with six compounds, whereas *CTSG*, *PIK3CG*, and *CD274* were associated with five. These results demonstrate substantial overlap among the predicted target profiles of the seven network-forming phytochemicals. However, the associations represent computational predictions and do not establish the direction or magnitude of compound activity against the corresponding proteins. Thirteen of the 20 evaluated phytochemicals did not contribute an eligible association to the final disease-filtered network. All compound structures were processed successfully; therefore, their exclusion was not attributable to structural-input failure. These compounds either produced no prediction above the score threshold of 0.50, lacked a mapped human gene target, or showed no intersection with the filtered GeneCards-derived regulated-cell-death gene set. Consequently, their absence from the final network should be interpreted as a lack of eligible association under the applied computational criteria rather than as evidence of biological inactivity (Fig. 5C).

### Transcriptomic validation of the network-derived targets

**Fig. 6:**
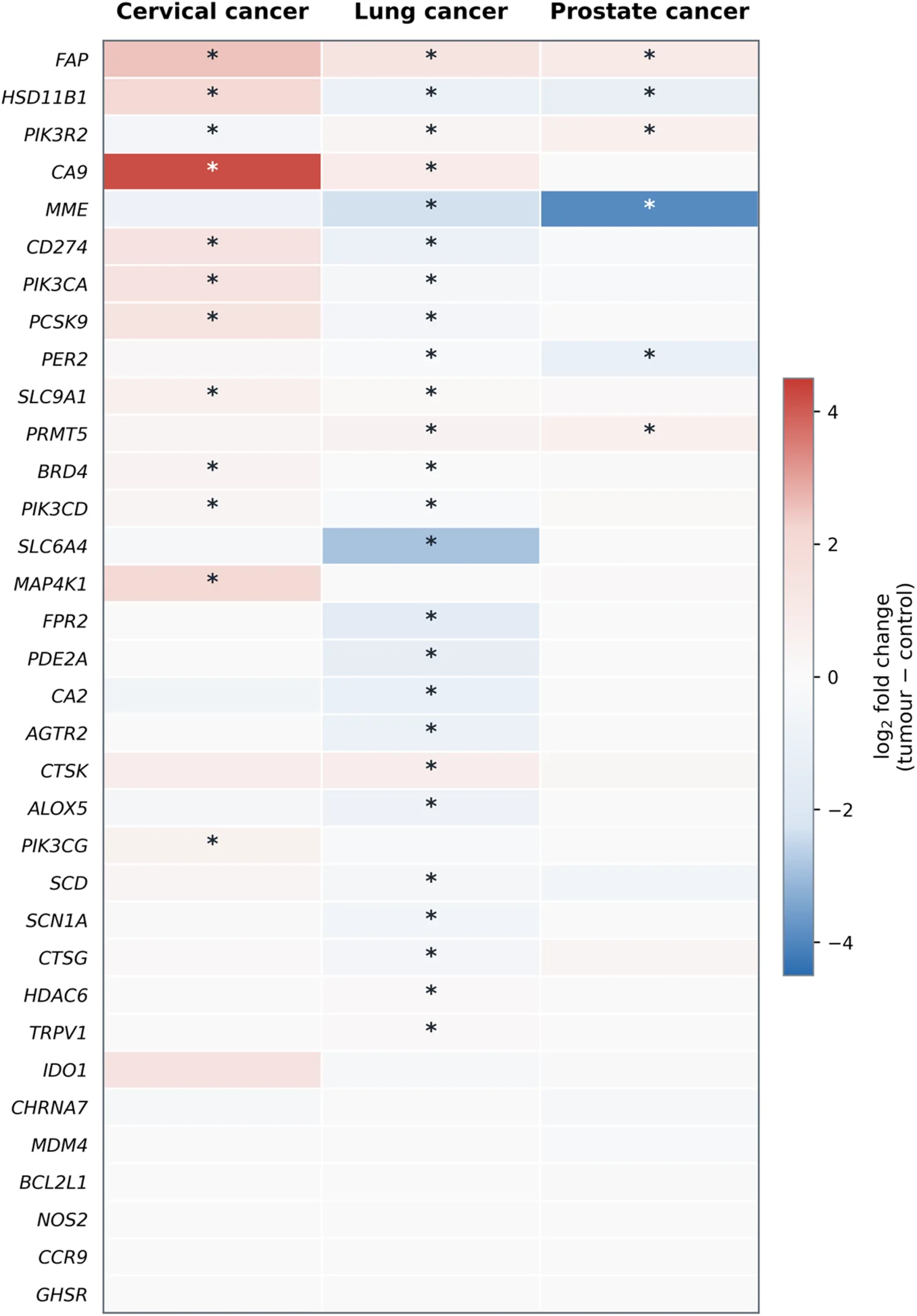
Cross-cancer differential-expression profiles of the predefined 34-gene signature. The heatmap shows limma-derived log_2_ fold-change values for tumor *versus* control comparisons in cervical cancer (GSE63514), lung cancer (GSE19804), and prostate cancer (GSE46602). Red indicates increased expression in tumor tissue, whereas blue indicates decreased expression. Asterisks denote genes that remained significant after Benjamini–Hochberg correction at FDR < 0.05 (*). Genes were ordered according to the number of cancer datasets in which they reached FDR significance and subsequently by their maximum absolute log_2_ fold change.

The 34 common targets identified through the network pharmacology analysis were subsequently evaluated using independent transcriptomic datasets representing cervical, lung, and prostate cancers. All 34 genes were successfully mapped in GSE63514, GSE19804, and GSE46602, allowing the same predefined signature to be examined across the three cancer types without dataset-specific gene replacement. Differential-expression analysis revealed cancer-specific differences in the transcriptional behaviour of the signature (Fig. 6 and Table S2). In the cervical cancer dataset, 12 of the 34 genes were significantly differentially expressed at FDR < 0.05, including 11 upregulated and one downregulated gene. The principal upregulated genes were *CA9*, *PIK3CA*, *HSD11B1*, *MAP4K1*, *SLC9A1*, *CD274*, *FAP*, and *BRD4*, whereas *PIK3R2* was downregulated. This expression pattern was consistent with the involvement of several interconnected processes, including PI3K signalling, cellular-stress adaptation, immune regulation, stromal activity, and transcriptional control. Among the three cancer types, the lung cancer dataset showed the most extensive transcriptional alteration of the 34-gene signature. The lung cancer dataset showed the most extensive transcriptional alteration. Twenty-five genes were significant at FDR < 0.05, of which eight were upregulated and 17 were downregulated. The major upregulated genes included *PIK3R2*, *FAP*, *PRMT5*, *CA9*, *HDAC6*, *CTSK*, *TRPV1*, and *SLC9A1*. In contrast, *MME*, *SLC6A4*, *PDE2A*, *FPR2*, *CD274*, *ALOX5*, *AGTR2*, and *SCN1A* were among the most prominent downregulated genes. The predominance of downregulated genes demonstrates that the signature does not represent a uniformly upregulated tumor-associated program. Rather, it may reflect broader reorganization across survival signalling, inflammation, metabolism, immune regulation, and tissue-remodelling processes. In the prostate cancer dataset, six of the 34 genes were significantly differentially expressed at FDR < 0.05. *PIK3R2*, *PRMT5*, and *FAP* were upregulated, whereas *MME*, *PER2*, and *HSD11B1* were downregulated. Compared with the cervical and lung datasets, the prostate dataset provided more limited transcriptional support for the complete 34-gene signature. This difference may reflect tumor-specific biology, cohort heterogeneity, or the smaller and less balanced composition of the prostate cohort, which comprised 36 tumor and 14 control samples. Collectively, the expression findings demonstrate that the network-derived targets are not altered uniformly across the three cancer types. The strongest transcriptomic support was obtained in lung cancer, followed by cervical cancer, whereas prostate cancer displayed a more restricted pattern. These findings indicate that the 34-gene set exhibits cancer-dependent transcriptional behavior rather than representing a uniformly dysregulated pan-cancer module (Fig. 6 and Table S2).

### Discriminative performance of gene signature

**Fig. 7:**
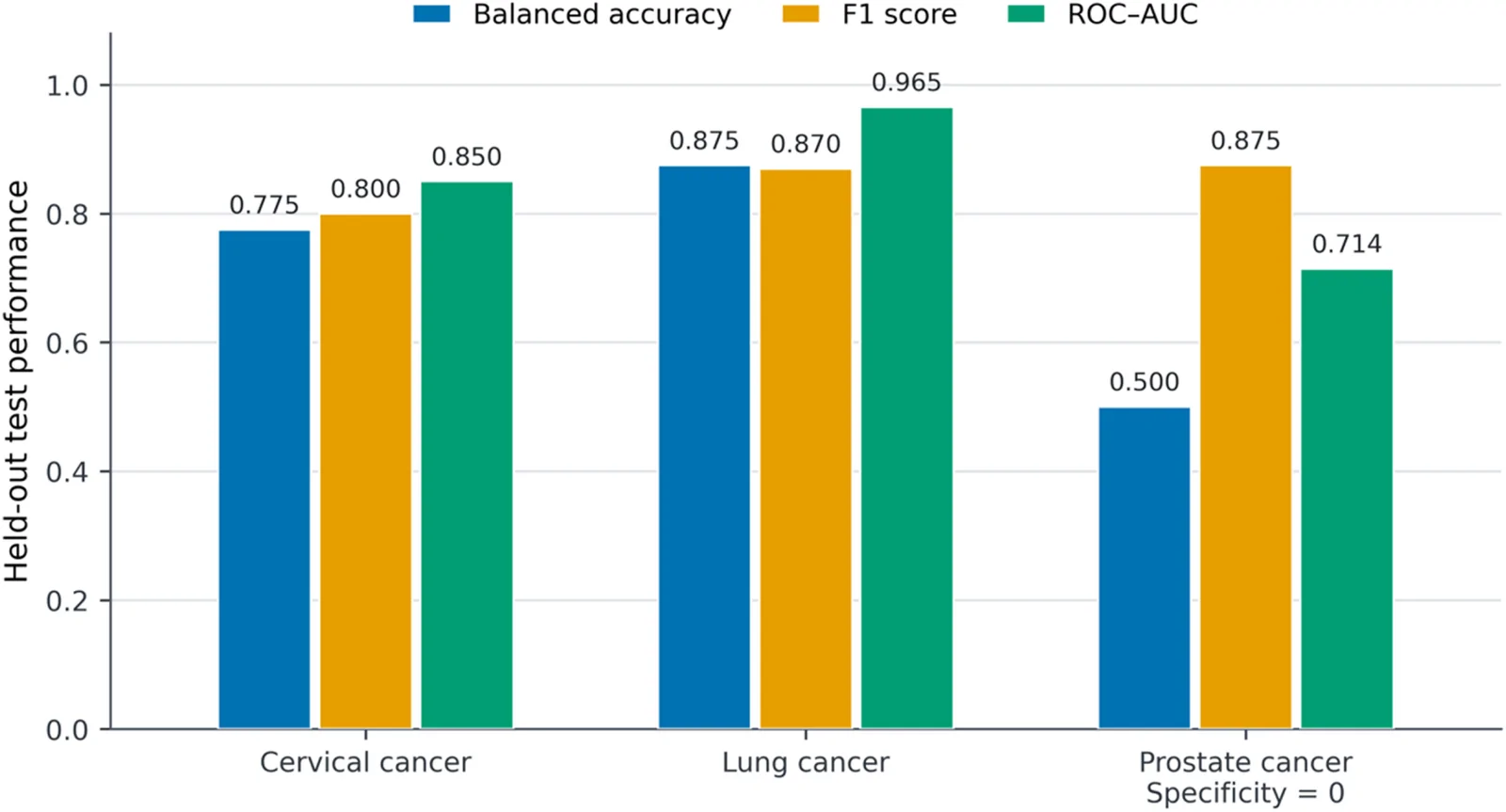
Held-out ElasticNet performance of the predefined 34-gene signature across three cancer datasets.

**Fig. 8:**
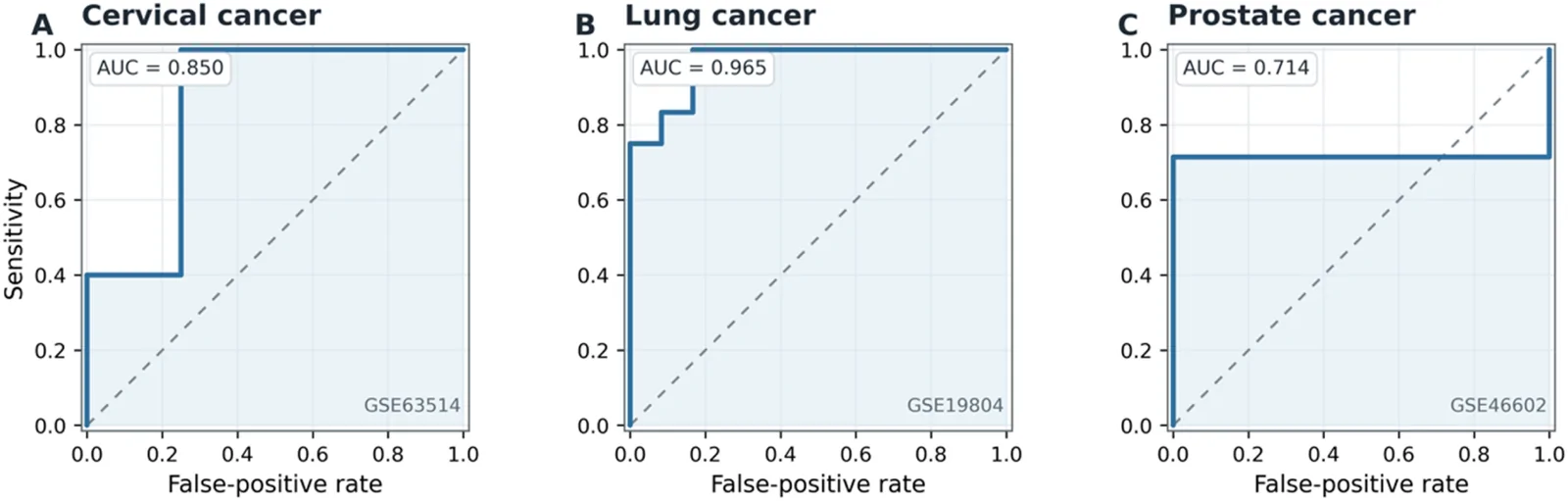
Receiver operating characteristic curves of the cancer-specific ElasticNet models based on the predefined 34-gene signature. ROC curves were generated from held-out test-set predictions for (A) cervical cancer (GSE63514), (B) lung cancer (GSE19804), and (C) prostate cancer (GSE46602).

ElasticNet logistic regression was used to assess whether the combined expression profile of the predefined 34 genes could distinguish tumor from control samples. For each dataset, model tuning was restricted to the training subset, and final performance was assessed using the held-out stratified test subset, comprising 9 cervical, 24 lung, and 9 prostate samples. In cervical cancer, the model achieved a test ROC–AUC of 0.850, an accuracy of 0.778, and a balanced accuracy of 0.775. Sensitivity and specificity were 0.800 and 0.750, respectively, while the *F*1 score was 0.800. The similar sensitivity and specificity values suggest that the model did not show an evident preference for either class within the small held-out test subset. Among the three datasets, the strongest held-out performance was observed for lung cancer. The model achieved a ROC–AUC of 0.965, an accuracy of 0.875, and a balanced accuracy of 0.875. Sensitivity was 0.833, specificity was 0.917, and the *F*1 score was 0.870. The agreement among these performance measures supports consistent tumor-control discrimination within the held-out lung cancer test subset. This result is also consistent with the extensive differential expression observed in the same dataset. The prostate cancer model yielded a ROC–AUC of 0.714 and an overall accuracy of 0.778. However, its balanced accuracy was only 0.500, with a sensitivity of 1.000 and a specificity of 0.000. Thus, the model correctly classified all seven tumor samples but failed to classify either of the two control samples in the held-out test subset. The apparently acceptable overall accuracy was therefore driven by the predominance of tumor samples in the test subset and should not be interpreted as evidence of reliable class-balanced discrimination. The contrast among the three cancer-specific models was analytically informative. The 34-gene signature showed promising held-out discrimination in lung cancer and moderate discrimination in cervical cancer but did not provide reliable class-balanced discrimination in the prostate cancer test subset. Accordingly, the signature should not be presented as a universal diagnostic classifier. Its principal value lies in providing supportive evidence that the network-derived targets contain cancer-specific transcriptional information, with the clearest held-out support observed in the lung cancer dataset (Table 6 and Fig. 7, 8).

**Table 6:** Held-out performance of ElasticNet logistic-regression models based on the predefined 34-gene signature^a^

Cancer type	GEO accession	Total *n* (tumour/control)	Training *n*	Test *n* (tumour/control)	ROC–AUC	Accuracy	Balanced accuracy	Sensitivity	Specificity	*F*1 score
Cervical cancer	GSE63514	52 (28/24)	43	9 (5/4)	0.850	0.778	0.775	0.800	0.750	0.800
Lung cancer	GSE19804	120 (60/60)	96	24 (12/12)	0.965	0.875	0.875	0.833	0.917	0.870
Prostate cancer	GSE46602	50 (36/14)	41	9 (7/2)	0.714	0.778	0.500	1.000	0.000	0.875

a Models were trained using the predefined 34-gene signature. Hyperparameter tuning was conducted exclusively within the training data using repeated cross-validation. Results represent a single stratified held-out test split and should be interpreted as internal exploratory validation. The prostate model showed no control-class discrimination.

### Cancer-specific model-associated genes

ElasticNet feature-importance profiles differed across the three cancer datasets. In cervical cancer, *PIK3CA* and *MAP4K1* were the two highest-ranked features, followed by *SLC9A1*, *CA2*, *CA9*, *BRD4*, *PIK3CD*, and *HSD11B1*. *PIK3CA*, *MAP4K1*, *SLC9A1*, *CA9*, *BRD4*, *PIK3CD*, and *HSD11B1* were also significantly differentially expressed, providing concordant support from the gene-level and multivariable analyses. The prominence of *PIK3CA*, *PIK3CD*, and *MAP4K1* is consistent with the network-level identification of signalling systems involved in cell survival, proliferation, immune-cell regulation, and cellular stress responses.^65,66^ The importance of CA9 and CA2 suggested a contribution from pH regulation and metabolic adaptation, while BRD4 connected the model-associated profile to transcriptional and epigenetic regulation. These findings indicate that feature importance reflected both tumor-associated differential expression and complementary discriminatory information not captured by gene-level significance alone. In lung cancer, *PDE2A* was the highest-ranked feature, followed by *FAP*, *CA2*, *MME*, *HSD11B1*, *CD274*, *SCD*, *CCR9*, *SCN1A*, and *IDO1*. Notably, several highly ranked genes, including *PDE2A*, *MME*, and *CD274*, were downregulated in tumor samples. This finding illustrates that machine-learning importance is not equivalent to tumor-associated upregulation. A downregulated gene may contribute strongly to classification when its expression pattern provides discriminatory information that is not captured by the remaining predictors. *FAP* ranked second in the lung cancer model and was significantly upregulated in tumor tissue, suggesting that stromal or tumor-microenvironment-associated variation may contribute to tumor-control discrimination. The relatively high importance of *CD274* and *IDO1* further suggested that immune-regulatory expression differences may contribute to the multigene classification pattern, although *IDO1* was not significantly differentially expressed. However, bulk transcriptomic data cannot determine whether these signals originate from tumor cells, stromal cells, or infiltrating immune populations. In prostate cancer, *MME* was the highest-ranked feature, followed by *PIK3R2*, *CCR9*, *HSD11B1*, *PER2*, *PRMT5*, *GHSR*, *CHRNA7*, and *FAP*. Nevertheless, these rankings should be interpreted cautiously because the final prostate model failed to classify control samples. The feature order describes the fitted model in the available cohort but does not establish a reproducible or externally validated prostate cancer signature. These findings emphasize that differential expression, network centrality, and machine-learning importance represent distinct forms of evidence and should not be treated as interchangeable measures (Table S3).

### Integrated interpretation and candidate prioritization

The transcriptomic and machine-learning analyses were used as additional evidence layers for the targets previously identified through network pharmacology. Machine learning was not employed as an independent target-discovery procedure; instead, it assessed whether the predefined 34-gene signature contained sufficient combined information to distinguish tumor from control samples. Targets considered for subsequent structural analysis were therefore selected using integrated evidence rather than ML rank alone. Candidate targets were prioritized according to their presence in the compound–target network, relevance to regulated cell death or tumor survival, significant tumor-associated expression changes in the GEO datasets, contribution to the multivariable model, availability of an experimentally determined protein structure, and presence of a biologically meaningful ligand-binding site. For cervical cancer, *PIK3CA*, *MAP4K1*, *CA9*, *HSD11B1*, and *BRD4* received comparatively consistent support. All five targets were significantly upregulated in the cervical cancer dataset and combined varying degrees of model contribution with biological and structural relevance to signalling, metabolic adaptation, or transcriptional regulation. In lung cancer, *FAP*, *PRMT5*, *HDAC6*, *CTSK*, and *CA9* were significantly upregulated and were further supported by their biological relevance and suitability for structure-based analysis. Although *PDE2A* had the highest ML importance in lung cancer, its significant downregulation meant that it was not automatically prioritised as an inhibitory docking target in the absence of a clear mechanistic rationale. In prostate cancer, *PRMT5* and *FAP* were retained as structurally suitable candidates because both were significantly upregulated and contributed to the fitted ElasticNet model. However, because the prostate classifier lacked specificity, these targets were interpreted as expression- and structure-supported candidates rather than as products of a successfully validated predictive model. Taken together, the integrated analysis provided the strongest support for target prioritisation in lung cancer, followed by cervical cancer. The results also showed that no single analytical layer was sufficient on its own. Network pharmacology predicted compound–target relationships, GEO analysis evaluated tumor-associated expression, and ElasticNet quantified the multivariable discriminatory contributions of genes within the predefined signature. Candidate selection was therefore considered most defensible when these complementary lines of evidence converged (Table S4).

### Ligand binding energy and interaction

The molecular docking analysis identified several phytochemicals with strong binding and favorable interaction profiles across the investigated targets (Fig. S5 and S6). For instance, quercetin 3-*O*-rhamnoside exhibited the strongest binding against BChE (−10.3 kcal mol^−1^), PRMT5 (−10.8 kcal mol^−1^), MAP4K1 (−9.5 kcal mol^−1^), and BRD4 (−9.2 kcal mol^−1^). These complexes were predominantly stabilized by extensive hydrogen bonding, hydrophobic contacts, π-interactions, salt bridges, and metal coordination, supporting the multitarget therapeutic potential of these phytochemicals.

**Fig. 9:**
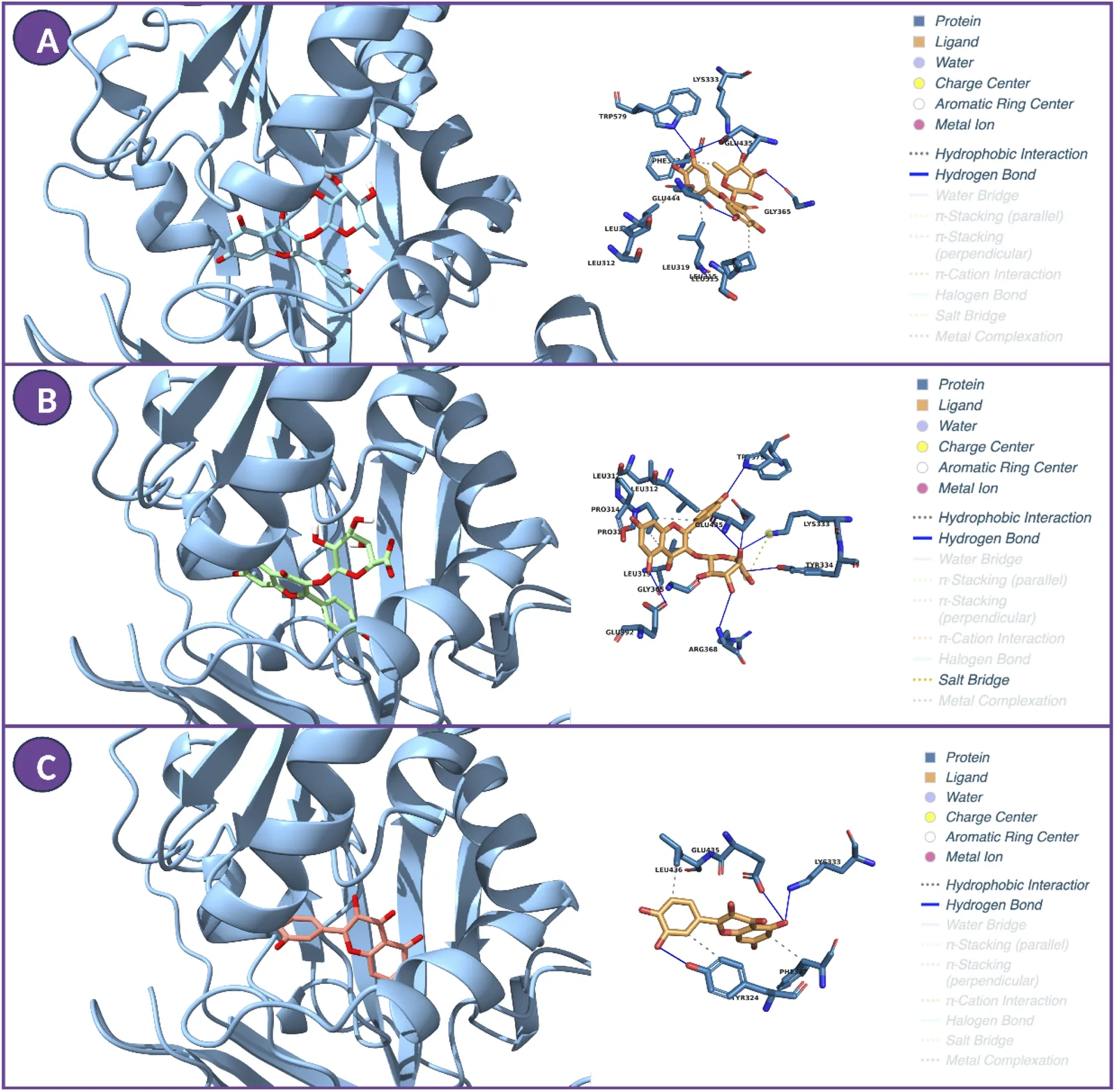
Molecular docking poses and protein–ligand interaction profiles of the top three phytochemicals within the binding pocket of the target protein. (A) Quercetin 3-*O*-rhamnoside, (B) kaempferol 3-*O*-glucuronide, and (C) quercetin.

Quercetin 3-*O*-rhamnoside (Fig. 9A) and kaempferol 3-*O*-glucuronide (Fig. 9B) formed hydrogen bonds with Lys333, Glu435, Glu444, Gly365, and Tyr334, supported by hydrophobic contacts with Leu312, Leu319, Leu315, and Phe327. This agrees with the pharmacophore of validated PRMT5 inhibitors at this pocket, where Glu435/Glu444 consistently anchor ligand polar groups.^67^ Engagement of this glutamate pair is mechanistically significant, as it coordinates the substrate arginine guanidinium during catalysis in the native PRMT5:MEP50–peptide complex,^68^ suggesting the docked poses recapitulate a catalytically relevant interaction rather than a peripheral contact.^69^

Kaempferol 3-*O*-glucuronide additionally formed a salt bridge with Lys333 *via* its glucuronide moiety. Lys333 has independently been identified as a high-frequency contact and hydrogen-bond partner in MD simulations of PRMT5 inhibitors at this site,^70^ implying that glycosylation may confer extra electrostatic stabilization relative to the aglycone. Quercetin itself engaged a more limited network; Lys333, Glu435, and Tyr324, with hydrophobic contacts to Leu436 and Phe327 (Fig. 9C), consistent with its smaller size and fewer hydrogen-bond donors/acceptors relative to its glycosylated counterparts, in line with SAR trends showing that peripheral substituents extending toward the solvent-exposed hydrophobic subpocket (Tyr304, Phe327, Ser578, Phe580) improve pose stability.^71^

These docking results support the plausibility of flavonol glycosides and their aglycone as substrate-competitive PRMT5 binders that recapitulate key interactions of established inhibitors, particularly engagement of the catalytic Glu435/Glu444 dyad. The comparatively richer interaction network of the glycosylated ligands, notably the ionic contact from kaempferol 3-*O*-glucuronide, suggests glycosylation may enhance predicted binding stability.

### Ligand binding mode stability

#### Conformational behavior and ligand stability

**Fig. 10:**
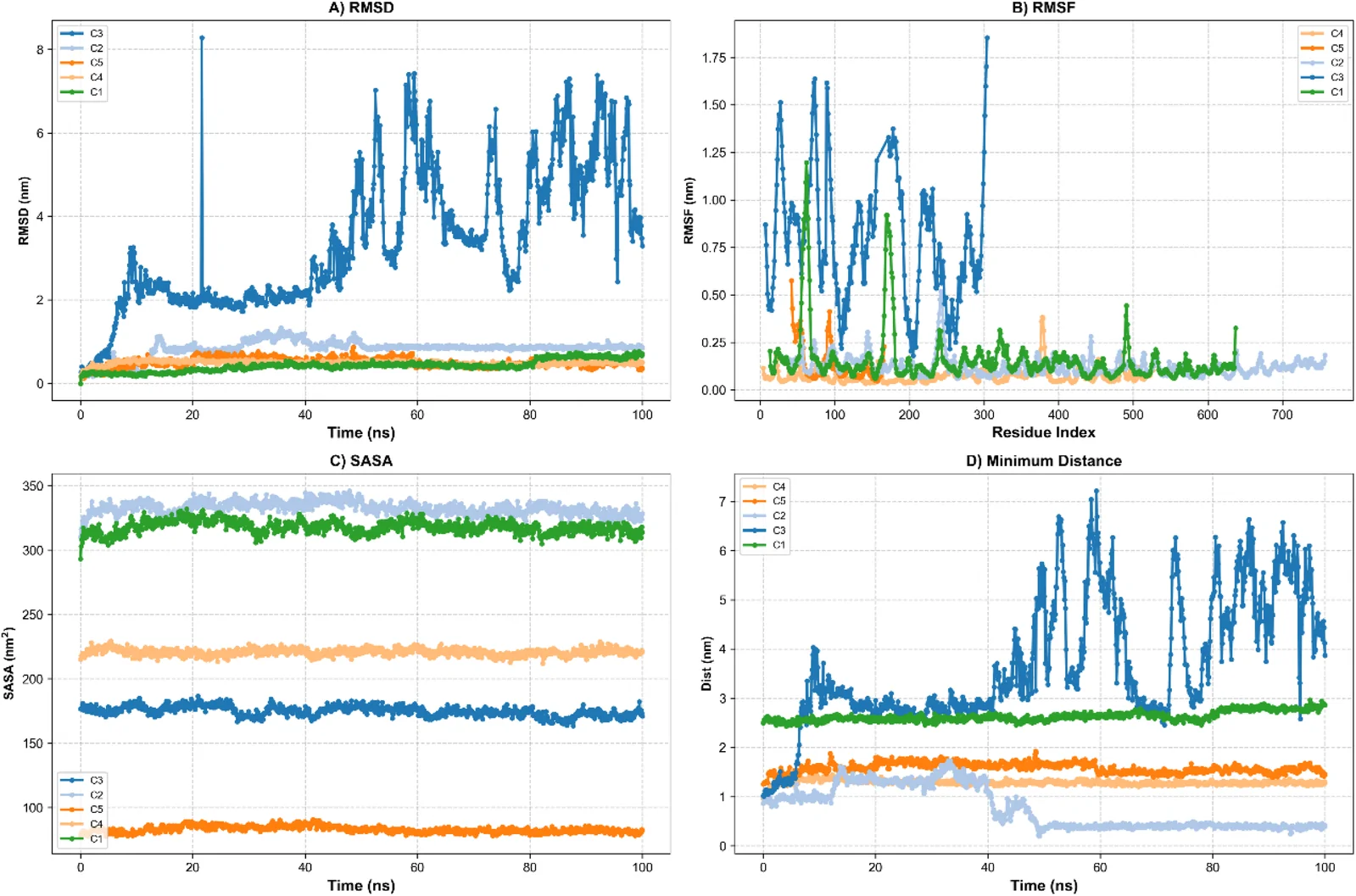
Dynamic profiles of selected docking complexes over 100 ns: (A) RMSD, (B) RMSF, (C) SASA, and (D) protein–ligand distance. (A) PRMT5–kaempferol 3-*O*-glucuronide (C1), (B) MAP4K1–nicotiflorin (C3), (C) FAP–quercetin 3-*O*-rhamnoside (C2), (D) BRD4–quercetin 3-*O*-rhamnoside (C5), (E) BChE–quercetin 3-*O*-rhamnoside (C4).

Four of the five complexes behave as expected for well-docked, stable poses: C1 (PRMT5–kaempferol glucuronide), C4 (BChE–rhamnoside) and C5 (BRD4–rhamnoside) equilibrate within the first ∼10 ns and hold steady between roughly 0.4–0.9 nm for the full 100 ns, while C2 (FAP–rhamnoside) settles at a slightly higher but still stable plateau near 0.8–1.0 nm. C3 (MAP4K1–nicotiflorin) is the clear outlier; it exceeds 2 nm almost immediately, spikes past 8 nm around 21 ns, and after ∼40 ns enters a regime of large, repeated 3–7 nm excursions that never re-stabilize through 100 ns (Fig. 10A). This pattern is textbook evidence of a non-equilibrated, poorly bound complex rather than a genuine alternative low-energy conformation; sustained multi-nanometer RMSD swings after an initial plateau typically indicate ligand displacement or partial unfolding rather than simple thermal noise.^72^

### Residual fluctuation

Per-residue flexibility is broadly consistent with each protein's own fold: most systems share a common low-fluctuation baseline (<0.2 nm) across their more rigid, well-packed regions, with characteristic peaks at surface loops (Fig. 10B). C4 is the most rigid overall. C1 shows two sharp, high peaks (∼1.2 nm near residue 60–90 and ∼0.9 nm near residue 170) that correspond to flexible surface loops rather than the core fold. C5 and C2 show comparatively modest, localized peaks. C3 again displays large fluctuations (1.0–1.85 nm) spanning almost the entire modeled sequence rather than one or two loops, and this is consistent with the global instability already seen in its RMSD trace, not a local effect, supporting the preservation of overall structural integrity during ligand binding.^73^

### Surface area accessibility

The five traces separate cleanly into stable bands that track target identity rather than ligand behavior (Fig. 10C): C2 has the highest SASA (∼320–345 nm^2^), followed closely by C1 (∼300–330 nm^2^), C4 (∼210–230 nm^2^), C3 (MAP4K1), (∼165–185 nm^2^), and C5, which is the lowest by far (∼65–90 nm^2^). This trend is consistent with BRD4's bromodomain being a much smaller single-domain fold than the larger, multidomain FAP or PRMT5. Within each system, the SASA trace is essentially flat for the full 100 ns. Also, the C3 instability is more likely a conformational rearrangement or ligand excursion within an intact fold than global denaturation.^74^

### Protein–ligand distance

C4 and C5 consistently exhibit short distances (∼1.3 nm and ∼1.5–1.9 nm, respectively). C1 is flat around 2.5–2.9 nm throughout, a stable but comparatively distant relationship. C2 is the most interesting: it starts around 1 nm, drifts up to ∼1.5–1.8 nm through 30 ns, then drops sharply after ∼40 ns to a tight ∼0.4–0.5 nm and stays there, and this indicates that the ligand settles into a closer, more committed pose partway through the run. C3 again shows an early rise, then oscillates remarkably between ∼2.5 and 7+ nm from ∼45 ns onward (Fig. 9D), which at these magnitudes is consistent with the ligand repeatedly leaving the binding pocket into bulk solvent rather than maintaining a stable contact.^75^

### Hydrogen bond dynamics

**Fig. 11:**
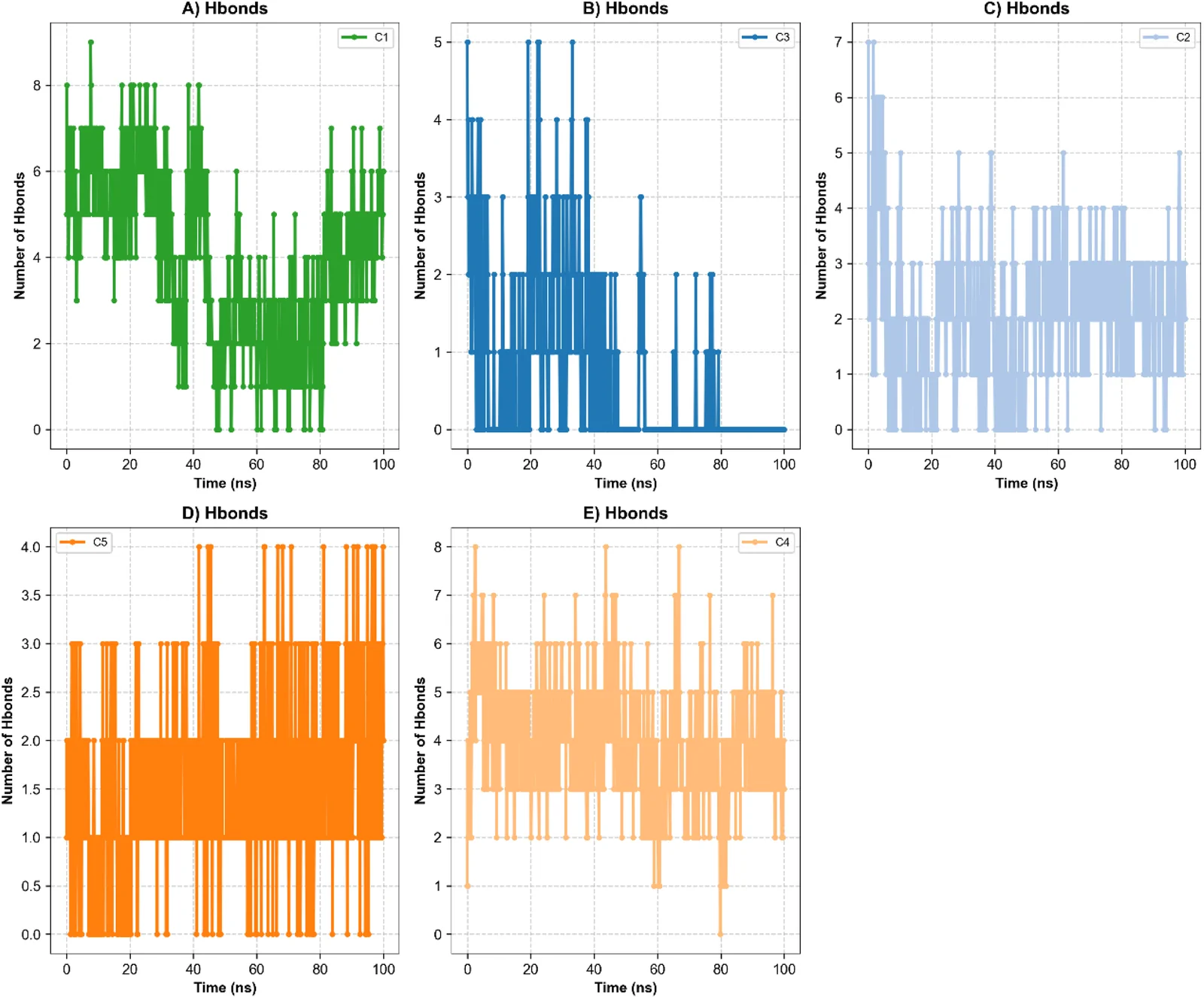
Time evolution of protein–ligand hydrogen bonds during 100 ns MD simulation: (A) PRMT5–kaempferol 3-*O*-glucuronide (C1), (B) MAP4K1–nicotiflorin (C3), (C) FAP–quercetin 3-*O*-rhamnoside (C2), (D) BRD4–quercetin 3-*O*-rhamnoside (C5), (E) BChE–quercetin 3-*O*-rhamnoside (C4).

For C1, H-bond count is high and consistently maintained, fluctuating mostly in the ∼4–8 range across the full trajectory with no sustained collapse to zero (Fig. 11A). This is the richest and most persistent H-bonding profile of the five complexes, indicating the glucuronide moiety is engaging in multiple, largely stable polar contacts with the PRMT5 pocket throughout the simulation.

Conversely, C3 has the weakest H-bond profile: counts oscillate mostly between 1 and 5, with numerous intervals where the count appears to drop out entirely (no bond detected for stretches of the trajectory) (Fig. 11B). This pattern agrees directly with the large increase in RMSD after ∼40 ns and the minimum-distance spikes to 4–7 nm. Loss of hydrogen bonding coinciding with increased ligand–pocket separation and backbone RMSD/RMSF is a classic signature of a ligand losing its binding pose rather than merely repositioning within it, since backbone/ligand hydrogen bonds are what hold a pose in its energy minimum in the first place.^72^

In the case of C2, the H-bond count is moderate-to-high and the broadest-ranging of the stable complexes, spanning roughly 1–7 over the run with a fairly high average (∼4) (Fig. 11C). Interestingly, this tracks with the minimum-distance behavior noted for C2, where the pocket-tightening event after ∼40 ns is consistent with an increase in H-bond engagement. The ligand appears to settle into a more extensively hydrogen-bonded pose partway through the simulation rather than losing contacts.^76^

For C5, H-bond count is low but tightly constrained, staying mostly within a narrow 1–4 band for the entire run (Fig. 11D). A narrow, low-but-nonzero H-bond count is typical of a small, single-domain binding pocket like a bromodomain, where only one or two canonical contacts are geometrically available regardless of ligand identity.^77^

Finally, the C4 exhibits a high H-bond count and is the most consistently sustained of all five complexes, staying mostly at 4–8 with a minimum that rarely falls below ∼4 (Fig. 11E). This behavior is also in agreement with the flattest RMSD/RMSF trends, suggesting the most stable binding pose among the five systems.^78^ Therefore, the H-bond profile reinforces the stability ranking already suggested by the structural metrics that C4 and C1 combine low structural deviation with high, sustained H-bond counts, suggesting durable, well-anchored complexes.

## Conclusion

This study expands the phytochemical and biological characterisation of *S. libanotis* aerial parts through a solvent-resolved comparison of four extracts across complementary chemical, antioxidant, enzyme-inhibitory, computational, and cell-based analyses. Under the applied conditions, extraction polarity was a major determinant of both the detected chemical profiles and biological responses. Water-containing extracts showed the highest total phenolic contents and generally strong radical-scavenging and reducing-power responses. Ethanol yielded the highest total flavonoid content, while ethanol and ethanol/water showed the highest AChE and α-glucosidase inhibition; in contrast, the EA extract displayed a distinct lipophilic profile together with the highest BChE, α-amylase, and phosphomolybdenum responses. The broad correspondence between the qualitative HPLC-ESI-Orbitrap-MS/MS profiles and the biological results provides a coherent basis for further compound-level investigation. The ethanol/water extract showed the greatest cytotoxicity and produced Annexin V/PI changes consistent with an apoptosis-associated loss of viability. However, its comparable activity against HEK-293 cells indicated limited tumour-cell selectivity, precluding direct anticancer claims and warranting further safety evaluation. Quantitative LC-MS analysis, confirmation with authentic standards, bioactivity-guided fractionation, enzyme-kinetic studies, testing of isolated compounds, and evaluation in additional non-cancerous and *in vivo* models are therefore required. The present study establishes a chemical and biological framework for identifying the constituents responsible for the target-dependent activities of *S. libanotis*.

## Conflicts of interest

There are no conflicts to declare.

## Supplementary Material

RA-016-D6RA07396D-s001

## Data Availability

The data supporting this article have been included as part of the supplementary information (SI). Supplementary information: Table S1: proposed docking grid centre coordinates and box dimensions for selected protein targets (docking pending). Table S2: differential expression of the predefined 34-gene signature in cervical, lung, and prostate cancer GEO datasets. Table S3: non-zero relative feature importance values from the fitted ElasticNet models based on the predefined 34-gene signature. Table S4: integrated prioritisation of targets for future molecular docking analysis. Table S5: molecular docking scores, RMSD values, and protein–ligand interaction profiles of the investigated compounds against the standard enzyme targets. Table S6: molecular docking scores cancer target protein–ligand interaction profiles of the investigated compounds against the selected targets. See DOI: https://doi.org/10.1039/d6ra07396d.
