# Supplementary material for: Extraction solvent shapes the phytochemistry, multi-target bioactivity, and machine learning-predicted anticancer targets of Seseli libanotis
Source: RSC Adv. 2026 Aug 28;16(42):51134–56. doi: 10.1039/d6ra07396d (PMC13523778; doi:10.1039/d6ra07396d)
Supplement: Supplementary file 1 [file RA-016-D6RA07396D-s001.pdf]

Supplementary Information (SI) for RSC Advances.
This journal is © The Royal Society of Chemistry 2026

Table S1. Proposed docking grid centre coordinates and box dimensions for selected protein targets (docking pending).

Target protein	PDB ID	Centre X, Y, Z (Å)	Grid box X, Y, Z
AChE	7E3H	-54.44, 32.90, -28.65	25, 25, 25
BChE	6EQP	32.16, -16.33, 40.73	25, 25, 25
α-Glucosidase	7KBJ	-18.79, 2.18, 15.75	25, 25, 25
BRD4	4QR3	6.907, -3.103, 7.407	40, 40, 54
CA9	5FL6	-22.032, -5.698, 0.231	126, 126, 126
CTSK	5Z5O	44.948, 55.894, 50.093	126, 126, 126
FAP	1Z68	38.034, 0.530, 69.455	126, 126, 124
HDAC6	5EDU	17.210, -44.560, 101.810	16, 24, 19
HSD11B1	3FCO	29.419, 48.142, 83.231	126, 126, 124
MAP4K1	7KAC	23.013, -77.484, -5.321	50, 66, 88
PIK3CA	2V1Y	68.512, 50.026, -16.585	126, 126, 126
PRMT5	5EML	20.468, -59.921, -15.501	40, 126, 96

Table S2. Differential expression of the predefined 34-gene signature in cervical, lung, and prostate cancer GEO datasets.

Cancer type	GEO accession	Gene	log2FC (tumour vs control)	FDR	Direction	FDR < 0.05
Cervical cancer	GSE63514	BCL2L1	0.00685629	0.930254	Upregulated in tumor	No
Cervical cancer	GSE63514	NOS2	-0.0343267	0.526974	Downregulated in tumor	No
Cervical cancer	GSE63514	ALOX5	-0.321754	0.0935466	Downregulated in tumor	No
Cervical cancer	GSE63514	PIK3CA	1.47995	1.05e-06	Upregulated in tumor	Yes
Cervical cancer	GSE63514	CD274	1.49071	0.000697997	Upregulated in tumor	Yes
Cervical cancer	GSE63514	PCSK9	1.41426	0.0169424	Upregulated in tumor	Yes
Cervical cancer	GSE63514	MDM4	-0.0131182	0.956852	Downregulated in tumor	No
Cervical cancer	GSE63514	CTSG	0.175673	0.644814	Upregulated in tumor	No
Cervical cancer	GSE63514	FAP	2.50864	0.0013941	Upregulated in tumor	Yes
Cervical cancer	GSE63514	PIK3CD	0.378318	0.0493055	Upregulated in tumor	Yes
Cervical cancer	GSE63514	MAP4K1	2.01828	0.000381612	Upregulated in tumor	Yes
Cervical cancer	GSE63514	CCR9	0.00245996	0.909851	Upregulated in tumor	No
Cervical cancer	GSE63514	SCD	0.349729	0.367603	Upregulated in tumor	No
Cervical cancer	GSE63514	IDO1	1.46201	0.12369	Upregulated in tumor	No
Cervical cancer	GSE63514	SCN1A	0.10248	0.633036	Upregulated in tumor	No
Cervical cancer	GSE63514	HSD11B1	2.01127	0.000221065	Upregulated in tumor	Yes
Cervical cancer	GSE63514	PDE2A	-0.0469069	0.897606	Downregulated in tumor	No
Cervical cancer	GSE63514	PIK3R2	-0.455485	0.0031113	Downregulated in tumor	Yes
Cervical cancer	GSE63514	TRPV1	-0.072698	0.236704	Downregulated in tumor	No
Cervical cancer	GSE63514	FPR2	-0.0453097	0.133778	Downregulated in tumor	No
Cervical cancer	GSE63514	SLC9A1	0.61492	0.000496876	Upregulated in tumor	Yes
Cervical cancer	GSE63514	PIK3CG	0.540571	0.0311435	Upregulated in tumor	Yes
Cervical cancer	GSE63514	GHSR	-0.0283271	0.231557	Downregulated in tumor	No
Cervical cancer	GSE63514	CHRNA7	-0.227695	0.561692	Downregulated in tumor	No
Cervical cancer	GSE63514	MME	-0.748178	0.208322	Downregulated in tumor	No
Cervical cancer	GSE63514	AGTR2	-0.0597407	0.296438	Downregulated in tumor	No
Cervical cancer	GSE63514	PER2	0.178173	0.35848	Upregulated in tumor	No
Cervical cancer	GSE63514	BRD4	0.400823	0.00203304	Upregulated in tumor	Yes
Cervical cancer	GSE63514	HDAC6	-0.0242082	0.846418	Downregulated in tumor	No
Cervical cancer	GSE63514	PRMT5	0.319214	0.367318	Upregulated in tumor	No
Cervical cancer	GSE63514	CTSK	0.811681	0.246448	Upregulated in tumor	No
Cervical cancer	GSE63514	CA9	4.19221	2.61e-08	Upregulated in tumor	Yes
Cervical cancer	GSE63514	SLC6A4	-0.27181	0.624693	Downregulated in tumor	No
Cervical cancer	GSE63514	CA2	-0.552216	0.608037	Downregulated in tumor	No
Lung cancer	GSE19804	BCL2L1	-0.0858731	0.518787	Downregulated in tumor	No
Lung cancer	GSE19804	NOS2	-0.0830982	0.0762581	Downregulated in tumor	No
Lung cancer	GSE19804	ALOX5	-0.739279	4.59e-11	Downregulated in tumor	Yes
Lung cancer	GSE19804	PIK3CA	-0.363357	0.000828022	Downregulated in tumor	Yes
Lung cancer	GSE19804	CD274	-1.00629	3.74e-11	Downregulated in tumor	Yes
Lung cancer	GSE19804	PCSK9	-0.442049	3.24e-06	Downregulated in tumor	Yes
Lung cancer	GSE19804	MDM4	-0.0721676	0.369579	Downregulated in tumor	No
Lung cancer	GSE19804	CTSG	-0.424816	0.00714388	Downregulated in tumor	Yes
Lung cancer	GSE19804	FAP	1.35783	3.85e-10	Upregulated in tumor	Yes
Lung cancer	GSE19804	PIK3CD	-0.205964	0.00539394	Downregulated in tumor	Yes
Lung cancer	GSE19804	MAP4K1	0.05196	0.669344	Upregulated in tumor	No
Lung cancer	GSE19804	CCR9	-0.0600567	0.0911797	Downregulated in tumor	No
Lung cancer	GSE19804	SCD	-0.408324	0.0220675	Downregulated in tumor	Yes
Lung cancer	GSE19804	IDO1	-0.271601	0.307409	Downregulated in tumor	No
Lung cancer	GSE19804	SCN1A	-0.503334	3.75e-08	Downregulated in tumor	Yes
Lung cancer	GSE19804	HSD11B1	-0.989366	2.05e-07	Downregulated in tumor	Yes
Lung cancer	GSE19804	PDE2A	-1.31166	1.11e-17	Downregulated in tumor	Yes
Lung cancer	GSE19804	PIK3R2	0.363558	2.03e-13	Upregulated in tumor	Yes

Lung cancer	GSE19804	TRPV1	0.153904	0.0102278	Upregulated in tumor	Yes
Lung cancer	GSE19804	FPR2	-1.57361	1.03e-11	Downregulated in tumor	Yes
Lung cancer	GSE19804	SLC9A1	0.113144	0.0383565	Upregulated in tumor	Yes
Lung cancer	GSE19804	PIK3CG	-0.181785	0.0898347	Downregulated in tumor	No
Lung cancer	GSE19804	GHSR	0.0542378	0.0619084	Upregulated in tumor	No
Lung cancer	GSE19804	CHRNA7	0.011534	0.901188	Upregulated in tumor	No
Lung cancer	GSE19804	MME	-2.29914	9.64e-24	Downregulated in tumor	Yes
Lung cancer	GSE19804	AGTR2	-1.01575	1.47e-08	Downregulated in tumor	Yes
Lung cancer	GSE19804	PER2	-0.207281	0.00809053	Downregulated in tumor	Yes
Lung cancer	GSE19804	BRD4	-0.103331	0.0420434	Downregulated in tumor	Yes
Lung cancer	GSE19804	HDAC6	0.174878	0.000259045	Upregulated in tumor	Yes
Lung cancer	GSE19804	PRMT5	0.477948	6.51e-09	Upregulated in tumor	Yes
Lung cancer	GSE19804	CTSK	0.740253	0.00151663	Upregulated in tumor	Yes
Lung cancer	GSE19804	CA9	0.932448	1.42e-06	Upregulated in tumor	Yes
Lung cancer	GSE19804	SLC6A4	-2.87654	2.25e-19	Downregulated in tumor	Yes
Lung cancer	GSE19804	CA2	-1.10299	0.000505362	Downregulated in tumor	Yes
Prostate cancer	GSE46602	BCL2L1	0.0744059	0.639477	Upregulated in tumor	No
Prostate cancer	GSE46602	NOS2	0.0112029	0.260295	Upregulated in tumor	No
Prostate cancer	GSE46602	ALOX5	-0.0268484	0.88364	Downregulated in tumor	No
Prostate cancer	GSE46602	PIK3CA	-0.179	0.194813	Downregulated in tumor	No
Prostate cancer	GSE46602	CD274	-0.17297	0.166134	Downregulated in tumor	No
Prostate cancer	GSE46602	PCSK9	-0.000911393	0.977245	Downregulated in tumor	No
Prostate cancer	GSE46602	MDM4	-0.147583	0.418535	Downregulated in tumor	No
Prostate cancer	GSE46602	CTSG	0.325696	0.327528	Upregulated in tumor	No
Prostate cancer	GSE46602	FAP	0.967852	0.045332	Upregulated in tumor	Yes
Prostate cancer	GSE46602	PIK3CD	0.131117	0.327097	Upregulated in tumor	No
Prostate cancer	GSE46602	MAP4K1	0.172169	0.470417	Upregulated in tumor	No
Prostate cancer	GSE46602	CCR9	0.0113046	0.233139	Upregulated in tumor	No
Prostate cancer	GSE46602	SCD	-0.505577	0.410074	Downregulated in tumor	No
Prostate cancer	GSE46602	IDO1	0.0779202	0.910291	Upregulated in tumor	No
Prostate cancer	GSE46602	SCN1A	0.0306056	0.276198	Upregulated in tumor	No
Prostate cancer	GSE46602	HSD11B1	-1.12902	0.0109869	Downregulated in tumor	Yes
Prostate cancer	GSE46602	PDE2A	0.0371282	0.845492	Upregulated in tumor	No
Prostate cancer	GSE46602	PIK3R2	0.601474	0.00472751	Upregulated in tumor	Yes
Prostate cancer	GSE46602	TRPV1	0.000109062	0.999615	Upregulated in tumor	No
Prostate cancer	GSE46602	FPR2	0.028341	0.253865	Upregulated in tumor	No
Prostate cancer	GSE46602	SLC9A1	0.154262	0.22147	Upregulated in tumor	No
Prostate cancer	GSE46602	PIK3CG	-0.00064791	0.99801	Downregulated in tumor	No
Prostate cancer	GSE46602	GHSR	0.0184042	0.0859341	Upregulated in tumor	No
Prostate cancer	GSE46602	CHRNA7	-0.21974	0.0743947	Downregulated in tumor	No
Prostate cancer	GSE46602	MME	-3.9358	2.97e-07	Downregulated in tumor	Yes
Prostate cancer	GSE46602	AGTR2	0.0637313	0.166986	Upregulated in tumor	No
Prostate cancer	GSE46602	PER2	-1.05235	0.00161555	Downregulated in tumor	Yes
Prostate cancer	GSE46602	BRD4	-0.132994	0.105829	Downregulated in tumor	No
Prostate cancer	GSE46602	HDAC6	0.0174441	0.903959	Upregulated in tumor	No
Prostate cancer	GSE46602	PRMT5	0.567971	0.0249911	Upregulated in tumor	Yes
Prostate cancer	GSE46602	CTSK	0.228891	0.607334	Upregulated in tumor	No
Prostate cancer	GSE46602	CA9	0.0028172	0.965257	Upregulated in tumor	No
Prostate cancer	GSE46602	SLC6A4	0.0623151	0.242653	Upregulated in tumor	No
Prostate cancer	GSE46602	CA2	-0.0877135	0.892033	Downregulated in tumor	No

Note: log2FC values compare tumour with control samples. FDR values were adjusted using the Benjamini–Hochberg procedure.

Table S3. Non-zero relative feature importance values from the fitted ElasticNet models based on the predefined 34-gene signature.

Cancer type	GEO accession	Gene	Feature rank	Relative feature importance (0–100)
Cervical cancer	GSE63514	PIK3CA	1	100
Cervical cancer	GSE63514	MAP4K1	2	99.3878
Cervical cancer	GSE63514	SLC9A1	3	65.4857
Cervical cancer	GSE63514	CA2	4	57.379
Cervical cancer	GSE63514	CA9	5	53.1638
Cervical cancer	GSE63514	BRD4	6	37.1607
Cervical cancer	GSE63514	PIK3CD	7	35.1318
Cervical cancer	GSE63514	HSD11B1	8	32.6931
Cervical cancer	GSE63514	GHSR	9	32.6921
Cervical cancer	GSE63514	HDAC6	10	9.04432
Cervical cancer	GSE63514	NOS2	11	7.21591
Cervical cancer	GSE63514	MDM4	12	3.04407
Cervical cancer	GSE63514	PRMT5	13	1.33119
Lung cancer	GSE19804	PDE2A	1	100
Lung cancer	GSE19804	FAP	2	81.0767
Lung cancer	GSE19804	CA2	3	58.9739
Lung cancer	GSE19804	MME	4	50.6833
Lung cancer	GSE19804	HSD11B1	5	49.7024
Lung cancer	GSE19804	CD274	6	46.0285
Lung cancer	GSE19804	SCD	7	45.9758
Lung cancer	GSE19804	CCR9	8	45.2422
Lung cancer	GSE19804	SCN1A	9	44.1764
Lung cancer	GSE19804	IDO1	10	41.4981
Lung cancer	GSE19804	BRD4	11	39.0963
Lung cancer	GSE19804	PRMT5	12	38.7356
Lung cancer	GSE19804	PIK3R2	13	37.0426
Lung cancer	GSE19804	SLC6A4	14	30.2005
Lung cancer	GSE19804	PER2	15	29.3311
Lung cancer	GSE19804	MDM4	16	29.0657
Lung cancer	GSE19804	TRPV1	17	25.6016
Lung cancer	GSE19804	ALOX5	18	25.5836
Lung cancer	GSE19804	MAP4K1	19	23.2941
Lung cancer	GSE19804	FPR2	20	20.8317
Lung cancer	GSE19804	AGTR2	21	20.4654
Lung cancer	GSE19804	PIK3CD	22	15.7267
Lung cancer	GSE19804	HDAC6	23	15.3585
Lung cancer	GSE19804	PCSK9	24	15.1648
Lung cancer	GSE19804	NOS2	25	10.7326
Lung cancer	GSE19804	PIK3CG	26	8.8929
Lung cancer	GSE19804	CTSK	27	3.75096
Lung cancer	GSE19804	GHSR	28	3.54191
Lung cancer	GSE19804	CHRNA7	29	2.48205
Lung cancer	GSE19804	PIK3CA	30	2.38342
Lung cancer	GSE19804	CTSG	31	2.35887
Lung cancer	GSE19804	BCL2L1	32	2.26763
Prostate cancer	GSE46602	MME	1	100
Prostate cancer	GSE46602	PIK3R2	2	81.3733
Prostate cancer	GSE46602	CCR9	3	76.1918
Prostate cancer	GSE46602	HSD11B1	4	71.0747
Prostate cancer	GSE46602	PER2	5	66.1165
Prostate cancer	GSE46602	PRMT5	6	64.697
Prostate cancer	GSE46602	GHSR	7	49.8831
Prostate cancer	GSE46602	CHRNA7	8	48.0291
Prostate cancer	GSE46602	FAP	9	46.1231

Prostate cancer	GSE46602	PIK3CA	10	35.1791
Prostate cancer	GSE46602	PIK3CD	11	31.1167
Prostate cancer	GSE46602	SLC9A1	12	23.649
Prostate cancer	GSE46602	NOS2	13	23.1683
Prostate cancer	GSE46602	CTSG	14	15.7512
Prostate cancer	GSE46602	BRD4	15	14.2392
Prostate cancer	GSE46602	AGTR2	16	14.2234
Prostate cancer	GSE46602	IDO1	17	13.5525
Prostate cancer	GSE46602	BCL2L1	18	13.357
Prostate cancer	GSE46602	CD274	19	13.2641
Prostate cancer	GSE46602	CTSK	20	9.5254
Prostate cancer	GSE46602	FPR2	21	8.15712
Prostate cancer	GSE46602	SCD	22	3.20113
Prostate cancer	GSE46602	SLC6A4	23	1.27296
Prostate cancer	GSE46602	SCN1A	24	0.707605

Note: Relative feature importance is scaled from 0 to 100 within each cancer-specific fitted model. It is not an ElasticNet regression coefficient and does not imply independent target validity.

Table S4. Integrated prioritisation of targets for future molecular docking analysis.

Cancer type	GEO accession	Gene	log2FC (tumour vs control)	FDR	Direction	Feature rank	Relative feature importance (0–100)
Cervical cancer	GSE63514	CA9	4.19221	2.61e-08	Upregulated in tumor	5	53.1638
Cervical cancer	GSE63514	PIK3CA	1.47995	1.05e-06	Upregulated in tumor	1	100
Cervical cancer	GSE63514	HSD11B1	2.01127	0.000221065	Upregulated in tumor	8	32.6931
Cervical cancer	GSE63514	MAP4K1	2.01828	0.000381612	Upregulated in tumor	2	99.3878
Cervical cancer	GSE63514	BRD4	0.400823	0.00203304	Upregulated in tumor	6	37.1607
Lung cancer	GSE19804	FAP	1.35783	3.85e-10	Upregulated in tumor	2	81.0767
Lung cancer	GSE19804	PRMT5	0.477948	6.51e-09	Upregulated in tumor	12	38.7356
Lung cancer	GSE19804	CA9	0.932448	1.42e-06	Upregulated in tumor	34	0
Lung cancer	GSE19804	HDAC6	0.174878	0.000259045	Upregulated in tumor	23	15.3585
Lung cancer	GSE19804	CTSK	0.740253	0.00151663	Upregulated in tumor	27	3.75096
Prostate cancer	GSE46602	PRMT5	0.567971	0.0249911	Upregulated in tumor	6	64.697
Prostate cancer	GSE46602	FAP	0.967852	0.045332	Upregulated in tumor	9	46.1231

Note: Targets were retained based on their presence in the network-derived signature, significant tumour-associated expression change in the corresponding GEO dataset, structural tractability, and biological relevance. Relative feature importance reflects the fitted cancer-specific ElasticNet model only.

Table S5. Molecular docking scores, RMSD values, and protein-ligand interaction profiles of the investigated compounds against the standard enzyme targets.

Compound	Target	PDB ID	Binding energy	RMSD	Type and Binding site
Quercetin 3-O-rhamnoside	BChE	6EQP	-10.3	0.8642	**Hydrogen bond:** ASP:70; TYR:128<br>**Hydrophobic interaction:** ALA:328; TYR:332; TRP:430<br>**Pi-pi stacking:** PHE:329
Kaempferol 3-O-glucuronide	BChE	6EQP	-9.5	5.3781	**Hydrogen bond:** ASP:70; TRP:82; TYR:128; PRO:285; TRP:430<br>**Hydrophobic interaction:** TRP:82
Isorhamnetin 3-O-rhamnoside	BChE	6EQP	-9.4	0.1801	**Hydrogen bond:** ASP:70; TRP:82; THR:120; LEU:286<br>**Hydrophobic interaction:** ASP:70; THR:120; VAL:288; PHE:329<br>**Pi-pi stacking:** HIS:438
3,3′,4′,5,7-Pentahydroxyflavone (Quercetin)	AChE	7E3H	-9.3	0.0040	**Hydrogen bond:** ASP:74; TRP:86; GLY:120; TYR:124; GLU:202; TYR:337<br>**Hydrophobic interaction:** TRP:86; TYR:337; PHE:338
Nicotiflorin	BChE	6EQP	-9.3	1.0835	**Hydrogen bond:** TRP:82; GLY:117; THR:120; TYR:128; SER:198; ALA:199; LEU:286<br>**Hydrophobic interaction:** TRP:82; ALA:328; PHE:329; PHE:398<br>**Pi-pi stacking:** TRP:82
Asperulosidic acid	AChE	7E3H	-9.3	0.0887	**Hydrogen bond:** ASP:74; TRP:86; ASN:87; GLY:121; GLY:122; TYR:124; TYR:337; TYR:341; HIS:447<br>**Hydrophobic interaction:** PHE:297; PHE:338<br>**Salt bridge:** HIS:447
Kaempferol 3-O-glucuronide	AChE	7E3H	-9.2	0.8182	**Hydrogen bond:** ASP:74; GLY:120; TYR:124; TYR:133; GLU:202; TYR:337<br>**Hydrophobic interaction:** ASP:74; TRP:86<br>**Pi-pi stacking:** TRP:86; TYR:124
5-O-Feruloylquinic acid	AChE	7E3H	-9.2	2.5931	**Hydrogen bond:** ASP:74; GLY:120; GLY:122; TYR:133; TYR:337<br>**Hydrophobic interaction:** TRP:86; PHE:297; PHE:338

					**Pi-pi stacking:** TRP:86<br>**Salt bridge:** HIS:447
Osthenol	AChE	7E3H	-9.1	0.0034	**Hydrogen bond:** GLY:121; GLY:122; TYR:337<br>**Hydrophobic interaction:** TRP:86; PHE:297; PHE:338; TYR:341
3,3′,4′,5,7-Pentahydroxyflavone (Quercetin)	BChE	6EQP	-9.1	0.3750	**Hydrogen bond:** GLY:115<br>**Hydrophobic interaction:** TRP:82; TRP:430
Asperulosidic acid	BChE	6EQP	-9.0	0.1664	**Hydrogen bond:** TRP:82; GLY:115; GLY:116; THR:120; TYR:128; GLU:197; TRP:430; HIS:438<br>**Hydrophobic interaction:** TRP:82; ALA:328; TRP:430
3-O-Caffeoylquinic acid	AChE	7E3H	-8.8	0.2380	**Hydrogen bond:** GLY:120; TYR:124; TYR:133; GLU:202; HIS:447<br>**Hydrophobic interaction:** TRP:86; PHE:338; TYR:341<br>**Pi-pi stacking:** TRP:86
Cryptochlorogenic acid	AChE	7E3H	-8.7	3.6342	**Hydrogen bond:** TRP:86; TYR:133; PHE:295<br>**Hydrophobic interaction:** TRP:86; TYR:124; PHE:297; PHE:338; TYR:341<br>**Salt bridge:** HIS:447
3-O-Feruloylquinic acid	α-Glucosidase	7KBJ	-8.7	0.0519	**Hydrogen bond:** LEU:252; GLY:253; GLY:254; GLU:287; SER:290; TYR:291; LEU:344; ALA:345<br>**Hydrophobic interaction:** LEU:252; TYR:291; TRP:331<br>**Salt bridge:** LYS:283; ARG:352
Hyperoside	BChE	6EQP	-8.7	1.1186	**Hydrogen bond:** THR:120; TYR:332<br>**Hydrophobic interaction:** VAL:288; PHE:329<br>**Pi-pi stacking:** HIS:438
Uralenneoside	AChE	7E3H	-8.7	1.0688	**Hydrogen bond:** ASP:74; ASN:87; GLY:121; GLY:122; TYR:124; SER:203; TYR:337<br>**Pi-pi stacking:** TRP:86; HIS:447
5-O-Caffeoylquinic acid	α-Glucosidase	7KBJ	-8.7	1.0984	**Hydrogen bond:** TYR:246; GLU:248; LEU:252; GLY:253; GLY:254; LYS:283; GLU:287; ARG:334; LEU:344; ARG:352<br>**Hydrophobic interaction:** TYR:291<br>**Salt bridge:** LYS:283
5-O-Caffeoylquinic acid	BChE	6EQP	-8.6	1.0951	**Hydrogen bond:** ASP:70; TRP:82; ALA:328; TRP:430<br>**Hydrophobic interaction:** LEU:286; PHE:398; TRP:430<br>**Pi-pi stacking:** TRP:231; PHE:329
3-O-Caffeoylquinic acid	BChE	6EQP	-8.5	1.0882	**Hydrogen bond:** TRP:82; TRP:430<br>**Hydrophobic interaction:** ALA:328; PHE:329; PHE:398<br>**Pi-pi stacking:** TRP:231; PHE:329<br>**Salt bridge:** HIS:438
Quercetin 3-O-rhamnoside	AChE	7E3H	-8.5	0.9967	**Hydrogen bond:** TYR:72; ASP:74; TYR:124; TYR:133<br>**Hydrophobic interaction:** ASP:74; TRP:86; TYR:337; PHE:338; TYR:341<br>**Pi-pi stacking:** TRP:86; TYR:124
Cryptochlorogenic acid	α-Glucosidase	7KBJ	-8.4	1.0120	**Hydrogen bond:** ASN:265; LYS:283; TYR:291; ARG:334; LEU:344; TYR:348; ARG:352<br>**Hydrophobic interaction:** LEU:252; LEU:344<br>**Salt bridge:** ARG:334
5-O-Feruloylquinic acid	BChE	6EQP	-8.4	1.2148	**Hydrogen bond:** TRP:82; TYR:128; TYR:332; TRP:430; HIS:438<br>**Hydrophobic interaction:** TRP:82; TYR:440<br>**Salt bridge:** HIS:438
Hyperoside	α-Glucosidase	7KBJ	-8.4	0.4571	**Hydrogen bond:** GLU:248; GLY:253; GLY:254; GLY:264; ASN:265; LEU:344; PHE:350; ARG:352<br>**Hydrophobic interaction:** TYR:291<br>**Salt bridge:** ARG:352
Cryptochlorogenic acid	BChE	6EQP	-8.4	0.5571	**Hydrogen bond:** ASP:70; TRP:82; GLY:115; TYR:128; TRP:430<br>**Hydrophobic interaction:** TRP:82<br>**Pi-pi stacking:** TRP:82
3-O-Caffeoylquinic acid	α-Glucosidase	7KBJ	-8.4	7.4505	**Hydrogen bond:** GLU:287; MET:289; TYR:348; PHE:350; ARG:352<br>**Hydrophobic interaction:** TYR:291<br>**Salt bridge:** ARG:334
5-O-Feruloylquinic acid	α-Glucosidase	7KBJ	-8.4	0.8330	**Hydrogen bond:** GLU:248; ASP:251; LEU:252; GLY:253; GLU:287; ARG:334; ARG:352<br>**Hydrophobic interaction:** LEU:252; TYR:291<br>**Salt bridge:** LYS:283; ARG:352
Quercetin 3-O-rhamnoside	α-Glucosidase	7KBJ	-8.4	4.5565	**Hydrogen bond:** GLY:253; GLY:264; TYR:291; ARG:334; LEU:344; PHE:350; ARG:352<br>**Hydrophobic interaction:** LEU:252; TYR:291; TRP:331; ARG:352<br>**Salt bridge:** ARG:352<br>**Pi-cation interaction:** ARG:352
3-O-Feruloylquinic acid	BChE	6EQP	-8.3	0.9275	**Hydrogen bond:** ASP:70; TRP:82; GLY:115; TYR:128; TYR:332<br>**Hydrophobic interaction:** TRP:82; TYR:332<br>**Pi-pi stacking:** TRP:82
Penstemide	BChE	6EQP	-8.3	0.7847	**Hydrogen bond:** ASP:70; GLY:116; GLY:117; SER:198; ALA:199; TYR:332<br>**Hydrophobic interaction:** TRP:231; LEU:286; VAL:288; PHE:329; PHE:398<br>**Salt bridge:** HIS:438

5-O-Caffeoylquinic acid	AChE	7E3H	-8.3	0.9553	**Hydrogen bond:** GLY:121; TYR:133; GLU:202<br>**Hydrophobic interaction:** TRP:86; TYR:337<br>**Pi-pi stacking:** TYR:341<br>**Salt bridge:** HIS:447
3,3′,4′,5,7-Pentahydroxyflavone (Quercetin)	α-Glucosidase	7KBJ	-8.2	0.2700	**Hydrogen bond:** ALA:345; TYR:348; PHE:350; ARG:352<br>**Hydrophobic interaction:** TYR:291; LEU:344
Osthenol	BChE	6EQP	-8.2	0.0344	**Hydrogen bond:** TRP:82; TRP:430<br>**Hydrophobic interaction:** TRP:82; ALA:328; PHE:329; TYR:332; TRP:430
Uralenneoside	BChE	6EQP	-8.1	2.0854	**Hydrogen bond:** TRP:82; GLY:115; TYR:128; ALA:328; TRP:430<br>**Hydrophobic interaction:** TRP:82<br>**Pi-pi stacking:** TRP:82<br>**Salt bridge:** HIS:438
Hyperoside	AChE	7E3H	-8.0	1.1309	**Hydrogen bond:** TYR:72; GLY:120; GLY:121; TYR:124; TYR:133; GLU:202; TYR:337; HIS:447<br>**Hydrophobic interaction:** ASP:74; TRP:86; TYR:124
Penstemide	α-Glucosidase	7KBJ	-8.0	2.7285	**Hydrogen bond:** MET:289; SER:290; TYR:348; PHE:350<br>**Hydrophobic interaction:** LEU:252; TYR:291<br>**Salt bridge:** ARG:334; ARG:352
3-O-Feruloylquinic acid	AChE	7E3H	-7.8	1.0664	**Hydrogen bond:** GLY:120; GLY:121; TYR:124; TYR:133; GLU:202; TYR:337<br>**Hydrophobic interaction:** TRP:86; TYR:124; PHE:297; PHE:338<br>**Pi-pi stacking:** TRP:86
Scopoletin	AChE	7E3H	-7.7	0.0125	**Hydrogen bond:** TYR:337<br>**Hydrophobic interaction:** TRP:86; TYR:337<br>**Pi-pi stacking:** TRP:86
Quercetin 3-O-(6″-malonyl glucoside)	BChE	6EQP	-7.7	0.9524	**Hydrogen bond:** ASP:70; TRP:82; ASN:83; GLY:117; THR:120; GLY:121; GLU:197; SER:198; SER:287; TRP:430; TYR:440<br>**Hydrophobic interaction:** ASP:70; TRP:82; THR:120; PHE:329<br>**Salt bridge:** HIS:438
Ferulic acid	AChE	7E3H	-7.5	0.0079	**Hydrogen bond:** GLY:120; TYR:133; GLU:202; TYR:337<br>**Hydrophobic interaction:** TRP:86<br>**Pi-pi stacking:** TRP:86
Uralenneoside	α-Glucosidase	7KBJ	-7.5	0.9694	**Hydrogen bond:** GLU:248; GLY:266; LYS:283; SER:290; TYR:291<br>**Hydrophobic interaction:** ILE:257; TYR:291<br>**Salt bridge:** ARG:352
Caffeic acid	AChE	7E3H	-7.4	0.3051	**Hydrogen bond:** GLY:120; TYR:133; GLU:202; TYR:337<br>**Hydrophobic interaction:** TRP:86<br>**Pi-pi stacking:** TRP:86
p-Coumaric acid	AChE	7E3H	-7.2	0.0318	**Hydrogen bond:** GLY:120; TYR:133; TYR:337<br>**Hydrophobic interaction:** TRP:86<br>**Pi-pi stacking:** TRP:86
Asperulosidic acid	α-Glucosidase	7KBJ	-7.1	0.7960	**Hydrogen bond:** GLY:253; TYR:291; ARG:334; TYR:348; PHE:350; ARG:352<br>**Hydrophobic interaction:** TYR:291<br>**Salt bridge:** ARG:352
Scopoletin	BChE	6EQP	-7.0	0.0196	**Hydrogen bond:** GLY:115<br>**Pi-pi stacking:** TRP:82
Kaempferol 3-O-glucuronide	α-Glucosidase	7KBJ	-6.9	7.1010	**Hydrogen bond:** GLU:248; GLY:264; ALA:345; TYR:348; PHE:350; ARG:352<br>**Hydrophobic interaction:** ILE:257; TYR:291; ARG:352<br>**Salt bridge:** ARG:334<br>**Pi-cation interaction:** ARG:352
Quercetin 3-O-(6″-malonyl glucoside)	α-Glucosidase	7KBJ	-6.9	1.0933	**Hydrogen bond:** GLY:253; LYS:283; GLU:287; MET:289; SER:290; TYR:291; TYR:348; PHE:350; ARG:352<br>**Hydrophobic interaction:** LEU:252<br>**Pi-pi stacking:** TYR:291<br>**Salt bridge:** LYS:283; ARG:334
Isorhamnetin 3-O-rhamnoside	AChE	7E3H	-6.8	0.4596	**Hydrogen bond:** TYR:72; ASP:74; ASN:87; TYR:124; TYR:133; GLU:202<br>**Hydrophobic interaction:** TYR:72; ASP:74; TRP:86; TYR:124; TYR:337; TYR:341
Nicotiflorin	α-Glucosidase	7KBJ	-6.8	0.7991	**Hydrogen bond:** GLY:253; LYS:283; PHE:350<br>**Hydrophobic interaction:** LEU:252; ILE:257; TYR:291; TRP:331; TYR:348<br>**Salt bridge:** ARG:352
Osthenol	α-Glucosidase	7KBJ	-6.8	0.0127	**Hydrogen bond:** GLY:253; GLY:254<br>**Hydrophobic interaction:** LEU:252; ILE:257; TYR:291<br>**Salt bridge:** LYS:283; ARG:352<br>**Pi-cation interaction:** ARG:352
Ferulic acid	BChE	6EQP	-6.8	0.0259	**Hydrogen bond:** TRP:82; GLY:115; TRP:430<br>**Hydrophobic interaction:** TRP:82<br>**Pi-pi stacking:** TRP:82

Caffeic acid	BChE	6EQP	-6.7	0.0070	**Hydrogen bond:** SER:198<br>**Hydrophobic interaction:** TRP:82<br>**Pi-pi stacking:** TRP:82
p-Coumaric acid	BChE	6EQP	-6.6	0.6546	**Hydrogen bond:** TRP:82; GLY:115; TRP:430; TYR:440<br>**Hydrophobic interaction:** TRP:82<br>**Pi-pi stacking:** TRP:82
Isorhamnetin 3-O-rhamnoside	α-Glucosidase	7KBJ	-6.5	6.1318	**Hydrogen bond:** GLN:247; GLU:248; TYR:291; ALA:345; PHE:350; ARG:352<br>**Hydrophobic interaction:** TYR:291<br>**Salt bridge:** ARG:352
Nicotiflorin	AChE	7E3H	-6.4	0.1723	**Hydrogen bond:** ASP:74; ASN:87; GLY:120; GLY:121; GLY:122; TYR:124; HIS:447<br>**Hydrophobic interaction:** ASP:74; TRP:86; TYR:124; PHE:295; PHE:297; PHE:338; TYR:341<br>**Pi-pi stacking:** TYR:337<br>**Salt bridge:** HIS:447
Penstemide	AChE	7E3H	-6.3	0.8854	**Hydrogen bond:** GLY:121; TYR:124; GLU:202; SER:203<br>**Hydrophobic interaction:** TYR:72; ASP:74; TYR:124; TYR:337; PHE:338; TYR:341
Scopoletin	α-Glucosidase	7KBJ	-6.3	0.0104	**Hydrogen bond:** TYR:291<br>**Salt bridge:** LYS:283
Caffeic acid	α-Glucosidase	7KBJ	-5.8	0.0546	**Hydrogen bond:** GLY:253; MET:289; TYR:348<br>**Hydrophobic interaction:** ILE:257; TYR:291; TRP:331; LEU:344<br>**Salt bridge:** ARG:334
Ferulic acid	α-Glucosidase	7KBJ	-5.8	0.0428	**Hydrogen bond:** ALA:345<br>**Hydrophobic interaction:** LEU:344; PHE:350<br>**Salt bridge:** ARG:352
p-Coumaric acid	α-Glucosidase	7KBJ	-5.6	0.0822	**Hydrogen bond:** TYR:348<br>**Hydrophobic interaction:** ILE:257; TYR:291; TRP:331; LEU:344<br>**Salt bridge:** ARG:334

Table S6. Molecular docking scores cancer target protein-ligand interaction profiles of the investigated compounds against the selected targets.

Compound	Target	PDB ID	Binding energy	RMSD	Type and Binding site
**Quercetin 3-O-rhamnoside**	PRMT5	5EML	-10.8	23.4896	**Hydrogen bond:** LYS:333; GLY:365; GLU:435; GLU:444; TRP:579<br>**Hydrophobic interaction:** LEU:312; LEU:315; LEU:319; PHE:327
**Kaempferol 3-O-glucuronide**	PRMT5	5EML	-10.5	0.3366	**Hydrogen bond:** LYS:333; TYR:334; GLY:365; ARG:368; GLU:392; GLU:435; TRP:579<br>**Hydrophobic interaction:** LEU:312; PRO:314; LEU:319<br>**Salt bridge:** LYS:333
**3,3′,4′,5,7-Pentahydroxyflavone (Quercetin)**	PRMT5	5EML	-10.4	12.3583	**Hydrogen bond:** TYR:324; LYS:333; GLU:435<br>**Hydrophobic interaction:** TYR:324; PHE:327; LEU:436
**3-O-Caffeoylquinic acid**	PRMT5	5EML	-9.6	1.0844	**Hydrogen bond:** LYS:333; TYR:334; GLY:365; LYS:393; GLU:435<br>**Hydrophobic interaction:** PRO:314; LEU:315; LEU:436<br>**Salt bridge:** LYS:333<br>**Pi-cation interaction:** LYS:393
**Cryptochlorogenic acid**	PRMT5	5EML	-9.5	0.7494	**Hydrogen bond:** GLU:328; LYS:333; TYR:334; ALA:366; ARG:368; GLU:392; LYS:393<br>**Hydrophobic interaction:** PRO:314; LEU:315; LEU:436<br>**Pi-cation interaction:** LYS:393
**Quercetin 3-O-rhamnoside**	MAP4K1	7KAC	-9.5	1.0843	**Hydrogen bond:** TYR:28; LYS:33; CYS:94; SER:98; ASP:155<br>**Hydrophobic interaction:** LEU:23; TYR:28; ALA:141; LEU:144
**Nicotiflorin**	FAP	1Z68	-9.4	23.7039	**Hydrogen bond:** ARG:123; GLU:203; GLN:547; TYR:656<br>**Hydrophobic interaction:** GLN:539; TYR:541; TRP:623
**5-O-Feruloylquinic acid**	PRMT5	5EML	-9.3	7.1641	**Hydrogen bond:** GLN:309; GLU:435<br>**Hydrophobic interaction:** LEU:312; PHE:327; VAL:503<br>**Salt bridge:** LYS:333
**Nicotiflorin**	MAP4K1	7KAC	-9.2	0.5180	**Hydrogen bond:** LEU:23; TYR:28; LYS:33; CYS:94; ASP:155<br>**Hydrophobic interaction:** VAL:31; ALA:44; LYS:46; LEU:144; ALA:154
**Quercetin 3-O-rhamnoside**	BRD4	4QR3	-9.2	0.0636	**Hydrogen bond:** GLN:85; TYR:97; MET:132; ASN:140<br>**Hydrophobic interaction:** TRP:81; PRO:82; PHE:83; LEU:92
**Nicotiflorin**	PRMT5	5EML	-9.2	2.7304	**Hydrogen bond:** ARG:256; ARG:505; HIS:507; PHE:509; GLN:511; HIS:572<br>**Hydrophobic interaction:** LEU:222; PHE:509; GLN:511; PRO:569<br>**Pi-cation interaction:** ARG:505
**Isorhamnetin 3-O-rhamnoside**	MAP4K1	7KAC	-9.1	0.9556	**Hydrogen bond:** TYR:28; LYS:33; CYS:94; ALA:141; ASN:142; ASP:155

					**Hydrophobic interaction:** LEU:23; VAL:31; LYS:46
**Quercetin 3-O-(6″-malonyl glucoside)**	FAP	1Z68	-9.1	25.0097	**Hydrogen bond:** GLN:539; VAL:540; TYR:541; SER:548; ASN:556; TYR:656; ASN:704<br>**Hydrophobic interaction:** GLN:539; TRP:623<br>**Pi-pi stacking:** TRP:623<br>**Salt bridge:** ARG:123; HIS:734
**3-O-Feruloylquinic acid**	PRMT5	5EML	-9.0	9.7906	**Hydrogen bond:** LEU:312; TYR:334; GLU:435<br>**Hydrophobic interaction:** LEU:312; LEU:319; PHE:327<br>**Salt bridge:** LYS:333
**5-O-Caffeoylquinic acid**	PRMT5	5EML	-9.0	14.0163	**Hydrogen bond:** GLN:309; GLU:435; GLU:444; TRP:579; PHE:580<br>**Hydrophobic interaction:** LEU:312; LEU:319; TYR:324; PHE:327<br>**Salt bridge:** LYS:333
**Quercetin 3-O-rhamnoside**	FAP	1Z68	-8.9	0.7340	**Hydrogen bond:** MET:205; PHE:350; PHE:351; THR:354; ILE:400<br>**Hydrophobic interaction:** THR:354<br>**Pi-pi stacking:** PHE:351
**Kaempferol 3-O-glucuronide**	FAP	1Z68	-8.8	1.0212	**Hydrogen bond:** ASN:60; LEU:105; TYR:113; TRP:155<br>**Pi-pi stacking:** TYR:152
**3,3′,4′,5,7-Pentahydroxyflavone (Quercetin)**	BRD4	4QR3	-8.7	2.1554	**Hydrogen bond:** PRO:82; TYR:97; MET:132; ASN:140<br>**Hydrophobic interaction:** TRP:81; PRO:82; VAL:87; LEU:92<br>**Pi-pi stacking:** PHE:83
**Isorhamnetin 3-O-rhamnoside**	FAP	1Z68	-8.7	0.7199	**Hydrogen bond:** GLN:539; SER:624; HIS:734; TYR:745<br>**Hydrophobic interaction:** GLN:539; TYR:541<br>**Pi-pi stacking:** TRP:623
**Isorhamnetin 3-O-rhamnoside**	BRD4	4QR3	-8.7	1.0772	**Hydrogen bond:** GLN:85; MET:132; ASN:140<br>**Hydrophobic interaction:** TRP:81; PRO:82; PHE:83; LEU:92
**Nicotiflorin**	CA9	5FL6	-8.7	7.8930	**Hydrogen bond:** ARG:10; SER:237; ARG:238; LEU:241; PHE:243<br>**Hydrophobic interaction:** TYR:11; PRO:234; LEU:239<br>**Pi-cation interaction:** ARG:10
**Uralenneoside**	PRMT5	5EML	-8.6	5.7884	**Hydrogen bond:** LEU:312; GLU:435; LEU:437; TRP:579<br>**Hydrophobic interaction:** LEU:312; PHE:327
**Kaempferol 3-O-glucuronide**	MAP4K1	7KAC	-8.6	0.3368	**Hydrogen bond:** LYS:33; CYS:94; SER:98; ASP:155<br>**Hydrophobic interaction:** LEU:23; ALA:44; PHE:93; LEU:144; ALA:154; ASP:155
**Hyperoside**	MAP4K1	7KAC	-8.6	1.0242	**Hydrogen bond:** GLY:25; CYS:94; SER:98; ASP:101; ASP:155<br>**Hydrophobic interaction:** VAL:31; LEU:144
**3,3′,4′,5,7-Pentahydroxyflavone (Quercetin)**	MAP4K1	7KAC	-8.6	0.0231	**Hydrogen bond:** LYS:33; CYS:94; ASP:155<br>**Hydrophobic interaction:** LEU:23; LYS:46
**Isorhamnetin 3-O-rhamnoside**	PRMT5	5EML	-8.6	6.5608	**Hydrogen bond:** ASN:295; GLU:305; ARG:505; HIS:507; TYR:554<br>**Hydrophobic interaction:** PHE:509; TYR:554; PRO:569; HIS:572; PRO:581<br>**Salt bridge:** ARG:505<br>**Pi-cation interaction:** HIS:572
**Kaempferol 3-O-glucuronide**	HDAC6	5EDU	-8.5	0.1882	**Hydrogen bond:** ASP:244; LYS:245; LEU:306; LEU:536; GLN:614; ASN:624; ASP:647; GLY:655; THR:656; GLU:779; ASN:654; GLN:634; SER:637; LEU:641; GLU:613; GLU:661; ASP:173; PHE:175; VAL:369; MET:438; MET:489; GLU:502; ARG:606; ASP:497; GLU:543; ALA:550; THR:551; THR:556; SER:563; SER:564; GLU:591; VAL:628; THR:635; ILE:636; VAL:650; GLN:657; SER:670; GLY:686; VAL:703; TRP:705; ASN:706; LEU:716; LEU:722; GLU:757; LEU:765; PRO:804; ALA:760; MET:767; GLY:768; THR:700; ILE:824; TYR:830; TRP:831; PHE:680; TYR:782<br>**Hydrophobic interaction:** TYR:125; TRP:496; LEU:834; PHE:680; LEU:749<br>**Pi-pi stacking:** PHE:680; TYR:125; TYR:570; TRP:496; PHE:620<br>**Metal complex:** ASP:647; ASP:649; HIE:651; SER:670; PHE:660; VAL:666; PHE:699; ASP:742
**Osthenol**	PRMT5	5EML	-8.5	2.0059	**Hydrogen bond:** GLU:328<br>**Hydrophobic interaction:** LEU:312; PHE:327; TRP:579<br>**Pi-pi stacking:** TYR:324<br>**Salt bridge:** LYS:333<br>**Pi-cation interaction:** LYS:333
**Hyperoside**	PRMT5	5EML	-8.5	1.1160	**Hydrogen bond:** GLU:298; ARG:505; HIS:507<br>**Hydrophobic interaction:** PHE:509<br>**Salt bridge:** ARG:505
**Isorhamnetin 3-O-rhamnoside**	HSD11B1	3FCO	-8.4	0.6962	**Hydrogen bond:** SER:43; LYS:44; ILE:46; ARG:66; SER:67; THR:92; MET:93; ILE:121; TYR:147; TYR:183; LYS:187; ILE:218; THR:220; THR:222; THR:124; SER:125; ASP:131; HIS:135; ALA:181; ALA:182<br>**Hydrophobic interaction:** LEU:215; ASP:132<br>**Salt bridge:** LYS:44; ARG:66; HIS:135
**Quercetin 3-O-(6″-malonyl glucoside)**	PRMT5	5EML	-8.4	0.7512	**Hydrogen bond:** GLN:309; SER:310; GLN:313; GLU:320; THR:634<br>**Hydrophobic interaction:** LEU:312; THR:323
**Nicotiflorin**	BRD4	4QR3	-8.3	8.6305	**Hydrogen bond:** LYS:91; LEU:92; ASN:93; TYR:97; ASN:140<br>**Hydrophobic interaction:** TRP:81; PRO:82; PHE:83; VAL:87; LEU:92; TYR:97

**3,3′,4′,5,7-Pentahydroxyflavone (Quercetin)**	FAP	1Z68	-8.2	19.8404	**Hydrogen bond:** GLN:539; TYR:541; SER:548; SER:624; HIS:734<br>**Hydrophobic interaction:** GLN:539; TYR:541<br>**Pi-pi stacking:** TRP:623
**Quercetin 3-O-(6″-malonyl glucoside)**	BRD4	4QR3	-8.2	1.0293	**Hydrogen bond:** GLN:85; TYR:97; ASN:135; TYR:139<br>**Hydrophobic interaction:** TRP:81; PRO:82; VAL:87; LEU:92; TYR:97; TYR:139
**Quercetin 3-O-(6″-malonyl glucoside)**	HSD11B1	3FCO	-8.2	0.9562	**Hydrogen bond:** SER:43; LYS:44; ILE:46; ARG:66; SER:67; THR:92; MET:93; ILE:121; TYR:147; TYR:183; LYS:187; ILE:218; THR:220; THR:222; ASN:123; SER:125; HIS:130; ASP:131; ASP:132; HIS:135; LYS:138; MET:179; ALA:181; ALA:182<br>**Hydrophobic interaction:** LEU:215; ASN:127; PHE:129; ASP:132; HIS:135<br>**Pi-pi stacking:** HIS:135<br>**Salt bridge:** LYS:44; ARG:66; HIS:135
**Quercetin 3-O-(6″-malonyl glucoside)**	CTSK	5Z5O	-8.2	16.5816	**Hydrogen bond:** LYS:20; HIS:62; ASN:117; GLN:120; GLU:285; ASN:286<br>**Pi-pi stacking:** HIS:62<br>**Salt bridge:** HIS:62
**Nicotiflorin**	HDAC6	5EDU	-8.2	0.2100	**Hydrogen bond:** ASP:244; LYS:245; LEU:306; LEU:536; GLN:614; ASN:624; ASP:647; GLY:655; THR:656; GLU:779; ASN:654; GLN:634; SER:637; LEU:641; GLU:613; GLU:661; ASP:173; PHE:175; VAL:369; MET:438; MET:489; GLU:502; ARG:606; ASP:497; GLU:543; ALA:550; THR:551; THR:556; SER:563; SER:564; GLU:591; VAL:628; THR:635; ILE:636; VAL:650; GLN:657; SER:670; GLY:686; VAL:703; TRP:705; ASN:706; LEU:716; LEU:722; GLU:757; LEU:765; PRO:804; ALA:760; MET:767; GLY:768; THR:700; ILE:824; TYR:830; TRP:831; SER:568; ILE:569; TYR:782<br>**Hydrophobic interaction:** TYR:125; TRP:496; LEU:834; PHE:620; LEU:749<br>**Pi-pi stacking:** PHE:680; TYR:125; TYR:570; TRP:496<br>**Metal complex:** ASP:647; ASP:649; HIE:651; SER:670; PHE:660; VAL:666; PHE:699; ASP:742
**Quercetin 3-O-rhamnoside**	HSD11B1	3FCO	-8.2	0.9714	**Hydrogen bond:** SER:43; LYS:44; ILE:46; ARG:66; SER:67; THR:92; MET:93; ILE:121; TYR:147; TYR:183; LYS:187; ILE:218; THR:220; THR:222; LEU:128; HIS:130; ASP:131; ASP:132; ALA:181; ALA:182<br>**Hydrophobic interaction:** LEU:215; ASN:127; PHE:129; ASP:132; HIS:135<br>**Pi-pi stacking:** HIS:135<br>**Salt bridge:** LYS:44; ARG:66<br>**Pi-cation interaction:** HIS:135
**Nicotiflorin**	CTSK	5Z5O	-8.2	3.0282	**Hydrogen bond:** SER:50; GLU:57; LYS:246; GLU:285; ASN:286; GLY:288; ASN:289; LYS:290<br>**Hydrophobic interaction:** GLU:57; ALA:59<br>**Salt bridge:** LYS:290
**Isorhamnetin 3-O-rhamnoside**	CTSK	5Z5O	-8.2	0.4876	**Hydrogen bond:** GLU:57; LYS:246; ASN:286; GLY:288<br>**Hydrophobic interaction:** ALA:49; GLU:57; ALA:59
**Quercetin 3-O-rhamnoside**	CTSK	5Z5O	-8.2	0.8636	**Hydrogen bond:** LYS:37; ALA:59; ASN:61<br>**Hydrophobic interaction:** TYR:41; LEU:63; MET:66
**Hyperoside**	FAP	1Z68	-8.2	34.0995	**Hydrogen bond:** GLN:539; VAL:540; TRP:557; ILE:558; TRP:623; GLY:735; TYR:745<br>**Hydrophobic interaction:** TRP:621; TRP:623<br>**Pi-pi stacking:** TRP:623
**Quercetin 3-O-(6″-malonyl glucoside)**	MAP4K1	7KAC	-8.1	0.5058	**Hydrogen bond:** ASP:137; ILE:138; LYS:139; SER:200; ARG:262<br>**Hydrophobic interaction:** TYR:232<br>**Pi-pi stacking:** TRP:199
**Kaempferol 3-O-glucuronide**	BRD4	4QR3	-8.1	0.0751	**Hydrogen bond:** TYR:139<br>**Hydrophobic interaction:** PHE:83; VAL:87; LEU:92; TYR:97; TYR:139; ILE:146
**5-O-Feruloylquinic acid**	BRD4	4QR3	-8.0	0.9854	**Hydrogen bond:** PRO:82; GLN:85; ASP:88; ASN:140<br>**Hydrophobic interaction:** PRO:82; PHE:83; VAL:87; LEU:92; TYR:97; ILE:146<br>**Salt bridge:** LYS:91
**Nicotiflorin**	HSD11B1	3FCO	-8.0	11.1862	**Hydrogen bond:** SER:43; LYS:44; ILE:46; ARG:66; SER:67; THR:92; MET:93; ILE:121; TYR:147; TYR:183; LYS:187; ILE:218; THR:220; THR:222; ARG:198; ILE:210; GLU:254<br>**Hydrophobic interaction:** LEU:215; ASN:24; ARG:198; ARG:252; GLU:254<br>**Salt bridge:** LYS:44; ARG:66; ARG:252
**Osthenol**	BRD4	4QR3	-8.0	0.1620	**Hydrophobic interaction:** TRP:81; PRO:82; VAL:87; LEU:92; ILE:146<br>**Pi-pi stacking:** PHE:83; TYR:97
**Quercetin 3-O-rhamnoside**	CA9	5FL6	-8.0	16.8230	**Hydrogen bond:** GLY:12; ASP:14; TRP:17; ARG:244; ALA:245<br>**Hydrophobic interaction:** TYR:11; PRO:16; ARG:244; ALA:245<br>**Pi-pi stacking:** TRP:17<br>**Pi-cation interaction:** ARG:244
**Hyperoside**	HSD11B1	3FCO	-7.9	1.0453	**Hydrogen bond:** SER:43; LYS:44; ILE:46; ARG:66; SER:67; THR:92; MET:93; ILE:121; TYR:147; TYR:183; LYS:187; ILE:218; THR:220; THR:222; THR:124; SER:125; ASN:127; ASP:131; HIS:135; ALA:181; ALA:182<br>**Hydrophobic interaction:** LEU:215; PHE:129; ASP:132; ALA:182<br>**Salt bridge:** LYS:44; ARG:66
**Hyperoside**	CA9	5FL6	-7.9	3.6985	**Hydrogen bond:** TYR:11; HIS:68; SER:237; ARG:238; GLN:240<br>**Hydrophobic interaction:** PRO:234; LEU:239<br>**Pi-cation interaction:** ARG:10
**Cryptochlorogenic acid**	FAP	1Z68	-7.9	1.1519	**Hydrogen bond:** LEU:206; PHE:350; PHE:351; GLU:396; ASN:399

					**Hydrophobic interaction:** PHE:351<br>**Pi-pi stacking:** PHE:351
**3-O-Caffeoylquinic acid**	FAP	1Z68	-7.9	0.7275	**Hydrogen bond:** SER:294; PHE:350; PHE:351; ILE:398; ASN:399<br>**Hydrophobic interaction:** PHE:351<br>**Pi-pi stacking:** PHE:351
**Asperulosidic acid**	MAP4K1	7KAC	-7.8	1.0970	**Hydrogen bond:** LYS:33; CYS:94; ASP:155<br>**Hydrophobic interaction:** VAL:75; LEU:144; ALA:154; ASP:155
**Kaempferol 3-O-glucuronide**	CA9	5FL6	-7.8	1.0389	**Hydrogen bond:** GLY:12; GLY:13; ASP:14; TRP:17; ALA:245<br>**Hydrophobic interaction:** PRO:18; ARG:244<br>**Pi-pi stacking:** TRP:17
**3-O-Feruloylquinic acid**	FAP	1Z68	-7.8	0.8559	**Hydrogen bond:** GLU:204; TYR:541; GLN:547; SER:548; SER:624; TYR:656; ASN:704<br>**Hydrophobic interaction:** PHE:350; TYR:541<br>**Pi-pi stacking:** TYR:541<br>**Salt bridge:** ARG:123
**Quercetin 3-O-(6′′-malonyl glucoside)**	CA9	5FL6	-7.7	27.3776	**Hydrogen bond:** GLY:12; TRP:17; ARG:102; GLY:111; ARG:113<br>**Hydrophobic interaction:** ALA:245<br>**Salt bridge:** ARG:113
**3,3′,4′,5,7-Pentahydroxyflavone (Quercetin)**	HDAC6	5EDU	-7.7	0.0106	**Hydrogen bond:** ASP:244; LYS:245; LEU:306; LEU:536; GLN:614; ASN:624; ASP:647; GLY:655; THR:656; GLU:779; ASN:654; GLN:634; SER:637; LEU:641; GLU:613; GLU:661; ASP:173; PHE:175; VAL:369; MET:438; MET:489; GLU:502; ARG:606; ASP:497; GLU:543; ALA:550; THR:551; THR:556; SER:563; SER:564; GLU:591; VAL:628; THR:635; ILE:636; VAL:650; GLN:657; SER:670; GLY:686; VAL:703; TRP:705; ASN:706; LEU:716; LEU:722; GLU:757; LEU:765; PRO:804; ALA:760; MET:767; GLY:768; THR:700; ILE:824; TYR:830; TRP:831; SER:568; PHE:680; TYR:782<br>**Hydrophobic interaction:** TYR:125; TRP:496; LEU:834; PHE:680; LEU:749<br>**Pi-pi stacking:** PHE:680; TYR:125; TYR:570; TRP:496; PHE:620<br>**Metal complex:** ASP:647; ASP:649; HIE:651; SER:670; PHE:660; VAL:666; PHE:699; ASP:742
**3-O-Caffeoylquinic acid**	CA9	5FL6	-7.7	0.9561	**Hydrogen bond:** ALA:99; GLY:101; ARG:102; HIS:112; GLU:148; SER:228<br>**Hydrophobic interaction:** PRO:115; GLU:148; LEU:224
**Isorhamnetin 3-O-rhamnoside**	CA9	5FL6	-7.7	0.1905	**Hydrogen bond:** GLY:98; ALA:99; GLU:149<br>**Hydrophobic interaction:** PRO:103; PRO:115; LEU:224
**Penstemide**	MAP4K1	7KAC	-7.7	0.0477	**Hydrogen bond:** LYS:33; CYS:94; SER:98; ALA:141<br>**Hydrophobic interaction:** LEU:23; VAL:31; VAL:75; LEU:144
**Caffeic acid**	HDAC6	5EDU	-7.7	0.0212	**Hydrogen bond:** ASP:244; LYS:245; LEU:306; LEU:536; GLN:614; ASN:624; ASP:647; GLY:655; THR:656; GLU:779; ASN:654; GLN:634; SER:637; LEU:641; GLU:613; GLU:661; ASP:173; PHE:175; VAL:369; MET:438; MET:489; GLU:502; ARG:606; ASP:497; GLU:543; ALA:550; THR:551; THR:556; SER:563; SER:564; GLU:591; VAL:628; THR:635; ILE:636; VAL:650; GLN:657; SER:670; GLY:686; VAL:703; TRP:705; ASN:706; LEU:716; LEU:722; GLU:757; LEU:765; PRO:804; ALA:760; MET:767; GLY:768; THR:700; ILE:824; TYR:830; TRP:831; SER:568<br>**Hydrophobic interaction:** TYR:125; TRP:496; LEU:834; PHE:620<br>**Pi-pi stacking:** PHE:680; TYR:125; TYR:570; TRP:496<br>**Metal complex:** ASP:647; ASP:649; HIE:651; SER:670; PHE:660; VAL:666; PHE:699; UNL:1; ASP:742
**3-O-Feruloylquinic acid**	MAP4K1	7KAC	-7.7	1.0783	**Hydrogen bond:** TYR:28; LYS:33; CYS:94; ALA:141; ASN:142; ASP:155<br>**Hydrophobic interaction:** VAL:31; ALA:44
**p-Coumaric acid**	HDAC6	5EDU	-7.7	0.0092	**Hydrogen bond:** ASP:244; LYS:245; LEU:306; LEU:536; GLN:614; ASN:624; ASP:647; GLY:655; THR:656; GLU:779; ASN:654; GLN:634; SER:637; LEU:641; GLU:613; GLU:661; ASP:173; PHE:175; VAL:369; MET:438; MET:489; GLU:502; ARG:606; ASP:497; GLU:543; ALA:550; THR:551; THR:556; SER:563; SER:564; GLU:591; VAL:628; THR:635; ILE:636; VAL:650; GLN:657; SER:670; GLY:686; VAL:703; TRP:705; ASN:706; LEU:716; LEU:722; GLU:757; LEU:765; PRO:804; ALA:760; MET:767; GLY:768; THR:700; ILE:824; TYR:830; TRP:831<br>**Hydrophobic interaction:** TYR:125; TRP:496; LEU:834; PHE:620; PHE:680<br>**Pi-pi stacking:** PHE:680; TYR:125; TYR:570; TRP:496<br>**Metal complex:** ASP:647; ASP:649; HIE:651; SER:670; PHE:660; VAL:666; PHE:699; UNL:1; ASP:742
**5-O-Caffeoylquinic acid**	CTSK	5Z5O	-7.7	23.6539	**Hydrogen bond:** GLU:4; GLU:36; SER:43<br>**Hydrophobic interaction:** GLU:36; LYS:40
**Penstemide**	FAP	1Z68	-7.7	16.5519	**Hydrogen bond:** LYS:521; GLN:539; VAL:552; ASN:556; TRP:623<br>**Hydrophobic interaction:** GLN:539; TYR:541; TRP:623
**3,3′,4′,5,7-Pentahydroxyflavone (Quercetin)**	CA9	5FL6	-7.7	0.2804	**Hydrogen bond:** ALA:99; ARG:113; PRO:115; SER:228<br>**Hydrophobic interaction:** PRO:103; PRO:115
**Isorhamnetin 3-O-rhamnoside**	HDAC6	5EDU	-7.7	0.3935	**Hydrogen bond:** ASP:244; LYS:245; LEU:306; LEU:536; GLN:614; ASN:624; ASP:647; GLY:655; THR:656; GLU:779; ASN:654; GLN:634; SER:637; LEU:641; GLU:613; GLU:661; ASP:173; PHE:175; VAL:369; MET:438; MET:489; GLU:502; ARG:606; ASP:497; GLU:543; ALA:550; THR:551; THR:556; SER:563; SER:564; GLU:591; VAL:628; THR:635; ILE:636; VAL:650; GLN:657; SER:670; GLY:686; VAL:703; TRP:705; ASN:706; LEU:716; LEU:722; GLU:757; LEU:765; PRO:804; ALA:760; MET:767; GLY:768; THR:700; ILE:824; TYR:830; TRP:831; SER:568; GLY:619; TYR:782<br>**Hydrophobic interaction:** TYR:125; TRP:496; LEU:834; PHE:679; PHE:680<br>**Pi-pi stacking:** PHE:680; TYR:125; TYR:570; TRP:496; PHE:620<br>**Metal complex:** ASP:647; ASP:649; HIE:651; SER:670; PHE:660; VAL:666; PHE:699; ASP:742
**Kaempferol 3-O-glucuronide**	CTSK	5Z5O	-7.6	22.9163	**Hydrogen bond:** SER:50; GLU:57; LYS:246; GLU:285; ASN:286<br>**Hydrophobic interaction:** THR:55; GLU:57

**Hyperoside**	CTSK	5Z5O	-7.6	0.9028	**Hydrogen bond:** LYS:20; GLN:21; HIS:62; GLU:70; LYS:74; ASN:117; GLN:120<br>**Hydrophobic interaction:** GLN:21<br>**Salt bridge:** LYS:20
**5-O-Caffeoylquinic acid**	FAP	1Z68	-7.6	22.7877	**Hydrogen bond:** ARG:421; GLU:446; ILE:508; SER:551; ALA:554<br>**Hydrophobic interaction:** TYR:418; PRO:469; GLU:506; PHE:553<br>**Salt bridge:** ARG:421
**Scopoletin**	PRMT5	5EML	-7.6	16.3886	**Hydrogen bond:** GLN:309; SER:439; PHE:580<br>**Hydrophobic interaction:** LEU:312; VAL:503<br>**Pi-pi stacking:** PHE:327
**3-O-Caffeoylquinic acid**	MAP4K1	7KAC	-7.6	1.1169	**Hydrogen bond:** LYS:33; CYS:94; SER:98; GLN:100; ALA:141; ASP:155<br>**Hydrophobic interaction:** LEU:23; VAL:31; ALA:44; ALA:141
**Asperulosidic acid**	FAP	1Z68	-7.6	25.6790	**Hydrogen bond:** TRP:122; THR:127; ASN:168; ASN:190<br>**Hydrophobic interaction:** LEU:121; TRP:122; PHE:193; ILE:196
**Cryptochlorogenic acid**	HSD11B1	3FCO	-7.6	21.5221	**Hydrogen bond:** SER:43; LYS:44; ILE:46; ARG:66; SER:67; THR:92; MET:93; ILE:121; TYR:147; TYR:183; LYS:187; ILE:218; THR:220; THR:222; GLU:254; GLU:255; ARG:269; ARG:273<br>**Hydrophobic interaction:** LEU:215; GLU:255; LYS:274<br>**Salt bridge:** LYS:44; ARG:66; ARG:269; LYS:274
**Kaempferol 3-O-glucuronide**	HSD11B1	3FCO	-7.6	34.6722	**Hydrogen bond:** SER:43; LYS:44; ILE:46; ARG:66; SER:67; THR:92; MET:93; ILE:121; TYR:147; TYR:183; LYS:187; ILE:218; THR:220; THR:222; GLN:253; GLU:254; GLU:255; TYR:257; ARG:269<br>**Hydrophobic interaction:** LEU:215; GLN:253<br>**Salt bridge:** LYS:44; ARG:66; ARG:269
**5-O-Feruloylquinic acid**	MAP4K1	7KAC	-7.6	0.7749	**Hydrogen bond:** LYS:33; CYS:94; SER:98; ALA:141; ASP:155<br>**Hydrophobic interaction:** ALA:44; VAL:75; MET:91; LEU:144<br>**Salt bridge:** LYS:33
**Ferulic acid**	HDAC6	5EDU	-7.6	0.0209	**Hydrogen bond:** ASP:244; LYS:245; LEU:306; LEU:536; GLN:614; ASN:624; ASP:647; GLY:655; THR:656; GLU:779; ASN:654; GLN:634; SER:637; LEU:641; GLU:613; GLU:661; ASP:173; PHE:175; VAL:369; MET:438; MET:489; GLU:502; ARG:606; ASP:497; GLU:543; ALA:550; THR:551; THR:556; SER:563; SER:564; GLU:591; VAL:628; THR:635; ILE:636; VAL:650; GLN:657; SER:670; GLY:686; VAL:703; TRP:705; ASN:706; LEU:716; LEU:722; GLU:757; LEU:765; PRO:804; ALA:760; MET:767; GLY:768; THR:700; ILE:824; TYR:830; TRP:831; SER:568<br>**Hydrophobic interaction:** TYR:125; TRP:496; LEU:834; PHE:620<br>**Pi-pi stacking:** PHE:680; TYR:125; TYR:570; TRP:496<br>**Metal complex:** ASP:647; ASP:649; HIE:651; SER:670; PHE:660; VAL:666; PHE:699; UNL:1; ASP:742
**Quercetin 3-O-(6″-malonyl glucoside)**	HDAC6	5EDU	-7.6	0.2441	**Hydrogen bond:** ASP:244; LYS:245; LEU:306; LEU:536; GLN:614; ASN:624; ASP:647; GLY:655; THR:656; GLU:779; ASN:654; GLN:634; SER:637; LEU:641; GLU:613; GLU:661; ASP:173; PHE:175; VAL:369; MET:438; MET:489; GLU:502; ARG:606; ASP:497; GLU:543; ALA:550; THR:551; THR:556; SER:563; SER:564; GLU:591; VAL:628; THR:635; ILE:636; VAL:650; GLN:657; SER:670; GLY:686; VAL:703; TRP:705; ASN:706; LEU:716; LEU:722; GLU:757; LEU:765; PRO:804; ALA:760; MET:767; GLY:768; THR:700; ILE:824; TYR:830; TRP:831; ASN:494; SER:568; ILE:569<br>**Hydrophobic interaction:** TYR:125; TRP:496; LEU:834; PHE:620; PHE:680; LEU:749<br>**Pi-pi stacking:** PHE:680; TYR:125; TYR:570; TRP:496<br>**Metal complex:** ASP:647; ASP:649; HIE:651; SER:670; PHE:660; VAL:666; PHE:699; ASP:742
**5-O-Caffeoylquinic acid**	MAP4K1	7KAC	-7.5	0.4395	**Hydrogen bond:** TYR:28; LYS:33; GLU:92; CYS:94; SER:98; ASN:142; ASP:155<br>**Hydrophobic interaction:** LEU:23; VAL:31; ALA:141; LEU:144
**Osthenol**	CA9	5FL6	-7.5	2.4447	**Hydrogen bond:** GLY:12; ARG:244<br>**Hydrophobic interaction:** TYR:11; TRP:17; ARG:244; ALA:245<br>**Pi-pi stacking:** TRP:17
**Caffeic acid**	PRMT5	5EML	-7.5	1.6968	**Hydrogen bond:** LEU:312; LYS:333; TYR:334; GLU:435; SER:578<br>**Hydrophobic interaction:** PHE:327<br>**Pi-pi stacking:** PHE:327
**Quercetin 3-O-rhamnoside**	HDAC6	5EDU	-7.5	0.6138	**Hydrogen bond:** ASP:244; LYS:245; LEU:306; LEU:536; GLN:614; ASN:624; ASP:647; GLY:655; THR:656; GLU:779; ASN:654; GLN:634; SER:637; LEU:641; GLU:613; GLU:661; ASP:173; PHE:175; VAL:369; MET:438; MET:489; GLU:502; ARG:606; ASP:497; GLU:543; ALA:550; THR:551; THR:556; SER:563; SER:564; GLU:591; VAL:628; THR:635; ILE:636; VAL:650; GLN:657; SER:670; GLY:686; VAL:703; TRP:705; ASN:706; LEU:716; LEU:722; GLU:757; LEU:765; PRO:804; ALA:760; MET:767; GLY:768; THR:700; ILE:824; TYR:830; TRP:831; GLY:619; PHE:680; TYR:782<br>**Hydrophobic interaction:** TYR:125; TRP:496; LEU:834; PRO:501; PHE:680; LEU:749<br>**Pi-pi stacking:** PHE:680; TYR:125; TYR:570; TRP:496; PHE:620<br>**Metal complex:** ASP:647; ASP:649; HIE:651; SER:670; PHE:660; VAL:666; PHE:699; ASP:742
**5-O-Feruloylquinic acid**	FAP	1Z68	-7.5	0.9653	**Hydrogen bond:** GLU:204; SER:548; SER:624; TYR:656; ASN:704<br>**Hydrophobic interaction:** PHE:350; TYR:541<br>**Pi-pi stacking:** TYR:541<br>**Salt bridge:** ARG:123; HIS:734
**Hyperoside**	HDAC6	5EDU	-7.4	0.9603	**Hydrogen bond:** ASP:244; LYS:245; LEU:306; LEU:536; GLN:614; ASN:624; ASP:647; GLY:655; THR:656; GLU:779; ASN:654; GLN:634; SER:637; LEU:641; GLU:613; GLU:661; ASP:173; PHE:175; VAL:369; MET:438; MET:489; GLU:502; ARG:606; ASP:497; GLU:543; ALA:550; THR:551; THR:556; SER:563; SER:564; GLU:591; VAL:628; THR:635; ILE:636; VAL:650; GLN:657; SER:670; GLY:686; VAL:703; TRP:705; ASN:706; LEU:716; LEU:722; GLU:757; LEU:765; PRO:804; ALA:760; MET:767; GLY:768; THR:700; ILE:824; TYR:830; TRP:831; SER:568;

					THR:678; PHE:680; TYR:782<br>**Hydrophobic interaction:** TYR:125; TRP:496; LEU:834; PHE:679; PHE:680<br>**Pi-pi stacking:** PHE:680; TYR:125; TYR:570; TRP:496; PHE:620<br>**Metal complex:** ASP:647; ASP:649; HIE:651; SER:670; PHE:660; VAL:666; PHE:699; ASP:742
**3-O-Caffeoylquinic acid**	HDAC6	5EDU	-7.4	0.3639	**Hydrogen bond:** ASP:244; LYS:245; LEU:306; LEU:536; GLN:614; ASN:624; ASP:647; GLY:655; THR:656; GLU:779; ASN:654; GLN:634; SER:637; LEU:641; GLU:613; GLU:661; ASP:173; PHE:175; VAL:369; MET:438; MET:489; GLU:502; ARG:606; ASP:497; GLU:543; ALA:550; THR:551; THR:556; SER:563; SER:564; GLU:591; VAL:628; THR:635; ILE:636; VAL:650; GLN:657; SER:670; GLY:686; VAL:703; TRP:705; ASN:706; LEU:716; LEU:722; GLU:757; LEU:765; PRO:804; ALA:760; MET:767; GLY:768; THR:700; ILE:824; TYR:830; TRP:831; PHE:680; LEU:749; TYR:782<br>**Hydrophobic interaction:** TYR:125; TRP:496; LEU:834; PHE:679; PHE:680<br>**Pi-pi stacking:** PHE:680; TYR:125; TYR:570; TRP:496; PHE:620<br>**Metal complex:** ASP:647; ASP:649; HIE:651; SER:670; PHE:660; VAL:666; PHE:699; ASP:742
**3-O-Caffeoylquinic acid**	BRD4	4QR3	-7.4	1.0934	**Hydrogen bond:** LEU:92; TYR:97; MET:105; ASN:140<br>**Hydrophobic interaction:** PHE:83; VAL:87; LEU:92; LEU:94
**Osthenol**	HDAC6	5EDU	-7.4	3.1289	**Hydrogen bond:** ASP:244; LYS:245; LEU:306; LEU:536; GLN:614; ASN:624; ASP:647; GLY:655; THR:656; GLU:779; ASN:654; GLN:634; SER:637; LEU:641; GLU:613; GLU:661; ASP:173; PHE:175; VAL:369; MET:438; MET:489; GLU:502; ARG:606; ASP:497; GLU:543; ALA:550; THR:551; THR:556; SER:563; SER:564; GLU:591; VAL:628; THR:635; ILE:636; VAL:650; GLN:657; SER:670; GLY:686; VAL:703; TRP:705; ASN:706; LEU:716; LEU:722; GLU:757; LEU:765; PRO:804; ALA:760; MET:767; GLY:768; THR:700; ILE:824; TYR:830; TRP:831; TYR:782<br>**Hydrophobic interaction:** TYR:125; TRP:496; LEU:834; PHE:620; PHE:679; PHE:680; LEU:749<br>**Pi-pi stacking:** PHE:680; TYR:125; TYR:570; TRP:496<br>**Metal complex:** ASP:647; ASP:649; HIE:651; SER:670; PHE:660; VAL:666; PHE:699; ASP:742
**Cryptochlorogenic acid**	BRD4	4QR3	-7.4	1.0991	**Hydrogen bond:** TYR:97; MET:105; MET:132; ASN:135; ASN:140<br>**Hydrophobic interaction:** PHE:83; VAL:87; ILE:146
**3,3′,4′,5,7-Pentahydroxyflavone (Quercetin)**	HSD11B1	3FCO	-7.4	35.5796	**Hydrogen bond:** SER:43; LYS:44; ILE:46; ARG:66; SER:67; THR:92; MET:93; ILE:121; TYR:147; TYR:183; LYS:187; ILE:218; THR:220; THR:222; SER:125; ASP:132; HIS:135; ALA:181; ALA:182<br>**Hydrophobic interaction:** LEU:215; ASN:127; PHE:129; ASP:132; ALA:182<br>**Salt bridge:** LYS:44; ARG:66
**Ferulic acid**	PRMT5	5EML	-7.3	0.0136	**Hydrogen bond:** LEU:312; LYS:333; TYR:334; GLU:435; SER:439<br>**Hydrophobic interaction:** PHE:327<br>**Pi-pi stacking:** PHE:327
**3-O-Feruloylquinic acid**	BRD4	4QR3	-7.3	0.2628	**Hydrogen bond:** TYR:97; ASN:140<br>**Hydrophobic interaction:** PRO:82; PHE:83; VAL:87; ILE:146
**Cryptochlorogenic acid**	MAP4K1	7KAC	-7.3	0.8065	**Hydrogen bond:** TYR:28; LYS:33; CYS:94; SER:98; ASN:142; ASP:155<br>**Hydrophobic interaction:** LEU:23; ALA:141; LEU:144
**Nicotiflorin**	PIK3CA	2V1Y	-7.3	8.4942	**Hydrogen bond:** GLN:501; ARG:523<br>**Hydrophobic interaction:** GLN:501; ILE:524; ASN:527<br>**Pi-pi stacking:** TYR:508<br>**Salt bridge:** ARG:523
**Penstemide**	PRMT5	5EML	-7.3	22.8769	**Hydrogen bond:** ARG:256; ARG:505; HIS:507; PHE:509<br>**Hydrophobic interaction:** ALA:301<br>**Salt bridge:** ARG:256; ARG:505; HIS:507
**5-O-Caffeoylquinic acid**	BRD4	4QR3	-7.3	0.7424	**Hydrogen bond:** TYR:97; ASN:135<br>**Hydrophobic interaction:** PHE:83; VAL:87; ILE:146
**Asperulosidic acid**	CA9	5FL6	-7.3	1.0409	**Hydrogen bond:** GLY:12; CYS:24; ARG:244; ALA:245; GLN:247<br>**Hydrophobic interaction:** TRP:17<br>**Salt bridge:** ARG:244
**Osthenol**	FAP	1Z68	-7.2	19.0368	**Hydrogen bond:** TRP:623<br>**Hydrophobic interaction:** GLN:539; TYR:541; TRP:623<br>**Pi-pi stacking:** TYR:541
**Cryptochlorogenic acid**	CA9	5FL6	-7.2	0.2890	**Hydrogen bond:** GLY:12; GLN:247<br>**Hydrophobic interaction:** TRP:17; ARG:244<br>**Pi-pi stacking:** TRP:17<br>**Salt bridge:** ARG:244
**Hyperoside**	BRD4	4QR3	-7.2	0.0911	**Hydrogen bond:** ASN:135; ILE:146<br>**Hydrophobic interaction:** PHE:83; VAL:87; LEU:94; TYR:97; TYR:139; ILE:146
**Uralenneoside**	CA9	5FL6	-7.2	1.0418	**Hydrogen bond:** GLY:12; GLY:13; TRP:17; CYS:24; ARG:244; GLN:247<br>**Pi-pi stacking:** TRP:17
**Penstemide**	CA9	5FL6	-7.1	1.5475	**Hydrogen bond:** GLY:12; LEU:241; PHE:243; ALA:245<br>**Hydrophobic interaction:** TRP:17<br>**Salt bridge:** ARG:244
**3,3′,4′,5,7-Pentahydroxyflavone (Quercetin)**	CTSK	5Z5O	-7.1	52.8691	**Hydrogen bond:** LEU:91; TYR:92; ILE:93; GLU:95; LYS:139; ARG:210; ARG:226<br>**Hydrophobic interaction:** ARG:210; LYS:313<br>**Pi-pi stacking:** TYR:92

**3-O-Feruloylquinic acid**	CTSK	5Z5O	-7.1	0.1361	**Hydrogen bond:** SER:245; GLU:285; ASN:286<br>**Hydrophobic interaction:** ALA:59; ASN:286; LYS:290<br>**Salt bridge:** LYS:246
**Osthenol**	HSD11B1	3FCO	-7.1	0.0302	**Hydrogen bond:** SER:43; LYS:44; ILE:46; ARG:66; SER:67; THR:92; MET:93; ILE:121; TYR:147; TYR:183; LYS:187; ILE:218; THR:220; THR:222; THR:124; ASP:132<br>**Hydrophobic interaction:** LEU:215; ASN:127; PHE:129; ASP:132<br>**Salt bridge:** LYS:44; ARG:66; HIS:135
**Uralenneoside**	HSD11B1	3FCO	-7.1	13.2575	**Hydrogen bond:** SER:43; LYS:44; ILE:46; ARG:66; SER:67; THR:92; MET:93; ILE:121; TYR:147; TYR:183; LYS:187; ILE:218; THR:220; THR:222; SER:125; HIS:130; ASP:131; ASP:132; HIS:135; ALA:181; ALA:182<br>**Hydrophobic interaction:** LEU:215; ASN:127; ASP:132<br>**Salt bridge:** LYS:44; ARG:66; HIS:135
**Cryptochlorogenic acid**	HDAC6	5EDU	-7.0	4.2958	**Hydrogen bond:** ASP:244; LYS:245; LEU:306; LEU:536; GLN:614; ASN:624; ASP:647; GLY:655; THR:656; GLU:779; ASN:654; GLN:634; SER:637; LEU:641; GLU:613; GLU:661; ASP:173; PHE:175; VAL:369; MET:438; MET:489; GLU:502; ARG:606; ASP:497; GLU:543; ALA:550; THR:551; THR:556; SER:563; SER:564; GLU:591; VAL:628; THR:635; ILE:636; VAL:650; GLN:657; SER:670; GLY:686; VAL:703; TRP:705; ASN:706; LEU:716; LEU:722; GLU:757; LEU:765; PRO:804; ALA:760; MET:767; GLY:768; THR:700; ILE:824; TYR:830; TRP:831; ASN:494<br>**Hydrophobic interaction:** TYR:125; TRP:496; LEU:834; TYR:570; PHE:620; PHE:680<br>**Pi-pi stacking:** PHE:680; TYR:125; TYR:570; TRP:496<br>**Metal complex:** ASP:647; ASP:649; HIE:651; SER:670; PHE:660; VAL:666; PHE:699; ASP:742
**Asperulosidic acid**	PRMT5	5EML	-7.0	1.1691	**Hydrogen bond:** ARG:164; THR:173; THR:174; HIS:175; GLU:178<br>**Hydrophobic interaction:** GLU:161; ASP:162<br>**Salt bridge:** HIS:175
**p-Coumaric acid**	PRMT5	5EML	-7.0	0.4152	**Hydrogen bond:** LEU:312; LYS:333; GLU:435; SER:439<br>**Hydrophobic interaction:** PHE:327<br>**Pi-pi stacking:** PHE:327
**5-O-Feruloylquinic acid**	CA9	5FL6	-7.0	2.8450	**Hydrogen bond:** GLY:12; ARG:244; GLN:247<br>**Hydrophobic interaction:** TRP:17; ARG:244<br>**Pi-pi stacking:** TRP:17<br>**Salt bridge:** ARG:244
**Asperulosidic acid**	CTSK	5Z5O	-7.0	1.0906	**Hydrogen bond:** THR:55; LYS:246; ASN:286; GLY:288; LYS:290
**3-O-Feruloylquinic acid**	HDAC6	5EDU	-7.0	0.3424	**Hydrogen bond:** ASP:244; LYS:245; LEU:306; LEU:536; GLN:614; ASN:624; ASP:647; GLY:655; THR:656; GLU:779; ASN:654; GLN:634; SER:637; LEU:641; GLU:613; GLU:661; ASP:173; PHE:175; VAL:369; MET:438; MET:489; GLU:502; ARG:606; ASP:497; GLU:543; ALA:550; THR:551; THR:556; SER:563; SER:564; GLU:591; VAL:628; THR:635; ILE:636; VAL:650; GLN:657; SER:670; GLY:686; VAL:703; TRP:705; ASN:706; LEU:716; LEU:722; GLU:757; LEU:765; PRO:804; ALA:760; MET:767; GLY:768; THR:700; ILE:824; TYR:830; TRP:831; ASN:494; TRP:496; SER:568; ILE:569<br>**Hydrophobic interaction:** TYR:125; TRP:496; LEU:834; PRO:501; LEU:749<br>**Pi-pi stacking:** PHE:680; TYR:125; TYR:570; TRP:496; PHE:620<br>**Metal complex:** ASP:647; ASP:649; HIE:651; SER:670; PHE:660; VAL:666; PHE:699; ASP:742
**Penstemide**	HDAC6	5EDU	-7.0	0.6070	**Hydrogen bond:** ASP:244; LYS:245; LEU:306; LEU:536; GLN:614; ASN:624; ASP:647; GLY:655; THR:656; GLU:779; ASN:654; GLN:634; SER:637; LEU:641; GLU:613; GLU:661; ASP:173; PHE:175; VAL:369; MET:438; MET:489; GLU:502; ARG:606; ASP:497; GLU:543; ALA:550; THR:551; THR:556; SER:563; SER:564; GLU:591; VAL:628; THR:635; ILE:636; VAL:650; GLN:657; SER:670; GLY:686; VAL:703; TRP:705; ASN:706; LEU:716; LEU:722; GLU:757; LEU:765; PRO:804; ALA:760; MET:767; GLY:768; THR:700; ILE:824; TYR:830; TRP:831; ASN:494; ILE:569<br>**Hydrophobic interaction:** TYR:125; TRP:496; LEU:834; PHE:620; PHE:680<br>**Pi-pi stacking:** PHE:680; TYR:125; TYR:570; TRP:496<br>**Metal complex:** ASP:647; ASP:649; HIE:651; SER:670; PHE:660; VAL:666; PHE:699; ASP:742
**Scopoletin**	BRD4	4QR3	-7.0	0.0991	**Hydrogen bond:** ASN:140<br>**Hydrophobic interaction:** PRO:82; PHE:83; VAL:87; TYR:97; ILE:146
**5-O-Caffeoylquinic acid**	HSD11B1	3FCO	-7.0	4.9623	**Hydrogen bond:** SER:43; LYS:44; ILE:46; ARG:66; SER:67; THR:92; MET:93; ILE:121; TYR:147; TYR:183; LYS:187; ILE:218; THR:220; THR:222; ASN:127; HIS:130; ASP:131; ASP:132; ALA:181; ALA:182<br>**Hydrophobic interaction:** LEU:215; ASN:127; PHE:129<br>**Salt bridge:** LYS:44; ARG:66; HIS:130<br>**Pi-cation interaction:** HIS:135
**Uralenneoside**	FAP	1Z68	-7.0	24.5511	**Hydrogen bond:** THR:517<br>**Hydrophobic interaction:** TYR:374; GLU:417<br>**Salt bridge:** LYS:394
**3-O-Feruloylquinic acid**	HSD11B1	3FCO	-7.0	1.0681	**Hydrogen bond:** SER:43; LYS:44; ILE:46; ARG:66; SER:67; THR:92; MET:93; ILE:121; TYR:147; TYR:183; LYS:187; ILE:218; THR:220; THR:222; LYS:174; GLU:254; GLU:255; ARG:269; ARG:273; GLU:277<br>**Hydrophobic interaction:** LEU:215; GLN:253; GLU:254; GLU:255; LYS:274<br>**Salt bridge:** LYS:44; ARG:66; ARG:269; LYS:274
**Osthenol**	MAP4K1	7KAC	-7.0	0.0390	**Hydrogen bond:** CYS:94<br>**Hydrophobic interaction:** LEU:23; VAL:31; ALA:44; ALA:154
**Scopoletin**	CA9	5FL6	-6.9	0.0125	**Hydrogen bond:** GLY:12; GLY:13<br>**Hydrophobic interaction:** ARG:244

					**Pi-pi stacking:** TRP:17
**5-O-Caffeoylquinic acid**	CA9	5FL6	-6.9	6.8769	**Hydrogen bond:** GLY:12; GLY:13; TRP:17; CYS:24; ALA:25; ARG:244; ALA:245<br>**Hydrophobic interaction:** TRP:17; PRO:18; ALA:25
**Ferulic acid**	HSD11B1	3FCO	-6.9	10.8620	**Hydrogen bond:** SER:43; LYS:44; ILE:46; ARG:66; SER:67; THR:92; MET:93; ILE:121; TYR:147; TYR:183; LYS:187; ILE:218; THR:220; THR:222<br>**Hydrophobic interaction:** LEU:215; VAL:180<br>**Pi-pi stacking:** TYR:183<br>**Salt bridge:** LYS:44; ARG:66
**Uralenneoside**	BRD4	4QR3	-6.9	1.0491	**Hydrogen bond:** TYR:97; MET:105; ASN:140<br>**Hydrophobic interaction:** PRO:82; ILE:146
**5-O-Feruloylquinic acid**	HDAC6	5EDU	-6.9	0.4101	**Hydrogen bond:** ASP:244; LYS:245; LEU:306; LEU:536; GLN:614; ASN:624; ASP:647; GLY:655; THR:656; GLU:779; ASN:654; GLN:634; SER:637; LEU:641; GLU:613; GLU:661; ASP:173; PHE:175; VAL:369; MET:438; MET:489; GLU:502; ARG:606; ASP:497; GLU:543; ALA:550; THR:551; THR:556; SER:563; SER:564; GLU:591; VAL:628; THR:635; ILE:636; VAL:650; GLN:657; SER:670; GLY:686; VAL:703; TRP:705; ASN:706; LEU:716; LEU:722; GLU:757; LEU:765; PRO:804; ALA:760; MET:767; GLY:768; THR:700; ILE:824; TYR:830; TRP:831; ASN:494; SER:568<br>**Hydrophobic interaction:** TYR:125; TRP:496; LEU:834; PHE:620; LEU:749<br>**Pi-pi stacking:** PHE:680; TYR:125; TYR:570; TRP:496<br>**Metal complex:** ASP:647; ASP:649; HIE:651; SER:670; PHE:660; VAL:666; PHE:699; ASP:742
**Quercetin 3-O-rhamnoside**	PIK3CA	2V1Y	-6.8	0.6901	**Hydrogen bond:** GLN:432; VAL:589; GLN:591; LYS:592<br>**Hydrophobic interaction:** ARG:590; GLN:591<br>**Pi-cation interaction:** ARG:590
**Uralenneoside**	MAP4K1	7KAC	-6.8	0.2424	**Hydrogen bond:** LYS:33; CYS:94; ASP:155<br>**Hydrophobic interaction:** LEU:23
**Penstemide**	CTSK	5Z5O	-6.8	17.5047	**Hydrogen bond:** ASN:286; GLY:288<br>**Hydrophobic interaction:** ALA:49; GLU:57; LYS:290
**3-O-Caffeoylquinic acid**	CTSK	5Z5O	-6.7	34.1819	**Hydrogen bond:** ARG:107; VAL:115; ASN:146; TYR:292<br>**Hydrophobic interaction:** THR:113; LEU:144; LEU:145
**5-O-Feruloylquinic acid**	HSD11B1	3FCO	-6.7	34.7640	**Hydrogen bond:** SER:43; LYS:44; ILE:46; ARG:66; SER:67; THR:92; MET:93; ILE:121; TYR:147; TYR:183; LYS:187; ILE:218; THR:220; THR:222; LYS:174; GLN:253; GLU:254; ARG:273<br>**Hydrophobic interaction:** LEU:215; GLU:254; LYS:274<br>**Salt bridge:** LYS:44; ARG:66; ARG:269; LYS:274
**3-O-Feruloylquinic acid**	CA9	5FL6	-6.7	27.0316	**Hydrogen bond:** GLY:101; ARG:102; HIS:112; GLU:148; SER:228<br>**Hydrophobic interaction:** PRO:115; GLU:148; GLU:149; LEU:224
**5-O-Caffeoylquinic acid**	HDAC6	5EDU	-6.7	0.9203	**Hydrogen bond:** ASP:244; LYS:245; LEU:306; LEU:536; GLN:614; ASN:624; ASP:647; GLY:655; THR:656; GLU:779; ASN:654; GLN:634; SER:637; LEU:641; GLU:613; GLU:661; ASP:173; PHE:175; VAL:369; MET:438; MET:489; GLU:502; ARG:606; ASP:497; GLU:543; ALA:550; THR:551; THR:556; SER:563; SER:564; GLU:591; VAL:628; THR:635; ILE:636; VAL:650; GLN:657; SER:670; GLY:686; VAL:703; TRP:705; ASN:706; LEU:716; LEU:722; GLU:757; LEU:765; PRO:804; ALA:760; MET:767; GLY:768; THR:700; ILE:824; TYR:830; TRP:831; PHE:680<br>**Hydrophobic interaction:** TYR:125; TRP:496; LEU:834; PRO:501; PHE:620; LEU:749<br>**Pi-pi stacking:** PHE:680; TYR:125; TYR:570; TRP:496; PHE:620<br>**Metal complex:** ASP:647; ASP:649; HIE:651; SER:670; PHE:660; VAL:666; PHE:699; ASP:742
**Uralenneoside**	CTSK	5Z5O	-6.7	4.6589	**Hydrogen bond:** THR:55; ASN:286; LYS:290<br>**Hydrophobic interaction:** ALA:49; GLU:57
**Scopoletin**	HDAC6	5EDU	-6.6	0.0596	**Hydrogen bond:** ASP:244; LYS:245; LEU:306; LEU:536; GLN:614; ASN:624; ASP:647; GLY:655; THR:656; GLU:779; ASN:654; GLN:634; SER:637; LEU:641; GLU:613; GLU:661; ASP:173; PHE:175; VAL:369; MET:438; MET:489; GLU:502; ARG:606; ASP:497; GLU:543; ALA:550; THR:551; THR:556; SER:563; SER:564; GLU:591; VAL:628; THR:635; ILE:636; VAL:650; GLN:657; SER:670; GLY:686; VAL:703; TRP:705; ASN:706; LEU:716; LEU:722; GLU:757; LEU:765; PRO:804; ALA:760; MET:767; GLY:768; THR:700; ILE:824; TYR:830; TRP:831; TYR:782<br>**Hydrophobic interaction:** TYR:125; TRP:496; LEU:834; PHE:680<br>**Pi-pi stacking:** PHE:680; TYR:125; TYR:570; TRP:496; PHE:620<br>**Metal complex:** ASP:647; ASP:649; HIE:651; SER:670; PHE:660; VAL:666; PHE:699; ASP:742
**Scopoletin**	PIK3CA	2V1Y	-6.6	2.2707	**Hydrogen bond:** ASN:564<br>**Hydrophobic interaction:** LEU:466; TYR:467; TYR:470<br>**Pi-pi stacking:** TYR:463; TYR:467<br>**Pi-cation interaction:** LYS:567
**5-O-Feruloylquinic acid**	CTSK	5Z5O	-6.6	50.6543	**Hydrogen bond:** LYS:37; ALA:59; ASN:61; LEU:63; THR:67; SER:68<br>**Pi-pi stacking:** TYR:41
**Hyperoside**	PIK3CA	2V1Y	-6.6	2.1990	**Hydrogen bond:** GLN:497; GLN:501; TYR:504; ASN:527<br>**Hydrophobic interaction:** TYR:504; TYR:508; ILE:524
**Osthenol**	CTSK	5Z5O	-6.5	15.6219	**Hydrogen bond:** TYR:92; LYS:313<br>**Hydrophobic interaction:** ARG:210; ARG:226; VAL:227; PHE:311; LYS:313
**Ferulic acid**	BRD4	4QR3	-6.5	0.0198	**Hydrogen bond:** PRO:82<br>**Hydrophobic interaction:** PRO:82; PHE:83; VAL:87; ILE:146
**3-O-Caffeoylquinic acid**	HSD11B1	3FCO	-6.5	40.4884	**Hydrogen bond:** SER:43; LYS:44; ILE:46; ARG:66; SER:67; THR:92; MET:93; ILE:121; TYR:147; TYR:183; LYS:187; ILE:218; THR:220; THR:222; ASN:127; LEU:128; ASP:132; ALA:181; ALA:182<br>**Hydrophobic interaction:** LEU:215; ASN:127; PHE:129; ASP:132

					**Pi-pi stacking:** HIS:135<br>**Salt bridge:** LYS:44; ARG:66; HIS:130
**Cryptochlorogenic acid**	CTSK	5Z5O	-6.5	42.2673	**Hydrogen bond:** VAL:72; LYS:74; THR:76; GLY:77; LEU:78; GLY:122<br>**Hydrophobic interaction:** VAL:72
**Penstemide**	BRD4	4QR3	-6.5	0.2751	**Hydrogen bond:** ASP:144<br>**Hydrophobic interaction:** PRO:82; PHE:83; VAL:87; LEU:92; LEU:94
**Penstemide**	HSD11B1	3FCO	-6.5	1.0345	**Hydrogen bond:** SER:43; LYS:44; ILE:46; ARG:66; SER:67; THR:92; MET:93; ILE:121; TYR:147; TYR:183; LYS:187; ILE:218; THR:220; THR:222; ASN:24; VAL:206; VAL:208; ARG:252; GLU:254<br>**Hydrophobic interaction:** LEU:215; ASN:24; ARG:252; GLN:253<br>**Salt bridge:** LYS:44; ARG:66
**Kaempferol 3-O-glucuronide**	PIK3CA	2V1Y	-6.5	6.4982	**Hydrogen bond:** GLN:501; TYR:504; ARG:523; ASN:527<br>**Hydrophobic interaction:** GLN:501; TYR:504; ILE:524<br>**Salt bridge:** ARG:523
**Asperulosidic acid**	BRD4	4QR3	-6.5	4.1912	**Hydrogen bond:** TYR:97; MET:132; ASN:140<br>**Hydrophobic interaction:** TRP:81; PRO:82; LEU:92; ILE:146
**Caffeic acid**	CA9	5FL6	-6.4	1.6287	**Hydrogen bond:** GLY:12; GLY:13<br>**Hydrophobic interaction:** TRP:17; ARG:244<br>**Pi-pi stacking:** TRP:17<br>**Salt bridge:** ARG:244
**Isorhamnetin 3-O-rhamnoside**	PIK3CA	2V1Y	-6.4	1.0977	**Hydrogen bond:** GLN:497; GLN:501; TYR:508<br>**Hydrophobic interaction:** TYR:504; ILE:524; ASN:527<br>**Pi-pi stacking:** TYR:508
**Caffeic acid**	HSD11B1	3FCO	-6.4	11.7484	**Hydrogen bond:** SER:43; LYS:44; ILE:46; ARG:66; SER:67; THR:92; MET:93; ILE:121; TYR:147; TYR:183; LYS:187; ILE:218; THR:220; THR:222; SER:170<br>**Hydrophobic interaction:** LEU:215; TYR:183<br>**Salt bridge:** LYS:44; ARG:66
**Uralenneoside**	HDAC6	5EDU	-6.4	1.0629	**Hydrogen bond:** ASP:244; LYS:245; LEU:306; LEU:536; GLN:614; ASN:624; ASP:647; GLY:655; THR:656; GLU:779; ASN:654; GLN:634; SER:637; LEU:641; GLU:613; GLU:661; ASP:173; PHE:175; VAL:369; MET:438; MET:489; GLU:502; ARG:606; ASP:497; GLU:543; ALA:550; THR:551; THR:556; SER:563; SER:564; GLU:591; VAL:628; THR:635; ILE:636; VAL:650; GLN:657; SER:670; GLY:686; VAL:703; TRP:705; ASN:706; LEU:716; LEU:722; GLU:757; LEU:765; PRO:804; ALA:760; MET:767; GLY:768; THR:700; ILE:824; TYR:830; TRP:831; THR:678; PHE:680<br>**Hydrophobic interaction:** TYR:125; TRP:496; LEU:834; PHE:620; PHE:680<br>**Pi-pi stacking:** PHE:680; TYR:125; TYR:570; TRP:496<br>**Metal complex:** ASP:647; ASP:649; HIE:651; SER:670; PHE:660; VAL:666; PHE:699; ASP:742
**Scopoletin**	HSD11B1	3FCO	-6.3	13.3370	**Hydrogen bond:** SER:43; LYS:44; ILE:46; ARG:66; SER:67; THR:92; MET:93; ILE:121; TYR:147; TYR:183; LYS:187; ILE:218; THR:220; THR:222; HIS:130; ASP:131; ASP:132; ALA:181; ALA:182<br>**Hydrophobic interaction:** LEU:215; ASN:127; PHE:129; ASP:132<br>**Salt bridge:** LYS:44; ARG:66
**Asperulosidic acid**	HDAC6	5EDU	-6.3	1.0872	**Hydrogen bond:** ASP:244; LYS:245; LEU:306; LEU:536; GLN:614; ASN:624; ASP:647; GLY:655; THR:656; GLU:779; ASN:654; GLN:634; SER:637; LEU:641; GLU:613; GLU:661; ASP:173; PHE:175; VAL:369; MET:438; MET:489; GLU:502; ARG:606; ASP:497; GLU:543; ALA:550; THR:551; THR:556; SER:563; SER:564; GLU:591; VAL:628; THR:635; ILE:636; VAL:650; GLN:657; SER:670; GLY:686; VAL:703; TRP:705; ASN:706; LEU:716; LEU:722; GLU:757; LEU:765; PRO:804; ALA:760; MET:767; GLY:768; THR:700; ILE:824; TYR:830; TRP:831; SER:568; PHE:680; TYR:782<br>**Hydrophobic interaction:** TYR:125; TRP:496; LEU:834; PHE:620; PHE:680<br>**Pi-pi stacking:** PHE:680; TYR:125; TYR:570; TRP:496<br>**Metal complex:** ASP:647; ASP:649; HIE:651; SER:670; PHE:660; VAL:666; PHE:699; ASP:742
**Asperulosidic acid**	HSD11B1	3FCO	-6.3	0.9896	**Hydrogen bond:** SER:43; LYS:44; ILE:46; ARG:66; SER:67; THR:92; MET:93; ILE:121; TYR:147; TYR:183; LYS:187; ILE:218; THR:220; THR:222; SER:125; ASN:127; ASP:132<br>**Hydrophobic interaction:** LEU:215; HIS:134<br>**Salt bridge:** LYS:44; ARG:66; HIS:135
**Ferulic acid**	CA9	5FL6	-6.3	0.0259	**Hydrogen bond:** GLY:12; GLY:13; GLN:247<br>**Hydrophobic interaction:** TRP:17<br>**Pi-pi stacking:** TRP:17<br>**Salt bridge:** ARG:244
**Ferulic acid**	CTSK	5Z5O	-6.2	0.1011	**Hydrogen bond:** ILE:93; LYS:139; ARG:226<br>**Hydrophobic interaction:** VAL:227; PHE:311<br>**Salt bridge:** ARG:210
**Caffeic acid**	FAP	1Z68	-6.2	31.4905	**Hydrogen bond:** GLY:348; SER:369<br>**Hydrophobic interaction:** GLU:340; PHE:368; VAL:384<br>**Salt bridge:** LYS:375
**Scopoletin**	FAP	1Z68	-6.2	26.7012	**Hydrogen bond:** ASN:413; LYS:510; THR:517<br>**Hydrophobic interaction:** GLN:580
**Caffeic acid**	BRD4	4QR3	-6.2	2.4324	**Hydrogen bond:** PRO:82

					**Hydrophobic interaction:** PRO:82; PHE:83; VAL:87; ILE:146
**Uralenneoside**	PIK3CA	2V1Y	-6.2	0.4598	**Hydrogen bond:** ASN:564<br>**Hydrophobic interaction:** TYR:467; TYR:470<br>**Salt bridge:** LYS:567
**Cryptochlorogenic acid**	PIK3CA	2V1Y	-6.1	52.9734	**Hydrogen bond:** GLU:502; GLU:510; HIS:526<br>**Hydrophobic interaction:** LYS:506; ILE:509; ILE:524; TYR:528
**p-Coumaric acid**	CA9	5FL6	-6.1	1.0017	**Hydrogen bond:** GLY:12; GLN:247<br>**Hydrophobic interaction:** TRP:17<br>**Pi-pi stacking:** TRP:17<br>**Salt bridge:** ARG:244
**Ferulic acid**	FAP	1Z68	-6.1	0.0341	**Hydrogen bond:** GLY:348; SER:369<br>**Hydrophobic interaction:** GLU:340; PHE:368; VAL:384<br>**Salt bridge:** LYS:375
**p-Coumaric acid**	PIK3CA	2V1Y	-6.1	0.3704	**Hydrophobic interaction:** TYR:463; LEU:466; TYR:467; TYR:470<br>**Pi-pi stacking:** TYR:470
**5-O-Caffeoylquinic acid**	PIK3CA	2V1Y	-6.0	3.4255	**Hydrogen bond:** GLN:501; GLU:507; ILE:524<br>**Hydrophobic interaction:** GLN:501; TYR:504; TYR:508; ILE:524<br>**Pi-pi stacking:** TYR:508
**3,3′,4′,5,7-Pentahydroxyflavone (Quercetin)**	PIK3CA	2V1Y	-6.0	5.9099	**Hydrogen bond:** TRP:583; THR:585; GLN:586<br>**Hydrophobic interaction:** TYR:431; ASP:578; LEU:581; THR:585
**3-O-Feruloylquinic acid**	PIK3CA	2V1Y	-6.0	30.9372	**Hydrogen bond:** THR:490; SER:541; ARG:544; LEU:545; ASP:548<br>**Hydrophobic interaction:** ALA:483; ALA:486; PHE:487<br>**Pi-pi stacking:** PHE:487
**p-Coumaric acid**	FAP	1Z68	-6.0	0.0147	**Hydrogen bond:** GLY:348; SER:369<br>**Hydrophobic interaction:** GLU:340; PHE:368; VAL:384<br>**Salt bridge:** LYS:375
**Quercetin 3-O-(6″-malonyl glucoside)**	PIK3CA	2V1Y	-6.0	1.0809	**Hydrogen bond:** GLN:497; ASN:527<br>**Hydrophobic interaction:** TYR:504; ILE:524; ASN:527<br>**Salt bridge:** ARG:523
**Scopoletin**	MAP4K1	7KAC	-6.0	0.0136	**Hydrogen bond:** CYS:94<br>**Hydrophobic interaction:** LEU:23<br>**Salt bridge:** LYS:33
**Caffeic acid**	MAP4K1	7KAC	-5.9	1.7555	**Hydrogen bond:** LYS:33; CYS:94<br>**Hydrophobic interaction:** LEU:23; ALA:44
**Osthenol**	PIK3CA	2V1Y	-5.9	0.0481	**Hydrogen bond:** GLN:497; ARG:534<br>**Hydrophobic interaction:** PHE:494; GLN:497; LYS:530; LEU:531; ARG:534
**Scopoletin**	CTSK	5Z5O	-5.9	34.8198	**Hydrogen bond:** LYS:140<br>**Hydrophobic interaction:** GLN:136; LEU:137; LEU:147; ILE:180
**p-Coumaric acid**	BRD4	4QR3	-5.9	6.8347	**Hydrophobic interaction:** PRO:82; PHE:83; VAL:87; ILE:146
**Ferulic acid**	MAP4K1	7KAC	-5.8	0.0853	**Hydrogen bond:** LYS:33; CYS:94<br>**Hydrophobic interaction:** LEU:23; VAL:31; ALA:44
**Penstemide**	PIK3CA	2V1Y	-5.8	2.9573	**Hydrogen bond:** GLN:435; VAL:437; GLU:443<br>**Hydrophobic interaction:** GLU:439; VAL:589; ARG:590; LYS:592; LYS:593<br>**Salt bridge:** LYS:593
**Caffeic acid**	CTSK	5Z5O	-5.8	9.1777	**Hydrogen bond:** GLU:36; SER:43<br>**Hydrophobic interaction:** GLU:36; LEU:39; LYS:40
**p-Coumaric acid**	HSD11B1	3FCO	-5.7	5.8196	**Hydrogen bond:** SER:43; LYS:44; ILE:46; ARG:66; SER:67; THR:92; MET:93; ILE:121; TYR:147; TYR:183; LYS:187; ILE:218; THR:220; THR:222; THR:124; ALA:182<br>**Hydrophobic interaction:** LEU:215; ASN:127; PHE:129; ASP:132<br>**Salt bridge:** LYS:44; ARG:66; HIS:135
**3-O-Caffeoylquinic acid**	PIK3CA	2V1Y	-5.7	56.7782	**Hydrogen bond:** GLN:501; SER:505; ARG:523<br>**Hydrophobic interaction:** ARG:523; ILE:524<br>**Salt bridge:** ARG:523; HIS:526
**Asperulosidic acid**	PIK3CA	2V1Y	-5.7	1.1387	**Hydrogen bond:** GLN:501; ARG:523; ASN:527<br>**Hydrophobic interaction:** TYR:504; TYR:508; ILE:524
**Caffeic acid**	PIK3CA	2V1Y	-5.7	6.2992	**Hydrogen bond:** ASN:564<br>**Hydrophobic interaction:** TYR:467; TYR:470<br>**Pi-pi stacking:** TYR:467
**p-Coumaric acid**	MAP4K1	7KAC	-5.6	1.6434	**Hydrogen bond:** CYS:94; ASP:155<br>**Hydrophobic interaction:** ALA:44; VAL:75; LEU:144<br>**Salt bridge:** LYS:33
**5-O-Feruloylquinic acid**	PIK3CA	2V1Y	-5.6	10.3889	**Hydrogen bond:** GLN:501; SER:505; HIS:526<br>**Hydrophobic interaction:** ILE:509; TYR:528

**p-Coumaric acid**	CTSK	5Z5O	-5.6	1.6357	**Hydrogen bond:** ILE:93; LYS:139<br>**Hydrophobic interaction:** TYR:92; VAL:227; PHE:311<br>**Salt bridge:** ARG:210
**Ferulic acid**	PIK3CA	2V1Y	-5.4	0.1767	**Hydrogen bond:** ASN:564<br>**Hydrophobic interaction:** TYR:467; TYR:470; LYS:567<br>**Salt bridge:** LYS:567
